# Supplementary material for: Youth’s exposure to and engagement with e-cigarette marketing on social media: a UK focus group study
Source: BMJ Open. 2023 Aug 23;13(8):e071270. doi: 10.1136/bmjopen-2022-071270 (PMC10450076; doi:10.1136/bmjopen-2022-071270)
Supplement: Supplementary data [file bmjopen-2022-071270supp001.pdf]

Appendix A: Interview topic guide

During the focus groups images were shown in a PowerPoint presentation. A series of images (e.g., Slides 2-8) were shown and changed at five-second intervals and once all images in that section were shown the group were then asked to discuss these based upon questions in the topic guide.

Part 1: Introduction/background

- First of all, thank you for taking part in this research.
- I'm [researcher name], a research assistant at the University of Glasgow with an interest in tobacco and e-cigarettes.

Key points for consent:

- Can I confirm that you have received the participant information sheet and signed and returned your consent form.
- Just to reiterate, your taking part is voluntary, and you are free to withdraw at any time without giving a reason.
- Your participation will be anonymous. Excerpts from the interview may be quoted verbatim in a report and a paper, but quotations will be anonymised.
- Non-anonymised interview recordings and transcripts will be destroyed securely upon completion of this research, but anonymised transcripts and consent forms will be stored securely by the University of Glasgow for a period of 10 years for the purposes of ensuring research integrity.
- Finally, the study has been approved by the Ethics Committee of the College of Social Sciences at the University of Glasgow.
- Reiterate that I am interested to hear their thoughts and experiences as things on social media are changing so quickly and they will know more than me.

Young people's use of social media

First of all, I am going to ask you about social media and what you use.

Show Slide 1 of the PowerPoint presentation.

Smith MJ, Hilton S. *BMJ Open* 2023; 13:e071270. doi: 10.1136/bmjopen-2022-071270

1. Do you recognise any of these logos?
    a. PROMPT: Can you tell me what each logo is?
    b. PROMPT: What do you know about each one?
2. Can you tell me about the social media platforms that you use?
    a. Why do you use those?
    b. In what way do you use those?
        i. PROMPT: How do you use them? E.g., 'liking', commenting, sharing?
        ii. PROMPT: What is the purpose of each?
        iii. PROMPT: Whether all are the same in the sense of what you can do on them or whether some are different?
    c. What social media platforms do you like and dislike?
        i. PROMPT: Why?
    d. On a daily basis, how long do you tend to spend on social media?
3. What do you think the term 'social media influencer' means?
    a. What do social influencers do?
    b. How do you know that someone is a social influencer?
    c. Can you tell me of any social media influencers whom you like and dislike?
    d. What products have you seen influencers advertising?
        i. PROMPT: Clothing, makeup?
    e. How do you think they pick the products that they advertise?
    f. Have you ever bought or used products based on what you have seen on social media?
        i. PROMPT: What type of products were those?
    g. Would you ever like, comment on or share an influencer's image if they were advertising something?

E-cigarettes on social media

Now I am going to show a few slides on PowerPoint and after we have been through them, we will chat through what you have seen.

Smith MJ, Hilton S. *BMJ Open* 2023; 13:e071270. doi: 10.1136/bmjopen-2022-071270

Show Slides 2-8 of the PowerPoint presentation.

Eight images of social media influencers and user-generated e-cigarette content were shown to participants. We have not included the images used as this would be considered a copyright and ethics issue.

1. What can you remember from the images that you just saw?
2. Do any of these images grab your attention?
    a. PROMPT: Why?
    b. PROMPT: What do you think about the use of colour in the images?
    c. PROMPT: What do you think about the number of likes each picture has?
    d. PROMPT: What do you think about the caption under the images?
3. Did the images have anything in common?
    a. If so, what?
4. Did you recognise anyone in the images?
    a. If so, who?
5. Why do you think these images have been posted?
6. What do you think is the purpose of the images?
7. What do you think the image is trying to say?
    a. PROMPT: How do you know this?
8. Who do you think these images are targeted at?
    a. PROMPT: Why do you say that?
9. Do you think these images are selling a product?
    a. PROMPT: How can you tell if a product is being promoted or not?
    b. PROMPT: How do you know which product(s) are being promoted?
10. Who do you think has posted the images I showed you?
    a. Do you think that person is being paid to post the image or not?
    b. How can you tell if someone is being paid or not?
    c. Why do you think influencers would want to advertise e-cigarette products?
    d. Why do you think companies would pay influencers to advertise their products?

Show slides 9-12 of the PowerPoint presentation.

Smith MJ, Hilton S. *BMJ Open* 2023; 13:e071270. doi: 10.1136/bmjopen-2022-071270

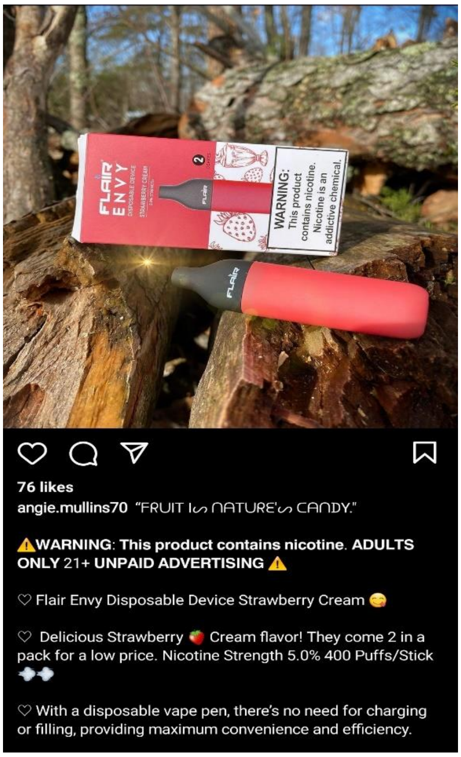

Slide 9

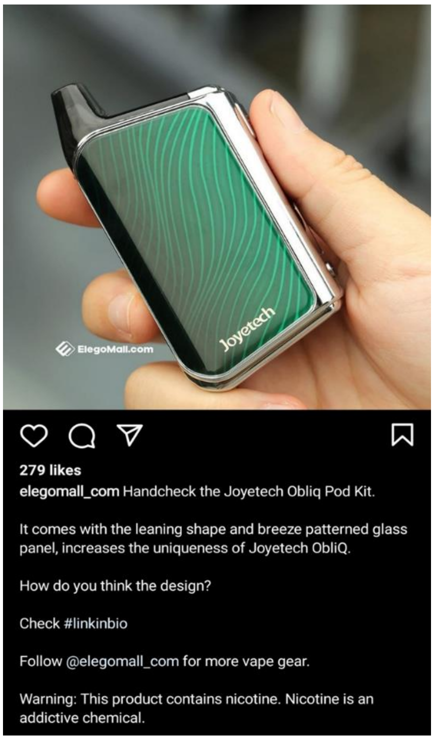

Slide 10

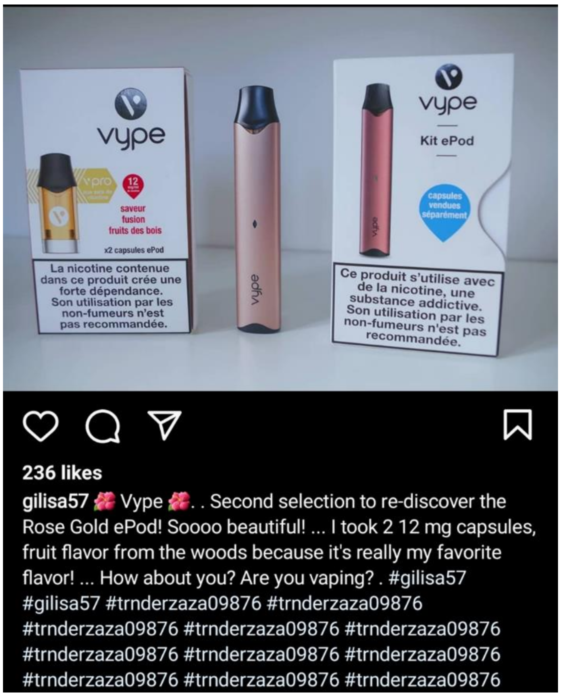

Slide 11

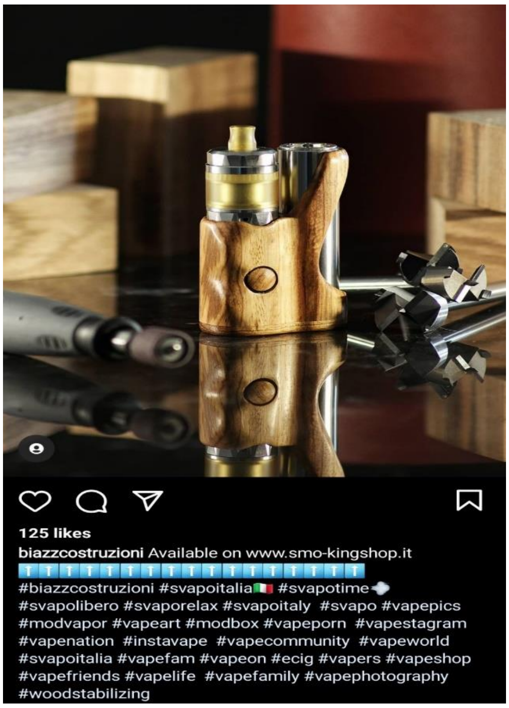

Slide 12

Smith MJ, Hilton S. *BMJ Open* 2023; 13:e071270. doi: 10.1136/bmjopen-2022-071270

1. What can you remember from the images that you just saw?
2. Do any of these images grab your attention?
    a. PROMPT: Why?
    b. PROMPT: What do you think about the use of colour in the images?
    c. PROMPT: What do you think about the number of likes each picture has?
    d. PROMPT: What do you think about the caption under the images?
3. Did the images have anything in common?
    a. If so, what?
4. Do any of these images remind you of any other products?
    a. PROMPT: Why?
5. Why do you think these images have been posted?
6. What do you think is the purpose of the images?
7. What do you think the image is trying to say?
    a. PROMPT: How do you know this?
8. Who do you think these images are targeted at?
    a. PROMPT: Why do you say that?
9. Why do you think e-cigarettes have been designed this way?

Show slides 14-18 of the PowerPoint presentation.

Smith MJ, Hilton S. *BMJ Open* 2023; 13:e071270. doi: 10.1136/bmjopen-2022-071270

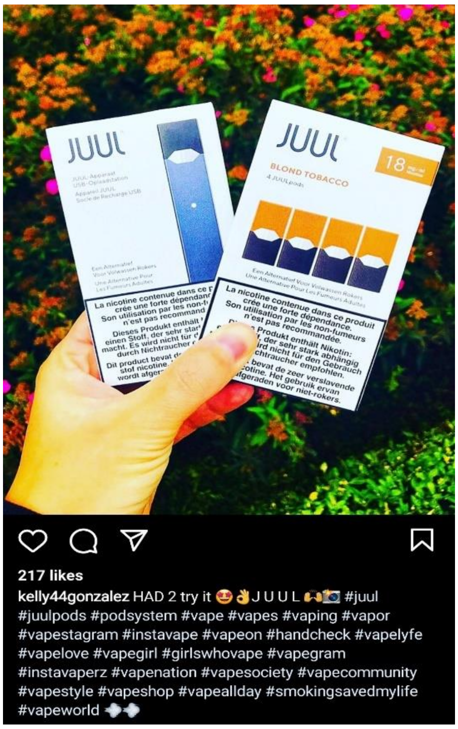

Slide 14

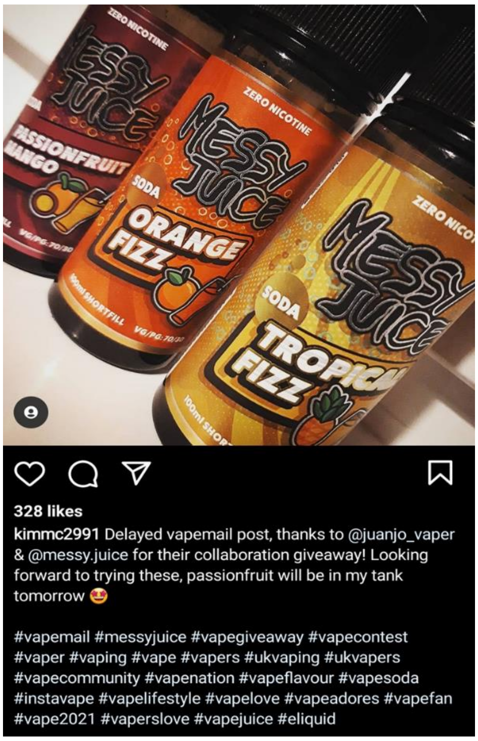

Slide 15

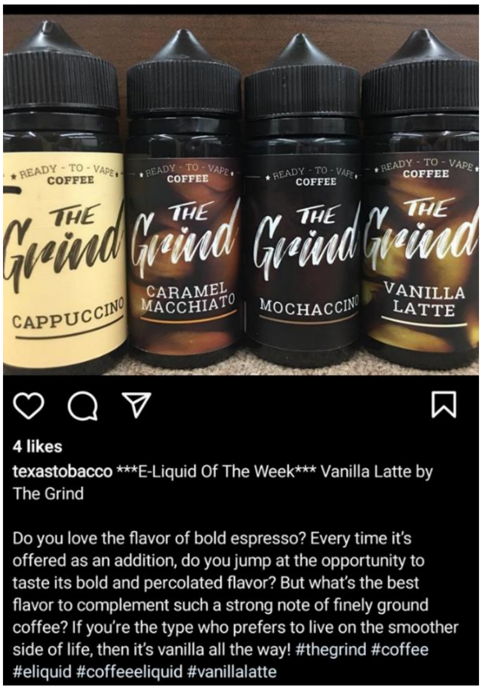

Slide 16

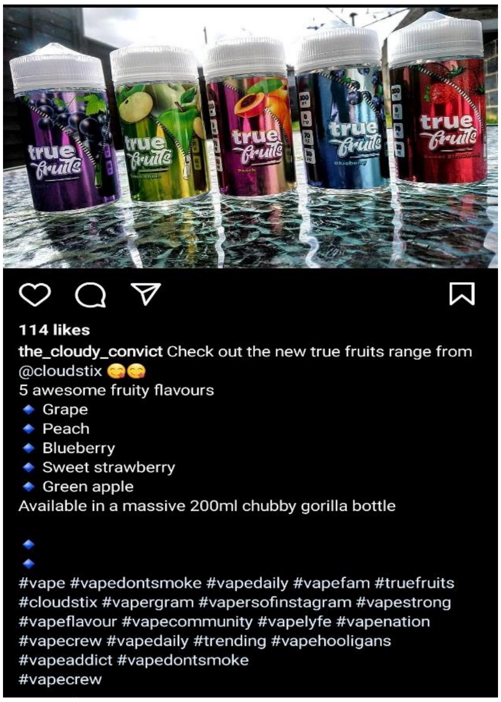

Slide 17

Smith MJ, Hilton S. *BMJ Open* 2023; 13:e071270. doi: 10.1136/bmjopen-2022-071270

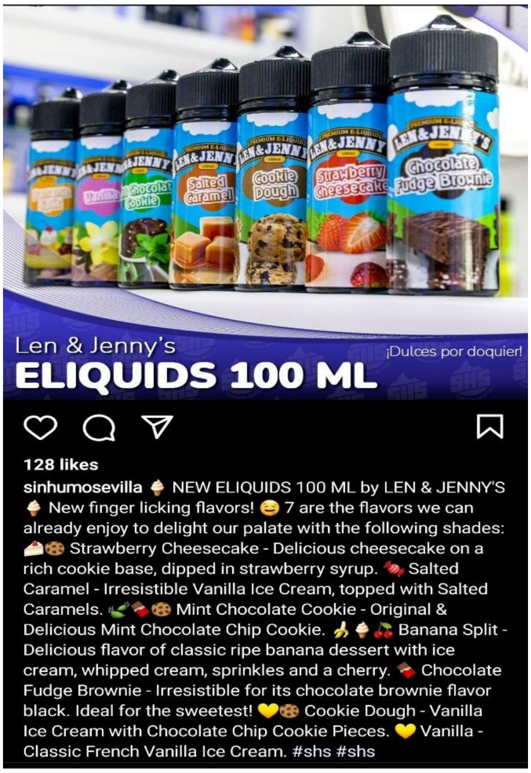
LEN & JENNY'S
Salted Caramel
Cookie Dough
Strawberry Cheesecake
Chocolate Fudge Brownie
Len & Jenny's
ELIQUIDS 100 ML
¡Dulces por doquier!
128 likes
sinhumosevilla NEW ELIQUIDS 100 ML by LEN & JENNY'S New finger licking flavors! 7 are the flavors we can already enjoy to delight our palate with the following shades: Strawberry Cheesecake - Delicious cheesecake on a rich cookie base, dipped in strawberry syrup. Salted Caramel - Irresistible Vanilla Ice Cream, topped with Salted Caramels. Mint Chocolate Cookie - Original & Delicious Mint Chocolate Chip Cookie. Banana Split - Delicious flavor of classic ripe banana dessert with ice cream, whipped cream, sprinkles and a cherry. Chocolate Fudge Brownie - Irresistible for its chocolate brownie flavor black. Ideal for the sweetest! Cookie Dough - Vanilla Ice Cream with Chocolate Chip Cookie Pieces. Vanilla - Classic French Vanilla Ice Cream. #shs #shs

Slide 18

1. What can you remember from the images that you just saw?
2. Do any of these images grab your attention?
   a. PROMPT: Why?
   b. PROMPT: What do you think about the colour of the products?
   c. PROMPT: What do you think about the number of likes each picture has?
   d. PROMPT: What do you think about the caption under the images?
3. Did the images have anything in common?
      i. If so, what?
4. Do any of these images remind you of any other products?
   a. PROMPT: Why?
5. Do you think these images are selling a product?
   a. Do you know what the products are that are shown in the images?
      i. If yes, how do you know that?
6. Why do you think these images have been posted?
7. What do you think is the purpose of the images?
8. What do you think the image is trying to say?
   a. PROMPT: How do you know this?
9. Who do you think these images are targeted at?
   a. PROMPT: Why do you say that?
10. What do you think of the flavours shown?
11. Do you see some flavours as more appealing compared to others?
   a. PROMPT: What ones and why?
12. Do you think there are any flavours that are more appealing to youths compared to adults?
   a. PROMPT: What ones and why?

<u>Personal experiences</u>

I am now moving on to ask about your own experiences and of friends and other people.

1. Have you seen any information about e-cigarettes on social media platforms?
   a. What kind of information was it?
      i. PROMPT: Why do you think the images were posted?
      ii. PROMPT: What do you think the images were trying to show/tell you?
2. Do friends or anyone you follow on social media post or share messages about e-cigarettes?
   a. How does that make you feel about them?
   b. Why do you think they post or share these messages?
   c. If seeing a friend or someone you know post about e-cigarettes on social media, would it make you want to share images or messages?

Smith MJ, Hilton S. *BMJ Open* 2023; 13:e071270. doi: 10.1136/bmjopen-2022-071270

d. Out of interest have you or your friends ever posted on social media about e-cigarettes?

Warnings on social media posts

1. Play the video (short clip) https://www.youtube.com/watch?v=tk9SQDH_ZNo
    a. Did you notice anything about the short clip I just showed you?
    b. Can you describe what you remember from the clip I just showed you?
    c. IF NECESSARY PROMPT: Did anyone notice the warning notice?
    d. PROMPT: What did it say?
    e. PROMPT: What did you think about it?
2. Replay the video pointing out the warning.
    a. What do you think about the length of time that the warning was shown for?
        i. How easy or difficult was it to notice the warning?
3. Why do you think there are warnings on posts/videos that show e-cigarette products?
4. Do you think there should or should not be warnings on posts/videos showing e-cigarette products?
    a. PROMPT: Why?

Final remarks

1. Have you ever noticed the marketing/promotion of e-cigarette products before or not?
2. How do you think about the marketing/promotion of e-cigarette products on social media?

Close interview

- Is there anything else you would like to add, that we haven't already talked about?
- Thank you very much for taking part.

Smith MJ, Hilton S. *BMJ Open* 2023; 13:e071270. doi: 10.1136/bmjopen-2022-071270
