# Supplementary material for: Youth’s exposure to and engagement with e-cigarette marketing on social media: a UK focus group study
Source: BMJ Open. 2023 Aug 23;13(8):e071270. doi: 10.1136/bmjopen-2022-071270 (PMC10450076; doi:10.1136/bmjopen-2022-071270)
Supplement: Author's manuscript [file bmjopen-2022-071270.draft_revisions.pdf]

BMJ Open is committed to open peer review. As part of this commitment we make the peer review history of every article we publish publicly available.

When an article is published we post the peer reviewers' comments and the authors' responses online. We also post the versions of the paper that were used during peer review. These are the versions that the peer review comments apply to.

The versions of the paper that follow are the versions that were submitted during the peer review process. They are not the versions of record or the final published versions. They should not be cited or distributed as the published version of this manuscript.

BMJ Open is an open access journal and the full, final, typeset and author-corrected version of record of the manuscript is available on our site with no access controls, subscription charges or pay-per-view fees (http://bmjopen.bmj.com).

If you have any questions on BMJ Open's open peer review process please email info.bmjopen@bmj.com

BMJ Open

Youth's exposure to e-cigarette marketing on social media: a focus group study

Journal:	*BMJ Open*
Manuscript ID	bmjopen-2022-071270
Article Type:	Original research
Date Submitted by the Author:	20-Dec-2022

Complete List of Authors:	Smith, Marissa; University of Glasgow, MRC/CSO Social and Public Health Sciences Unit<br>Hilton , Shona; University of Glasgow, MRC/CSO Social and Public Health Sciences Unit

Keywords:	Health policy < HEALTH SERVICES ADMINISTRATION & MANAGEMENT, PUBLIC HEALTH, QUALITATIVE RESEARCH

SCHOLARONE™
Manuscripts

BMJ

I, the Submitting Author has the right to grant and does grant on behalf of all authors of the Work (as defined in the below author licence), an exclusive licence and/or a non-exclusive licence for contributions from authors who are: i) UK Crown employees; ii) where BMJ has agreed a CC-BY licence shall apply, and/or iii) in accordance with the terms applicable for US Federal Government officers or employees acting as part of their official duties; on a worldwide, perpetual, irrevocable, royalty-free basis to BMJ Publishing Group Ltd ("BMJ") its licensees and where the relevant Journal is co-owned by BMJ to the co-owners of the Journal, to publish the Work in this journal and any other BMJ products and to exploit all rights, as set out in our licence.

The Submitting Author accepts and understands that any supply made under these terms is made by BMJ to the Submitting Author unless you are acting as an employee on behalf of your employer or a postgraduate student of an affiliated institution which is paying any applicable article publishing charge ("APC") for Open Access articles. Where the Submitting Author wishes to make the Work available on an Open Access basis (and intends to pay the relevant APC), the terms of reuse of such Open Access shall be governed by a Creative Commons licence – details of these licences and which Creative Commons licence will apply to this Work are set out in our licence referred to above.

Other than as permitted in any relevant BMJ Author's Self Archiving Policies, I confirm this Work has not been accepted for publication elsewhere, is not being considered for publication elsewhere and does not duplicate material already published. I confirm all authors consent to publication of this Work and authorise the granting of this licence.

For peer review only

Youth's exposure to e-cigarette marketing on social media: a focus group study

Marissa J. Smith[1*] and Shona Hilton[1]

[1] MRC/CSO Social and Public Health Sciences Unit, University of Glasgow, Glasgow, G3 7HR, UK

*Corresponding author

Email: marissa.smith@glasgow.ac.uk Tel: (+44) 0141 353 7500

Keywords: Health policy, qualitative research, public health

Word count: 3575

For peer review only

ABSTRACT

Objective: To explore young people's perceptions of social media marketing of e-cigarettes.

Design: Semi-structured discussion groups.

Subjects: Twenty focus groups with 82 young people aged 11-16 living in the Central belt of Scotland.

Methods: Youths were asked about smoking and vaping behaviours, social media use, vaping advertisement exposure, and were shown illustrative examples of social media content (e.g., images and videos) about different messages, presentations, and contextual features. Transcripts were imported into NVivo 12, coded thematically and analysed.

Results: Youths highlighted a variety of tactics e-cigarette companies use, including influencer or celebrity endorsement, attractive youth flavours, bright colours and emotional appeal to advertise and promote their products directly to young people. Social media influencers who advertise e-cigarettes were described as portraying e-cigarettes as 'cool' and 'fashionable' to entice viewers to try the products. Youths considered that there is a need for more restrictions on social media content to protect youths while also still allowing smokers to purchase them as a cessation device.

Conclusions: Our study provides worrying evidence that highlights that the e-cigarette industry is using previously employed tactics similar to the tobacco industry to advertise and promote its products on social media. These findings suggest the growing need for governments to work together to develop and implement policies to restrict the advertising and marketing of e-cigarettes on social media.

For peer review only

Strengths and limitation of this study

- Methodology includes semi-structured interviews with 82 youths aged 11-16 facilitating understandings of youths are exposure to and interaction with e-cigarette marketing on social media.
- This study is one of the first in-depth studies to explore youth's responses to and engagement with e-cigarette marketing on social media with a focus on the rise in user-generated and influencer content.
- Quantitative thematic analysis of the data allows depth of opinions but cannot offer predictions about the frequency of specific opinions with a wider population.
- This research offered new insights into an under-research area, but the complexity of the social media environment is such that implementing comprehensive policies to restrict the advertising and marketing of e-cigarettes on social media is challenging.

For peer review only

For peer review only - http://bmjopen.bmj.com/site/about/guidelines.xhtml

INTRODUCTION

Marketing strategies used by electronic cigarette (e-cigarette) companies have been associated with the uptick in e-cigarette use (vaping) among youth [1]. Many studies have found that the promotion of e-cigarettes through various channels (e.g., television (TV), social media influencers) has lent to increased positive perceptions of vaping and intentions to use vaping products and contributed to e-cigarette uptake among youth [2-4].

In the UK, the advertising of e-cigarettes is regulated through Article 20(5) of the revised European Union (EU) Tobacco Products Directive (2014/40/EU) (TPD), [5] which was transposed into UK law by the Tobacco and Related Products Regulations (TRPR) 2016 [6]. The TPD prohibited the advertising of nicotine-containing e-cigarettes (unless licensed as medicines) in channels with potential cross-border impact (i.e. channels that show adverts or sponsored events that originate from non-EU countries in EU countries), including TV, radio, newspapers, magazines, and sponsorship [7]. Online advertising was also prohibited under the TPD, although the regulations left scope for marketers to retain websites containing factual information about e-cigarette products. As there are currently no medicinally licensed nicotine vaping products in the UK, the prohibitions apply to all nicotine-containing e-cigarette products on the market. The TRPR requirements for e-cigarette advertising were set out in 2017 and are enforced by the Committee of Advertising Practice (CAP) – a self-regulatory body of organisations representing advertising, direct marketing, media businesses, and sales promotion endorsed and administered by the independent Advertising Standards Authority (ASA) [8, 9]. Although Rule 22.12 of the ASA CAP code prohibits advertising in online media, social media content for e-cigarettes is permitted in “non-paid-for space online under the marketer’s control, provided claims are factual rather than promotional” [10]. Promotional content includes descriptive language that goes beyond objective, factual claims, significant imagery that is not related to the product, and health claims (e.g. that e-cigarettes are safer or healthier than tobacco) [10].

Social media marketing includes promotions by means of paid digital advertising, via compensated ‘influencers’ (individuals with large followings who are paid to advertise brands and products), and on brand pages that companies can create for free and use to share content [11]. Most adolescents use

For peer review only - http://bmjopen.bmj.com/site/about/guidelines.xhtml

visual-based social media daily. User-generated and influencer marketing content on social media represents a key influence on young people's understanding of products, including e-cigarettes [12]. E-cigarettes are promoted creatively through social media, with well-designed features including colours, flavour variations, emotional appeal, incentives (such as price promotions and discount vouchers), and even celebrity endorsements [13]. Research has shown that online marketing that leads to exposure to e-cigarette advertising, including where it is concealed as information or recommendations from peers, can increase the likelihood of vaping in individuals, including among youths [14-16]. E-cigarette companies have been promoting their products on social media in an attempt to expand their market [17, 18]. In addition, e-cigarette companies use social media platforms to promote e-cigarettes and create a socially attractive vaping image [19].

Given the potential influence of social media marketing on young people, it is important to understand the nature, extent, and impact of e-cigarette marketing via social media. This study, therefore, aims to explore youth's responses to, and engagement with e-cigarette advertising on social media, including user-generated and influencer content.

METHODS

We conducted 20 focus groups in Scotland between March and May 2022. Focus groups included between three and five participants (a total of 82 participants). Purposive sampling was used to recruit a diverse sample of youths in terms of sex, socio-economic background, smoking status, and vaping status. Eleven groups were recruited through youth workers in local youth organisations. These gatekeepers handed out information sheets and helped achieve the sampling frame in terms of youth demographics and experiences concerning traditional cigarettes and e-cigarettes. The three organisations that helped with participant recruitment worked specifically with young people from disadvantaged backgrounds in urban areas. This recruitment strategy resulted in the inclusion of a range of participants from more affluent and more deprived backgrounds and with experiences of smoking and vaping. Seven groups were recruited through the Schools Health and Wellbeing

For peer review only

Improvement Research Network (SHINE) Newsletter which is distributed monthly to over 500 schools in Scotland. The remaining two groups were recruited via personal networks directly by MS. Focus group discussions were facilitated to allow the research team to explore how opinions about online advertising and marketing of e-cigarettes. Friendship groups of 3–5 participants were used to facilitate in-depth insights and promote participant interaction. Each participant was given a £20 shopping voucher as compensation for their time.

Prior to the start of the focus groups, participants completed a short anonymous questionnaire about their age, sex, postcode, smoking, and e-cigarette use status. For both traditional cigarettes and e-cigarettes, the questionnaire asked participants to specify whether they had tried or used them in the past or were using them at the time of the study. Based on a review of the literature a topic guide was developed which covered three key areas, including use of social media, perceptions of social media influencers versus user-generated e-cigarette content on social media, and views on the marketing of e-cigarettes on social media.

Images of different types of e-cigarettes (‘tanks’, disposables, and pod devices) and e-liquids posted by social media influencers and general users were used as conversation starters. Group discussions were facilitated by MS. Ten of the groups were conducted online using Microsoft Teams and ten of the groups were conducted face-to-face. Of these, one of the groups was conducted on the youth organisation’s premises, and the other nine were conducted at the school, with representatives of the youth organisation present. Groups lasted between 40 and 66 minutes. Field-notes reflecting on the focus group and individual issues discussed were written up for each group. All focus groups were audio recorded (with participants' permission) and transcribed verbatim. Each transcript was imported into NVivo 12, coded independently, cross-checked, and analysed by MS and SH. Contradictory cases and group dynamics were discussed, making use of transcripts and field notes. Ethical approval for the study was obtained from the University of Glasgow's Medical and Veterinary Life Sciences Ethics Committee (reference 200210034).

Patient and Public Involvement

None.

For peer review only

RESULTS

Participant characteristics

Eighty-two youths aged 11–16 years participated (47 females (57%) and 35 males (43%)), representing diversity in sociodemographic characteristics and smoking-related behaviours. Age distribution within the sample was skewed slightly towards 14–15-year-olds, with 14-year-olds making up the largest subgroup (n= 24). While the majority of participants did not currently smoke or use e-cigarettes, the sample included 10 smokers and 18 youths who used e-cigarettes. Table 1 describes the focus group composition and participants in more detail and Table 2 summarises smoking and e-cigarette use among the sample.

For peer review only

Group	Area	Sex	Age	Cigarette Smoker	E-cigarette use
1	Affluent	Female	13-15	Never	Never
2	Affluent	Female	14-15	Never	Mixed – Never (4) / Tried (1)
3	Affluent	Female	13-16	Never	Mixed – Never (2) / Tried (1)
4	Deprived	Mixed – Male (3)/Female (2)	12-15	Mixed – Never (3) / Current (2)	Mixed – Never (3) / Tried (1)/ Current (1)
5	Deprived	Mixed – Male (1)/ Female (4)	14-16	Mixed – Never (2)/ Tried (2)/ Current (1)	Mixed – Never (2)/ Tried (2)/ Current (1)
6	Deprived	Male	12-15	Never	Never
7	Deprived	Male	16	Current	Current
8	Affluent	Mixed – Male (2)/Female (3)	14	Never	Never
9	Deprived	Male	16	Mixed – Tried (1)/ Current (2)	Current
10	Deprived	Mixed – Male (4)/Female (1)	14-15	Mixed – Never (3)/ Tried (1)/ Current (1)	Mixed – Never (3)/ Tried (1)/ Current (1)
11	Deprived	Mixed – Male (3)/Female (2)	13-16	Mixed – Never (2)/ Tried (2)/ Current (1)	Mixed – Never (1)/ Current (4)
12	Affluent	Mixed – Male (2)/Female (1)	15-16	Tried	Mixed – Tried (2)/ Current (1)
13	Affluent	Female	13-16	Never	Never
14	Deprived	Mixed – Male (1)/Female (3)	11-12	Never	Never
15	Deprived	Mixed – Male (3)/Female (1)	11-12	Never	Never
16	Deprived	Mixed – Male (2)/Female (2)	11-12	Never	Never
17	Deprived	Female	14-16	Mixed – Never (4)/ Tried (1)	Mixed – Never (1)/ Tried (1)/ Current (3)
18	Deprived	Male	13-16	Never	Never
19	Deprived	Female	14	Never	Mixed – Tried (2)/ Current (1)
20	Affluent	Female	15-16	Never	Tried (3)

Table 1: Focus group location, participants, and their cigarette smoking and e-cigarette use.

For peer review only

	E-cigarette use											
	Never			Tried			Current			Total		
**Cigarette smoker**	**n**	**(col %)**	**(row %)**	**n**	**(col %)**	**(row %)**	**n**	**(col %)**	**(row %)**	**n**	**(col %)**	**(row %)**
**Never**	51	98.1%	82.3%	7	58.3%	11.3%	4	22.2%	6.5%	62	75.6%	100.0%
**Tried**	1	1.9%	10.0%	4	33.3%	40.0%	5	27.8%	50.0%	10	12.2%	100.0%
**Current**	0	0.0%	0.0%	1	8.3%	10.0%	9	50.0%	90.0%	10	12.2%	100.0%
**Total**	52	1	92.3%	12	100.0%	14.6%	18	100.0%	22.0%	82	100.0%	100.0%

Table 2: E-cigarette use according to cigarette smoking.

For peer review only

Perceptions of advertising tactics used by the e-cigarette industry to target youths

Youths discussed three ways (influencer or celebrity endorsement, flavourings and emotional appeal) e-cigarette companies market their products to target young people.

Influencer or celebrity endorsements

During the focus groups, participants frequently discussed the prevalence of social media influencers advertising e-cigarette products. When discussing why e-cigarette and/or tobacco companies would want to use social media influencers to advertise their products, participants highlighted the popularity of social media and how this would increase the reach of the adverts.

> *"I guess companies would use them [influencers] because the company may have approached them to advertise the product and they know they might have a huge following on the platform, so they might influence some more people to buy the products." (Male, current smoker, current vaper)*

> *"More people are on social media nowadays. So, it's better that they [vaping/tobacco companies] use influencers to advertise their products compared to say in newspapers, 'cause you don't see people reading newspapers anymore." (Male, never smoker, never vaper)*

Participants reported seeing marketing and advertising of e-cigarettes on social media and that they were portrayed as 'cool' and 'fashionable' by social media influencers.

> *"If an influencer posts it, a lot of people see it and be like, that's the trend now, it's a cool thing to do, so I want to take part in that." (Female, never smoker, tried vaping)*

For peer review only

"It's like they're showcasing them. It's not just like it's in the background or whatever, it's not a normal pose to have them in, it's like they're showcasing them and that they are a fashionable thing to carry or to have." (Female, never smoker, never vaper)

Participants discussed that this would influence the viewer to try the product, thus increasing the prevalence of use.

"People get tempted easily by seeing the post. Like if they saw some influencers trying that stuff [e-cigarettes], people get tempted quickly to try that stuff, because then that person is trying it so the like the kids would think this is something that I should also try that stuff like that, that's the point." (Male, current smoker, current vaper)

"When you see what an influencer is, most people think oh if they're doing that, then it's cool, so it like, it's making more people want to buy the vapes, and stuff." (Female, never smoker, tried vaping)

Several participants discussed how social media posts make it too easy for underage youths to purchase and hide the fact you are buying e-cigarette products from websites.

"I've seen a thing on TikTok like, they [influencers] show you them putting them [e-cigarettes] in the wee boxes and all that, or you could put them in secret packaging like behind the lashes. Like you can order it off their website and they'll hide it in the packaging, they put a few bits of sweeties on top of your vapes so your mum doesn't see it." (Female, tried smoking, current vaper)

Using sweet and fruity flavours to appeal to youths

For peer review only - http://bmjopen.bmj.com/site/about/guidelines.xhtml

Participants discussed the variety of flavours of e-cigarettes available, highlighting that the sweet and fruity flavours are particularly attractive to youths.

> *"'Cause younger people, like us would think that the flavoured ones look nice so they would try then and then they'd start vaping." (Male, never smoker, never vaper)*

> *"I think the type of ice cream flavours or sweetie ones are targeted at younger people because most people in their 30s and 40s would probably use the tobacco one or the coffee ones." (Male, current smoker, current vaper)*

> *"The ones that stood out to me the most were the Ben and Jerry's and fruit ones compared to the tobacco ones. I feel like, if I was going to buy them, I would buy them 'cause they are attractive and are things I like." (Female, tried smoking, current vaper)*

Several participants stated that the variety of flavourings was to appeal to youths.

> *"I feel like no one really wants to taste tobacco, to be honest. But I think putting out there with like grape and blueberry flavourings are going to attract younger people to the marketplace. I think it's an intentional design and I feel like that is probably what it does attract youths." (Female, never-smoker, never vaper)*

Using vibrant colours to attract youth attention

In addition, participants spoke about the presentation of e-cigarette products on social media, for example, the use of vibrant colours and filters.

For peer review only

"You can tell by the picture they're promoting them. Because if it was not promoting them you would have like a red colour, it's like a stop, it's kind of like a no colour. But they are using really positive colours, like pink and green. It makes you stop and look at it to see what it's [the social media post] is about." (Male, never smoker, never vaper)

"It was really bright colours and they had bright filters on them so it is brighter. If you were scrolling past then gives you a bright colour, you'd go back and see it. You'd be like, oh what's that? You'd be, like, drawn to it. […] I think young people like us would be drawn to that, well I would." (Female, never smoker, never vaper)

Connecting emotionally with youths

Participants discussed the emotional connections and positive portrayal of e-cigarettes on social media, stating that individuals in the social media posts 'looked happy' to be using the products.

"I think the person who posted this product, I think he or she was trying to emphasise the fact that these products are good because they look happy to be advertising it. So, you should try them and like they were not promoting vaping negatively or that it is bad at all. I think they were trying to sell the aesthetic idea of this product." (Male, current smoker, current vaper)

"It's the way they're all holding them, they looked quite passionate with a smile on their face. A big cheesy smile." (Male, current smoker, current vaper)

For peer review only - http://bmjopen.bmj.com/site/about/guidelines.xhtml

Participants highlighted the use of emojis in e-cigarette social media posts. Emojis (icons used to express an idea or emotion) and emoji sequencing (the use of two or more emojis that form a conceptual rhetorical unit) [20] are used to enhance the message meaning [21, 22]. When asked if this would affect their interaction with social media posts that included emojis, participants stated that they would be more inclined to read the post, rather than scrolling past.

> *"If you see it in colours, like emojis, you're like, oh I wonder what that is. You'd stop scrolling. 'Cause normally captions is just, like, text and a black background or something like that." (Female, never smoker, never vaper)*

> *"The wee emojis they would draw my attention, because of how brightly coloured they are. And I think that's what they're trying to do, like, it'll draw your attention." (Female, never smoker, never vaper)*

Perceptions of what can be done to limit e-cigarette marketing on social media

Participants had divergent views on the marketing and advertising of e-cigarettes on social media. Several participants stated that marketing and advertising should not be allowed as youths, including those of a similar age, could access this type of content and it may encourage them to try e-cigarettes.

> *"Advertising them [e-cigarettes] on social media is s a bad thing because younger people might see it and then try it." (Male, never smoker, never vaper)*

> *I don't think it [advertising of e-cigarettes] should be allowed, just because of the younger community on it [social media]. And then if they are seeing them, they might end up doing it, getting addicted and end up dying." (Male, tried smoking, tried vaping)*

For peer review only - http://bmjopen.bmj.com/site/about/guidelines.xhtml

But participants also acknowledged that controlling what is posted on social media is 'tricky' and 'difficult' and that prohibiting e-cigarette advertising may limit their role as cessation devices by smokers. Those participants suggested instead of prohibiting the marketing and advertising of e-cigarettes, warnings (such as age and health warnings) or other restrictive measures (such as parental controls or age restrictions) could be imposed on these types of social media posts.

"I think there needs to be age restrictions on the posts or parents could control what their child sees." (Female, never smoker, never vaper)

DISCUSSION

E-cigarettes have become increasingly popular and visible in public life, particularly on social media. Our qualitative thematic analysis of youths' perceptions of and engagement with e-cigarette advertising on social media highlighted a public health concern. Similar to previous research [23-25], youths in our study highlighted the tactics used by the e-cigarette industry to promote and advertise e-cigarettes to underage populations through influencer and celebrity endorsements, variety of flavours and using promotional endorsements. Youths discussed the positive portrayal of e-cigarettes by social media influencers, and they were enticing the viewer to buy the product. Youths highlighted that e-cigarette and/or tobacco companies use social media influencers to advertise their products on social media and by adverting on social media platforms, they will increase the reach of the adverts to a wider audience. We found that youths had divergent views on e-cigarette marketing on social media. Several participants stated that e-cigarette advertising on social media should be prohibited, citing the high prevalence of youths on social media who could access the content. While others suggested a balanced approach to regulating advertising on social media so that they can still be advertised as cessation devices by smokers. Youths suggested that all adverts should contain warnings (such as age

For peer review only - http://bmjopen.bmj.com/site/about/guidelines.xhtml

and health warnings) or other restrictive measures (such as parental controls or age restrictions) to protect youths and non-users.

Previous studies which examined e-cigarette-related social media [26-30] found that the vast majority of the content depicted positive attitudes towards vaping, while negative characterisations were mostly absent. Our study found that youths had similar experiences when exposed to e-cigarette content on social media. Consequently, there is a risk that youths will be exposed to, and possibly engage with, content that promotes vaping while staying uninformed about the negative aspects, including potential health harms. Our study adds to existing concerns about youths' perceptions of e-cigarettes as cool, fashionable products [31-34], by considering the advertising of the products by influencers on social media platforms, frequently accessed by youths. The use of social media influencers to promote products and celebrity endorsements encourage their followers to share and interact with their content, and ultimately purchase their products. Social media influencers often collaborate with multiple industries (e.g., fashion, and beauty products) in addition to e-cigarette products/brands. These influencers could potentially expose their non-e-cigarette-focused audience (including non-users of e-cigarettes) to e-cigarette content [35]. Therefore, these influencers could be considered an even higher risk for youth compared to influencers who post exclusively about e-cigarettes. This is problematic and concerning as most e-cigarette brands claim that their advertising and promotional content is meant to target current cigarette smokers to help them switch to e-cigarettes, [36, 37] rather than people who do not use nicotine at all.

Before plain packaging, the tobacco industry successfully used colourful packaging to represent its brand and increase the appeal of smoking [38-40]. The marketing tactics used by the tobacco industry were so effective that several e-cigarette manufacturers have used the same trade secrets to advertise their products [41-43]. Research has shown the plethora of bright-coloured packaging, eye-catching designs, and variety of flavourings available all appeal to youths and may result in experimentation of the products [44-49].

Previous research has illustrated YouTube viewers' exposure to e-cigarettes (as well as alcohol and tobacco) while watching videos on the platform [50]. Our study adds to this by illustrating the

For peer review only

potential mechanisms by which exposure to e-cigarette advertising on social media platforms may influence perceptions and resultant trial/use of the products. While restrictions on mass media marketing of e-cigarettes are increasingly considered internationally, social media platforms are more difficult to manage because user-generated content (in particular) will not be covered by incoming regulations. Our study findings resonate with previous research, which found that youths were easily able to purchase e-cigarettes from the internet due to the absence of age-verification measures used by internet vendors [51, 52].

As with all research, our study has some limitations. First, and consistent with the qualitative design, the sample does not aim to be representative of UK youth, as our study focused on Scottish youths. However, we did have a diverse sample of both sexes. Secondly, the study's geographical remit has to be considered when interpreting the findings. The UK is considered an international leader in tobacco control policy. It is possible that participants' views may have been influenced by the UK's unique favourable policy approach to e-cigarettes and legal and socio-cultural context, including low smoking prevalence. Thirdly, the data was collected in different formats (online and face-to-face), and this may have influenced participants' responses. Two of the online groups were conducted in a classroom with a teacher present, and during seven face-to-face groups, a teacher/youth worker was present in the room. The presence of a teacher/youth worker may have influenced youth's willingness to answer questions and their responses. Finally, two of the groups were recruited through personal networks and this may have impacted the youth's responses.

The results from this study should provide guidance for future research. More research is needed to determine the most effective means to counter the favourable/positive aspects of e-cigarettes to reduce youths' interest in product trial and use. In addition, research on the prevalence of warning statements on e-cigarette-related social media posts and the impact this has on youth perceptions would be an important future direction to expand this work.

For peer review only

Considering that most youth access multiple social media platforms multiple times per day [53, 54] and that exposure to this marketing is related to use [55, 56], our findings indicate a significant public health concern. Given the findings presented by our study, there is a growing need for policymakers to develop comprehensive plans to build more robust measures to protect youths and to restrict the ability of marketers to reach youths with enticing social media content promoting e-cigarettes. In addition, social media platforms should consider implementing more robust measures, such as age restrictions and portraying the negative aspects of vaping, to ensure the prevention of vaping-related content targeted at underage users.

CONCLUSION

The tactics used on social media platforms for advertising and marketing e-cigarettes directly to youths raises concerns and a new generation of youths becoming addicted to nicotine. The results of this study highlight that the e-cigarette industry is using previously employed tactics similar to the tobacco industry to advertise and promote its products on social media. Most youths use social media daily and the depiction of products on social media represents a key influence on young people's understanding of products. These findings may highlight a priority for governmental policy to restrict the ability of marketers to reach youths with social media content promoting e-cigarettes.

Declarations

Authors' contributions: MS conducted all focus groups and performed all analysis. All authors contributed to the design of the topic guide. MS wrote the first draft of the manuscript. All authors read and approved the final manuscript.

Ethical approval: Ethical approval for the study was obtained from the University of Glasgow's Medical and Veterinary Life Sciences Ethics Committee (reference 200210034).

Competing interests: The authors declare that they have no competing interests.

Data sharing statement: All data relevant to the study are included in the article.

For peer review only - http://bmjopen.bmj.com/site/about/guidelines.xhtml

Funding: MS acknowledges funding from Cancer Research UK grant PPRCTAGPJT\100003. SH is funded by the Medical Research Council grant MC_UU_00022/1, the Chief Scientist Office of the Scottish Government Health Directorates grant SPHSU17, and Cancer Research UK grant PPRCTAGPJT\100003.

Role of the funding source: The funders of this study had no role in the study design, data analysis, data interpretation, or writing of the report. The corresponding author had full access to all of the data and final responsibility for the decision to submit it for publication.

REFERENCES

1. Hammond D, Wackowski OA, Reid JL, O'Connor RJ. Use of JUUL E-cigarettes Among Youth in the United States. Nicotine Tob Res. 2020;22(5):827-32.
2. Duke JC, Allen JA, Eggers ME, Nonnemaker J, Farrelly MC. Exploring Differences in Youth Perceptions of the Effectiveness of Electronic Cigarette Television Advertisements. Nicotine Tob Res. 2016;18(5):1382-86.
3. Farrelly MC, Duke JC, Crankshaw EC, Eggers ME, Lee YO, Nonnemaker JM, et al. A Randomized Trial of the Effect of E-cigarette TV Advertisements on Intentions to Use E-cigarettes. Am J Prev Med. 2015;49(5):686-93.
4. Hammond D, Reid JL, Burkhalter R, Rynard VL. E-cigarette Marketing Regulations and Youth Vaping: Cross-Sectional Surveys, 2017–2019. 2020;146(1).
5. European Commission. Tobacco Products Directive (2014/40/EU). European Commission; 2014. p. 1-38.
6. The Tobacco and Related Products Regulations 2016, 507. Sect. Part 2 (2016).
7. Stead M, Ford A, Angus K, MacKintosh AM, Purves R, Mitchell D. E-Cigarette Advertising in the UK: A Content Analysis of Traditional and Social Media Advertising to Observe Compliance with Current Regulations. Nicotine Tob Res. 2021;23(11):1839-47.
8. Advertising Standards Authority. About the ASA and CAP 2021 [Available from: https://www.asa.org.uk/about-asa-and-cap/about-regulation/about-the-asa-and-cap.html.
9. Advertising Standards Authority. The CAP Code: The UK Code of Non-broadcast Advertising and Direct & Promotional Marketing. London, UK: Advertising Standards Authority; 2016.
10. Advertising Standards Authority. Electronic Cigarettes: Factual vs. Promotional Claims 2018 [Available from: https://www.asa.org.uk/advice-online/electronic-cigarettes-factual-vs-promotional-claims.html.
11. O'Brien EK, Hoffman L, Navarro MA, Ganz O. Social media use by leading US e-cigarette, cigarette, smokeless tobacco, cigar and hookah brands. Tob Control. 2020;29(e1):e87-e97.
12. De Veirman M, Hudders L, Nelson MR. What Is Influencer Marketing and How Does It Target Children? A Review and Direction for Future Research. Front Psychol. 2019;10.
13. Zhu S, Sun J, Binnevie E, Cummins S, Gamst A, Yin L, et al. Four hundred and sixty brands of e-cigarettes and counting: implications for product regulation. Tob Control. 2014;23(3):3-9.
14. Chu KH, Colditz JB, Primack BA, Shensa A, Allem JP, Miller E, et al. JUUL: Spreading Online and Offline. J Adolesc Health. 2018;63(5):582-86.

For peer review only - http://bmjopen.bmj.com/site/about/guidelines.xhtml

15. Phua J, Jin SV, Hahm JM. Celebrity-endorsed e-cigarette brand Instagram advertisements: Effects on young adults' attitudes towards e-cigarettes and smoking intentions. J Health Psychol. 2017;23(4):550-60.
16. Amin S, Dunn AG, Laranjo L. Social Influence in the Uptake and Use of Electronic Cigarettes: A Systematic Review. Am J Prev Med. 2020;58(1):129-41.
17. Cobb NK, Brookover J, Cobb CO. Forensic analysis of online marketing for electronic nicotine delivery systems. Tob Control. 2015;24(2):128.
18. Amin S, Dunn AG, Laranjo L. Exposure to e-cigarette information and advertising in social media and e-cigarette use in Australia: A mixed methods study. 2020;213:108112.
19. Prochaska JJ, Pechmann C, Kim R, Leonhardt JM. Twitter=quitter? An analysis of Twitter quit smoking social networks. Tob Control. 2012;21(4):447-49.
20. Ge J. Emoji Sequence Use in Enacting Personal Identity. In: Liu L, White R, editors. WWW '19: Companion Proceedings of The 2019 World Wide Web Conference; New York, USA2019. p. 426-38.
21. Ge J, Gretzel U. Emoji rhetoric: a social media influencer perspective. 2018;34(15-16):1272-95.
22. Jacobson J, Hodson J, Mittelman R. Pup-ularity contest: The advertising practices of popular animal influencers on Instagram. 2022;174:121226.
23. Kostygina G, Tran H, Czaplicki L, Perks SN, Vallone D, Emery SL, et al. Developing a theoretical marketing framework to analyse JUUL and compatible e-cigarette product promotion on Instagram. Tob Control. 2022;10.1136/tobaccocontrol-2021-057120:tobaccocontrol-2021-057120.
24. Alpert JM, Jaisle A, Chen H. A content analysis of the promotional strategies employed by e-cigarette brands on Twitter. 2019;36(4):307-21.
25. Struik LL, Dow-Fleisner S, Belliveau M, Thompson D, Janke R. Tactics for Drawing Youth to Vaping: Content Analysis of Electronic Cigarette Advertisements. 2020;22(8):e18943.
26. Malik A, Li Y, Karbasian H, Hamari J, Johri A. Live, Love, Juul: User and Content Analysis of Twitter Posts about Juul. Am J Health Behav. 2019;43:326-36.
27. Allem JP, Dharmapuri L, Unger JB, Cruz TB. Characterizing JUUL-related posts on Twitter. Drug Alcohol Depend. 2018;190:1-5.
28. Cole-Lewis H, Pugatch J, Sanders A, Varghese A, Posada S, Yun C, et al. Social Listening: A Content Analysis of E-Cigarette Discussions on Twitter. J Med Internet Res. 2015;17(10):e243.
29. Ketonen V, Malik A. Characterizing vaping posts on Instagram by using unsupervised machine learning. Int J Med Inform. 2020;10.1016/j.ijmedinf.2020.104223.
30. Smith MJ, Buckton C, Patterson C, Hilton S. A scoping review and content analysis of YouTube and Instagram posts. in press.
31. de Andrade M, Angus K, Hastings G. Teenage perceptions of electronic cigarettes in Scottish tobacco-education school interventions: co-production and innovative engagement through a pop-up radio project. 2016;136(5):288-93.
32. Choi K, Fabian L, Mottey N, Corbett A, Forster J. Young adults' favorable perceptions of snus, dissolvable tobacco products, and electronic cigarettes: findings from a focus group study. Am J Public Health. 2012;102(11):2088-93.
33. Park E, Kwon M, Gaughan MR, Livingston JA, Chang Y-P. Listening to Adolescents: Their Perceptions and Information Sources About E-cigarettes. 2019;48:82-91.
34. Vogel EA, Ramo DE, Rubinstein ML, Delucchi KL, Darrow SM, Costello C, et al. Effects of Social Media on Adolescents' Willingness and Intention to Use E-Cigarettes: An Experimental Investigation. Nicotine Tob Res. 2020;10.1093/ntr/ntaa003.
35. Vassey J, Valente T, Barker J, Stanton C, Li D, Laestadius L, et al. E-cigarette brands and social media influencers on Instagram: a social network analysis. Tob Control. 2022;10.1136/tobaccocontrol-2021-057053:tobaccocontrol-2021-057053.
36. Dewhirst T. Co-optation of harm reduction by Big Tobacco. Tob Control. 2021;30(e1):e1.
37. Prakash S, Xu Y, Goldenson NI, Wissmann R, Gougelet R, Shiffman S. Transitions in Smoking among Adults Newly Purchasing the JUUL System. Am J Health Behav. 2021;45(3):546-62.
38. Wayne GF, Connolly GN. How cigarette design can affect youth initiation into smoking: Camel cigarettes 1983-93. Tob Control. 2002;11 Suppl 1(Suppl 1):I32-9.

For peer review only

39. Carpenter CM, Wayne GF, Connolly GN. Designing cigarettes for women: new findings from the tobacco industry documents. Addiction. 2005;100(6):837-51.
40. Anderson SJ, Glantz SA, Ling PM. Emotions for sale: cigarette advertising and women's psychosocial needs. Tob Control. 2005;14(2):127-35.
41. Campaign for Tobacco-Free Kids. Campaign for Tobacco-Free Kids. 2013. [cited 2022]. Available from: https://www.tobaccofreekids.org/blog/2013_10_02_ecigarettes.
42. Haugh M. University of Richmond. 2020. [cited 2022]. Available from: https://jolt.richmond.edu/2020/11/27/e-cigarettes-new-technology-but-old-advertising-strategy/.
43. Walley SC, Wilson KM, Winickoff JP, Groner J. A Public Health Crisis: Electronic Cigarettes, Vape, and JUUL. 2019;143(6).
44. Kong G, Morean ME, Cavallo DA, Camenga DR, Krishnan-Sarin S. Reasons for Electronic Cigarette Experimentation and Discontinuation Among Adolescents and Young Adults. Nicotine Tob Res. 2015;17(7):847-54.
45. Audrain-McGovern J, Strasser AA, Wileyto EP. The impact of flavoring on the rewarding and reinforcing value of e-cigarettes with nicotine among young adult smokers. Drug Alcohol Depend. 2016;166(1):263-67.
46. Czoli CD, Goniewicz M, Islam T, Kotnowski K, Hammond D. Consumer preferences for electronic cigarettes: results from a discrete choice experiment. Tob Control. 2016;25(1):30-36.
47. Zare S, Nemati M, Zheng Y. A systematic review of consumer preference for e-cigarette attributes: Flavor, nicotine strength, and type. 2018;13(3):e0194145.
48. Kalaji MNH. The Visual Is Worth Thousands of Words: Adult and Youth Perceptions of Visual Cues in e-Cigarette Advertising [Ph.D.]: Cornell University; 2022.
49. Okawa S, Tabuchi T, Miyashiro I. Who Uses E-cigarettes and Why? E-cigarette Use among Older Adolescents and Young Adults in Japan: JASTIS Study. 2020;52(1):37-45.
50. Cranwell J, Murray R, Lewis S, Leonardi-Bee J, Dockrell M, Britton J. Adolescents' exposure to tobacco and alcohol content in YouTube music videos. Addiction. 2015;110(4):703-11.
51. Williams RS, Derrick J, Ribisl KM. Electronic Cigarette Sales to Minors via the Internet. 2015;169(3):e1563-e63.
52. Schiff S, Liu F, Cruz TB, Unger JB, Cwalina S, Leventhal A, et al. E-cigarette and cigarette purchasing among young adults before and after implementation of California’s tobacco 21 policy. Tob Control. 2021;30(2):206.
53. Statista. Social media sites or apps used by children in the United Kingdom (UK) in 2020 2020 [Available from: https://www.statista.com/statistics/415138/social-media-usage-among-children-uk/#:~:text=Youth%20accounts%20on%20the%20rise,2020%2C%20peaking%20at%2087%20percent.
54. Statista. Reach of leading social networking sites used by those aged 15 to 25 in the United Kingdom (UK) as of 3rd quarter 2020 2022 [Available from: https://www.statista.com/statistics/1059462/social-media-usage-uk-age/.
55. Depue JB, Southwell BG, Betzner AE, Walsh BM. Encoded exposure to tobacco use in social media predicts subsequent smoking behavior. Am J Health Promot. 2015;29(4):259-61.
56. Hébert ET, Case KR, Kelder SH, Delk J, Perry CL, Harrell MB. Exposure and Engagement With Tobacco- and E-Cigarette-Related Social Media. J Adolesc Health. 2017;61(3):371-77.

For peer review only

BMJ Open

Youth's exposure to and engagement with e-cigarette marketing on social media: a UK focus group study

Journal:	*BMJ Open*
Manuscript ID	bmjopen-2022-071270.R1
Article Type:	Original research
Date Submitted by the Author:	05-Apr-2023
Complete List of Authors:	Smith, Marissa; University of Glasgow, MRC/CSO Social and Public Health Sciences Unit<br>Hilton , Shona; University of Glasgow, MRC/CSO Social and Public Health Sciences Unit
**Primary Subject Heading**:	Public health
Secondary Subject Heading:	Qualitative research
Keywords:	PUBLIC HEALTH, QUALITATIVE RESEARCH, Health policy < HEALTH SERVICES ADMINISTRATION & MANAGEMENT

SCHOLARONE™
Manuscripts

I, the Submitting Author has the right to grant and does grant on behalf of all authors of the Work (as defined in the below author licence), an exclusive licence and/or a non-exclusive licence for contributions from authors who are: i) UK Crown employees; ii) where BMJ has agreed a CC-BY licence shall apply, and/or iii) in accordance with the terms applicable for US Federal Government officers or employees acting as part of their official duties; on a worldwide, perpetual, irrevocable, royalty-free basis to BMJ Publishing Group Ltd ("BMJ") its licensees and where the relevant Journal is co-owned by BMJ to the co-owners of the Journal, to publish the Work in this journal and any other BMJ products and to exploit all rights, as set out in our licence.

The Submitting Author accepts and understands that any supply made under these terms is made by BMJ to the Submitting Author unless you are acting as an employee on behalf of your employer or a postgraduate student of an affiliated institution which is paying any applicable article publishing charge ("APC") for Open Access articles. Where the Submitting Author wishes to make the Work available on an Open Access basis (and intends to pay the relevant APC), the terms of reuse of such Open Access shall be governed by a Creative Commons licence – details of these licences and which Creative Commons licence will apply to this Work are set out in our licence referred to above.

Other than as permitted in any relevant BMJ Author's Self Archiving Policies, I confirm this Work has not been accepted for publication elsewhere, is not being considered for publication elsewhere and does not duplicate material already published. I confirm all authors consent to publication of this Work and authorise the granting of this licence.

For peer review only

Youth's exposure to and engagement with e-cigarette marketing on social media: a UK focus group study

Marissa J. Smith[1*] and Shona Hilton[1]

[1] MRC/CSO Social and Public Health Sciences Unit, University of Glasgow, Glasgow, G3 7HR, UK

*Corresponding author

Email: marissa.smith@glasgow.ac.uk Tel: (+44) 0141 353 7500

Keywords: Health policy, qualitative research, public health

For peer review only

ABSTRACT

Objective: E-cigarettes are promoted creatively through social media and considering the potential influence of social media marketing on young people, we explored young people's exposure to and engagement with social media marketing of e-cigarettes.

Design: Semi-structured discussion groups.

Subjects: Twenty focus groups with 82 young people aged 11-16 living in the Central belt of Scotland.

Methods: Youths were asked about smoking and vaping behaviours, social media use, vaping advertisement exposure, and were shown illustrative examples of social media content (e.g., images and videos) about different messages, presentations, and contextual features. Transcripts were imported into NVivo 12, coded thematically and analysed.

Results: Youths highlighted a variety of tactics e-cigarette companies use, including influencer or celebrity endorsement, attractive youth flavours, bright colours and emotional appeal to advertise and promote their products directly to young people. Social media influencers who advertise e-cigarettes were described as portraying e-cigarettes as 'cool' and 'fashionable' to entice viewers to try the products. Youths considered that there is a need for more restrictions on social media content to protect youths while also still allowing smokers to purchase them as a cessation device.

Conclusions: Our study highlights that the e-cigarette industry is using previously employed tactics similar to the tobacco industry to advertise and promote its products on social media. These findings suggest the growing need for governments to work together to develop and implement policies to restrict the advertising and marketing of e-cigarettes on social media.

For peer review only

Strengths and limitations of this study

- This study is one of the first in-depth studies to explore youths' responses to and engagement with e-cigarette marketing on social media with a focus on the rise in user-generated and influencer content.
- This research is very timely as governments consider the need for further e-cigarette regulations.
- A methodological strength is the rich dataset from semi-structured interviews with 82 youths aged 11-16, facilitating understandings of youths' exposure to and interaction with e-cigarette marketing on social media.
- Qualitative thematic analysis of the data allows depth of opinions but cannot offer predictions about the frequency of specific opinions with a wider population.

For peer review only

INTRODUCTION

Marketing strategies used by electronic cigarette (e-cigarette) companies have been associated with the uptick in e-cigarette use (vaping) among youth [1]. Many studies have found that the promotion of e-cigarettes through various channels (e.g., television (TV), social media influencers) has lent to increased positive perceptions of vaping and intentions to use vaping products and contributed to e-cigarette uptake among youth [2-4].

In the UK, the advertising of e-cigarettes is regulated through Article 20(5) of the revised European Union (EU) Tobacco Products Directive (2014/40/EU) (TPD), [5] which was transposed into UK law by the Tobacco and Related Products Regulations (TRPR) 2016 [6]. The TPD prohibited the advertising of nicotine-containing e-cigarettes (unless licensed as medicines) in channels with potential cross-border impact (i.e. channels that show adverts or sponsored events that originate from non-EU countries in EU countries), including TV, radio, newspapers, magazines, and sponsorship [7]. Online advertising was also prohibited under the TPD, although the regulations left scope for marketers to retain websites containing factual information about e-cigarette products. As there are currently no medicinally licensed nicotine vaping products in the UK, the prohibitions apply to all nicotine-containing e-cigarette products on the market. The TRPR requirements for e-cigarette advertising were set out in 2017 and are enforced by the Committee of Advertising Practice (CAP) – a self-regulatory body of organisations representing advertising, direct marketing, media businesses, and sales promotion endorsed and administered by the independent Advertising Standards Authority (ASA) [8, 9]. Although Rule 22.12 of the ASA CAP code prohibits advertising in online media, social media content for e-cigarettes is permitted in “non-paid-for space online under the marketer’s control, provided claims are factual rather than promotional” [10]. Promotional content includes descriptive language that goes beyond objective, factual claims, significant imagery that is not related to the product, and health claims (e.g. that e-cigarettes are safer or healthier than tobacco) [10].

Social media marketing includes promotions by means of paid digital advertising, via compensated ‘influencers’ (individuals with large followings who are paid to advertise brands and products), and on brand pages that companies can create for free and use to share content [11]. Most adolescents use

For peer review only

visual-based social media daily. User-generated and influencer marketing content on social media represents a key influence on young people's understanding of products, including e-cigarettes [12]. E-cigarettes are promoted creatively through social media, with well-designed features including colours, flavour variations, emotional appeal, incentives (such as price promotions and discount vouchers), and even celebrity endorsements [13]. Research has shown that online marketing that leads to exposure to e-cigarette advertising, including where it is concealed as information or recommendations from peers, can increase the likelihood of vaping in individuals, including among youths [14-16]. In addition, a number of researchers have suggested that e-cigarette advertising could lead to increases in how socially acceptable and desirable cigarettes are perceived to be, and subsequently influence their continued use or possible (re)uptake in smokers, e-cigarette users and dual-users [17-20]. E-cigarette companies have been promoting their products on social media in an attempt to expand their market [21, 22]. In addition, e-cigarette companies use social media platforms to promote e-cigarettes and create a socially attractive vaping image [23]. Previous studies [including 24, 25, 26] have explored youths' perceptions of e-cigarette marketing on social media, finding that social media adverts entice non-smokers to use e-cigarettes, emotional appeals are featured in adverts, use of vibrant colours in the packaging, appeal of flavours and advertisements portray e-cigarettes as less harmful than combustible cigarettes. Liu et al.[27] examined youths; perception of tobacco and marijuana messaging on social media and reported similar findings (including, appealing colours in the packaging and appealing flavours). One limitation of the literature in this area is that it focuses on either smokers, non-smokers and/or vapers. This leaves out non-vapers and dual-users (those who use both cigarettes and e-cigarettes), understudied. It is important to establish whether exposure to e-cigarette adverts on social media influence attitudes towards e-cigarettes in non-vapers and smoking in dual-users.

Gaining a better understanding of the nature, extent, and impact of e-cigarette marketing including their possible effects on youth e-cigarette perceptions and use can help guide the development of policies and interventions to educate youths about e-cigarettes [28]. This study, therefore, aims to explore youths', including non-smokers and non-vapers and dual-users exposure to, and engagement with e-cigarette advertising on social media, including user-generated and influencer content.

For peer review only

METHODS

We conducted 20 focus groups in Scotland between March and May 2022. Focus groups included between three and five participants (a total of 82 participants). Purposive sampling was used to recruit a diverse sample of youths in terms of age, sex, socio-economic background, smoking status, and vaping status [29]. Eleven groups were recruited through youth workers in local youth organisations. These gatekeepers handed out information sheets and helped achieve the sampling frame in terms of youth demographics and experiences concerning combustible cigarettes and e-cigarettes. The three organisations that helped with participant recruitment worked specifically with young people from disadvantaged backgrounds in urban areas. This recruitment strategy resulted in the inclusion of a range of participants from more and less deprived backgrounds and with experiences of smoking and vaping. Seven groups were recruited through the Schools Health and Wellbeing Improvement Research Network (SHINE) Newsletter which is distributed monthly to over 500 schools in Scotland. The remaining two groups were recruited via personal networks directly by MS.

Focus group discussions were facilitated to allow the research team to explore opinions on online advertising and marketing of e-cigarettes. Friendship groups of 3–5 participants were used to facilitate in-depth insights and promote participant interaction. Each participant was given a £20 shopping voucher as compensation for their time.

Prior to the start of the focus groups, participants completed a short anonymous questionnaire about their age, biological sex, postcode, smoking, and e-cigarette use status. For both combustible cigarettes and e-cigarettes, the questionnaire asked participants to specify whether they had tried or used them in the past or were using them at the time of the study. Based on a scoping review of user-generated content and influencer marketing involving e-cigarettes on social media [30], a topic guide was developed which covered three key areas, including use of social media, perceptions of social media influencers versus user-generated e-cigarette content on social media, and views on the marketing of e-cigarettes on social media.

For peer review only

For peer review only - http://bmjopen.bmj.com/site/about/guidelines.xhtml

Images of different types of e-cigarettes ('tanks', disposables, and pod devices) and e-liquids posted by social media influencers and general users were used as conversation starters found during the scoping review part of this research [30], were used as conversation starters. Group discussions were facilitated by MS (a young post PhD early career researcher post and experienced qualitive researcher). Ten of the groups were conducted online using Microsoft Teams and ten of the groups were conducted face-to-face. Of these, one of the groups was conducted on the youth organisation's premises, and the other nine were conducted at the school, with representatives of the youth organisation present. Groups lasted between 40 and 66 minutes. Field-notes reflecting on the focus group and individual issues discussed were written up for each group. All focus groups were audio recorded (with participants' permission) and transcribed verbatim.

Analysis

Following Braun and Clarke's six-phase framework for thematic analysis [31], we conducted thematic analysis of the data form the focus groups. The research team read and re-read the transcripts to become familiar with the data, and then iteratively constructed a coding frame to enable consistent organisation of relevant data. NVivo was used to organise categories on the basis of inductive themes that emerged from close reading of the, capture of both areas of agreement and less typical perspectives across a range of categories. Each transcript was imported into NVivo 12, coded independently, cross-checked, and analysed by MS and SH (professor of public health and experienced qualitive researcher). Contradictory cases and group dynamics were discussed, making use of transcripts and field notes. The researchers reflected on their role as researcher, remained constantly aware of their position and took care not to introduce bias throughout the research. To further reduce bias the researcher (MS) recorded the focus groups and analysed them some time after they were completed ensuring a more reflective viewpoint of occurrences. Ethical approval for the study was obtained from the University of Glasgow's Medical and Veterinary Life Sciences Ethics Committee (reference 200210034).

For peer review only

Patient and Public Involvement

Patients and/or the public were not involved in the design of the study, developed of the research questions, recruitment, outcome measures or conduct of the study. A summary of the results will be disseminated to study participants.

RESULTS

Participant characteristics

Eighty-two youths aged 11–16 years participated (47 females (57%) and 35 males (43%)), representing diversity in sociodemographic characteristics and smoking-related behaviours. Age distribution within the sample was skewed slightly towards 14–15-year-olds, with 14-year-olds making up the largest subgroup (n= 24). While the majority of participants did not currently smoke or use e-cigarettes, the sample included 10 smokers and 18 youths who used e-cigarettes. Deprivation rank was assigned using the Scottish Index of Multiple Deprivation (SIMD) [32]. The area-level deprivation grouped using a binary deprivation variable (least deprived/most deprived) in which the three most deprived quintiles were grouped into the most deprived category for the Central Belt of Scotland area. Table 1 describes the focus group composition and participants in more detail and Table 2 summarises smoking and e-cigarette use among the sample.

For peer review only

For peer review only - http://bmjopen.bmj.com/site/about/guidelines.xhtml

Group	Area-level deprivation	Sex	Age	Cigarette Smoker	E-cigarette use
1	Least deprived	Female	13-15	Never	Never
2	Least deprived	Female	14-15	Never	Mixed – Never (4) / Tried (1)
3	Least deprived	Female	13-16	Never	Mixed – Never (2) / Tried (1)
4	Most deprived	Mixed – Male (3)/Female (2)	12-15	Mixed – Never (3) / Current (2)	Mixed – Never (3) / Tried (1)/ Current (1)
5	Most deprived	Mixed – Male (1)/ Female (4)	14-16	Mixed – Never (2)/ Tried (2)/ Current (1)	Mixed – Never (2)/ Tried (2)/ Current (1)
6	Most deprived	Male	12-15	Never	Never
7	Most deprived	Male	16	Current	Current
8	Least deprived	Mixed – Male (2)/Female (3)	14	Never	Never
9	Most deprived	Male	16	Mixed – Tried (1)/ Current (2)	Current
10	Most deprived	Mixed – Male (4)/Female (1)	14-15	Mixed – Never (3)/ Tried (1)/ Current (1)	Mixed – Never (3)/ Tried (1)/ Current (1)
11	Most deprived	Mixed – Male (3)/Female (2)	13-16	Mixed – Never (2)/ Tried (2)/ Current (1)	Mixed – Never (1)/ Current (4)
12	Least deprived	Mixed – Male (2)/Female (1)	15-16	Tried	Mixed – Tried (2)/ Current (1)
13	Least deprived	Female	13-16	Never	Never
14	Most deprived	Mixed – Male (1)/Female (3)	11-12	Never	Never
15	Most deprived	Mixed – Male (3)/Female (1)	11-12	Never	Never
16	Most deprived	Mixed – Male (2)/Female (2)	11-12	Never	Never
17	Most deprived	Female	14-16	Mixed – Never (4)/ Tried (1)	Mixed – Never (1)/ Tried (1)/ Current (3)
18	Most deprived	Male	13-16	Never	Never
19	Most deprived	Female	14	Never	Mixed – Tried (2)/ Current (1)
20	Least deprived	Female	15-16	Never	Tried (3)

174 **Table 1: Focus group location, participants, and their cigarette smoking and e-cigarette use**.

For peer review only - http://bmjopen.bmj.com/site/about/guidelines.xhtml

	E-cigarette use											
	Never			Tried			Current			Total		
**Cigarette smoker**	**n**	**(col %)**	**(row %)**	**n**	**(col %)**	**(row %)**	**n**	**(col %)**	**(row %)**	**n**	**(col %)**	**(row %)**
**Never**	51	98.1%	82.3%	7	58.3%	11.3%	4	22.2%	6.5%	62	75.6%	100.0%
**Tried**	1	1.9%	10.0%	4	33.3%	40.0%	5	27.8%	50.0%	10	12.2%	100.0%
**Current**	0	0.0%	0.0%	1	8.3%	10.0%	9	50.0%	90.0%	10	12.2%	100.0%
**Total**	52	1	92.3%	12	100.0%	14.6%	18	100.0%	22.0%	82	100.0%	100.0%

Table 2: E-cigarette use according to cigarette smoking.

For peer review only

Perceptions of advertising tactics used by the e-cigarette industry to target youths

Youths discussed three ways (influencer or celebrity endorsement, flavourings and emotional appeal) e-cigarette companies market their products to target young people.

Influencer or celebrity endorsements

During the focus groups, participants frequently discussed the prevalence of social media influencers advertising e-cigarette products. When discussing why e-cigarette and/or tobacco companies would want to use social media influencers to advertise their products, participants highlighted the popularity of social media and how this would increase the reach of the adverts.

> *"I guess companies would use them [influencers] because the company may have approached them to advertise the product and they know they might have a huge following on the platform, so they might influence some more people to buy the products." (Male, current smoker, current vaper)*

> *"More people are on social media nowadays. So, it's better that they [vaping/tobacco companies] use influencers to advertise their products compared to say in newspapers, 'cause you don't see people reading newspapers anymore." (Male, never smoker, never vaper)*

Participants reported seeing marketing and advertising of e-cigarettes on social media and that they were portrayed as 'cool' and 'fashionable' by social media influencers.

> *"If an influencer posts it, a lot of people see it and be like, that's the trend now, it's a cool thing to do, so I want to take part in that." (Female, never smoker, tried vaping)*

For peer review only

> *"It's like they're showcasing them. It's not just like it's in the background or whatever, it's not a normal pose to have them in, it's like they're showcasing them and that they are a fashionable thing to carry or to have." (Female, never smoker, never vaper)*

Participants discussed that this would influence the viewer to try the product, thus increasing the prevalence of use.

> *"People get tempted easily by seeing the post. Like if they saw some influencers trying that stuff [e-cigarettes], people get tempted quickly to try that stuff, because then that person is trying it so the like the kids would think this is something that I should also try that stuff like that, that's the point." (Male, current smoker, current vaper)*

> *"When you see what an influencer is, most people think oh if they're doing that, then it's cool, so it like, it's making more people want to buy the vapes, and stuff." (Female, never smoker, tried vaping)*

Several participants discussed how social media posts make it too easy for underage youths to purchase and hide the fact you are buying e-cigarette products from websites.

> *"I've seen a thing on TikTok like, they [influencers] show you them putting them [e-cigarettes] in the wee boxes and all that, or you could put them in secret packaging like behind the lashes. Like you can order it off their website and they'll hide it in the packaging, they put a few bits of sweeties on top of your vapes so your mum doesn't see it." (Female, tried smoking, current vaper)*

Using sweet and fruity flavours to appeal to youths

For peer review only

Participants discussed the variety of flavours of e-cigarettes available, highlighting that the sweet and fruity flavours are particularly attractive to youths.

> *"'Cause younger people, like us would think that the flavoured ones look nice so they would try then and then they'd start vaping." (Male, never smoker, never vaper)*

> *"I think the type of ice cream flavours or sweetie ones are targeted at younger people because most people in their 30s and 40s would probably use the tobacco one or the coffee ones." (Male, current smoker, current vaper)*

> *"The ones that stood out to me the most were the Ben and Jerry's and fruit ones compared to the tobacco ones. I feel like, if I was going to buy them, I would buy them 'cause they are attractive and are things I like." (Female, tried smoking, current vaper)*

Several participants stated that the variety of flavourings was to appeal to youths.

> *"I feel like no one really wants to taste tobacco, to be honest. But I think putting out there with like grape and blueberry flavourings are going to attract younger people to the marketplace. I think it's an intentional design and I feel like that is probably what it does attract youths." (Female, never-smoker, never vaper)*

Using vibrant colours to attract youth attention

In addition, participants spoke about the presentation of e-cigarette products on social media, for example, the use of vibrant colours and filters.

For peer review only - http://bmjopen.bmj.com/site/about/guidelines.xhtml

"You can tell by the picture they're promoting them. Because if it was not promoting them you would have like a red colour, it's like a stop, it's kind of like a no colour. But they are using really positive colours, like pink and green. It makes you stop and look at it to see what it's [the social media post] is about." (Male, never smoker, never vaper)

"It was really bright colours and they had bright filters on them so it is brighter. If you were scrolling past then gives you a bright colour, you'd go back and see it. You'd be like, oh what's that? You'd be, like, drawn to it. […] I think young people like us would be drawn to that, well I would." (Female, never smoker, never vaper)

Connecting emotionally with youths

Participants discussed the emotional connections and positive portrayal of e-cigarettes on social media, stating that individuals in the social media posts 'looked happy' to be using the products.

"I think the person who posted this product, I think he or she was trying to emphasise the fact that these products are good because they look happy to be advertising it. So, you should try them and like they were not promoting vaping negatively or that it is bad at all. I think they were trying to sell the aesthetic idea of this product." (Male, current smoker, current vaper)

"It's the way they're all holding them, they looked quite passionate with a smile on their face. A big cheesy smile." (Male, current smoker, current vaper)

For peer review only - http://bmjopen.bmj.com/site/about/guidelines.xhtml

Participants highlighted the use of emojis in e-cigarette social media posts. Emojis (icons used to express an idea or emotion) and emoji sequencing (the use of two or more emojis that form a conceptual rhetorical unit) [33] are used to enhance the message meaning [34, 35]. When asked if this would affect their interaction with social media posts that included emojis, participants stated that they would be more inclined to read the post, rather than scrolling past.

"If you see it in colours, like emojis, you're like, oh I wonder what that is. You'd stop scrolling. 'Cause normally captions is just, like, text and a black background or something like that." (Female, never smoker, never vaper)

"The wee emojis they would draw my attention, because of how brightly coloured they are. And I think that's what they're trying to do, like, it'll draw your attention." (Female, never smoker, never vaper)

Perceptions of what can be done to limit e-cigarette marketing on social media

Participants had divergent views on the marketing and advertising of e-cigarettes on social media. Several participants stated that marketing and advertising should not be allowed as youths, including those of a similar age, could access this type of content and it may encourage them to try e-cigarettes.

"Advertising them [e-cigarettes] on social media is s a bad thing because younger people might see it and then try it." (Male, never smoker, never vaper)

I don't think it [advertising of e-cigarettes] should be allowed, just because of the younger community on it [social media]. And then if they are seeing them, they might end up doing it, getting addicted and end up dying." (Male, tried smoking, tried vaping)

For peer review only - http://bmjopen.bmj.com/site/about/guidelines.xhtml

But participants also acknowledged that controlling what is posted on social media is ‘tricky’ and ‘difficult’ and that prohibiting e-cigarette advertising may limit their role as cessation devices by smokers. Those participants suggested instead of prohibiting the marketing and advertising of e-cigarettes, warnings (such as age and health warnings) or other restrictive measures (such as parental controls or age restrictions) could be imposed on these types of social media posts.

“I think there needs to be age restrictions on the posts or parents could control what their child sees.” (Female, never smoker, never vaper)

DISCUSSION

E-cigarettes have become increasingly popular and visible in public life, particularly on social media. Our qualitative thematic analysis of youths’ perceptions of and engagement with e-cigarette advertising on social media highlighted a public health concern. Similar to previous research [36-38], youths in our study highlighted the tactics used by the e-cigarette industry to promote and advertise e-cigarettes to underage populations through influencer and celebrity endorsements, variety of flavours and using promotional endorsements. Youths discussed the positive portrayal of e-cigarettes by social media influencers, and they were enticing the viewer to buy the product. Youths highlighted that e-cigarette and/or tobacco companies use social media influencers to advertise their products on social media and by adverting on social media platforms, they will increase the reach of the adverts to a wider audience. We found that youths had divergent views on e-cigarette marketing on social media. Several participants stated that e-cigarette advertising on social media should be prohibited, citing the high prevalence of youths on social media who could access the content. While others suggested a balanced approach to regulating advertising on social media so that they can still be advertised as cessation devices by smokers. Youths suggested that all adverts should contain warnings (such as age and health warnings) or other restrictive measures (such as parental controls or age restrictions) to protect youths and non-users.

For peer review only

Previous studies which examined e-cigarette-related social media [24-26, 39-43] found that the vast majority of the content depicted positive attitudes towards vaping, while negative characterisations were mostly absent. Our study found that youths had similar experiences when exposed to e-cigarette content on social media. Consequently, there is a risk that youths will be exposed to, and possibly engage with, content that promotes vaping while staying uninformed about the negative aspects, including potential health harms. Our study adds to existing concerns about youths' perceptions of e-cigarettes as cool, fashionable products [44-47], by considering the advertising of the products by influencers on social media platforms, frequently accessed by youths. The use of social media influencers to promote products and celebrity endorsements encourage their followers to share and interact with their content, and ultimately purchase their products. Social media influencers often collaborate with multiple industries (e.g., fashion, and beauty products) in addition to e-cigarette products/brands. These influencers could potentially expose their non-e-cigarette-focused audience (including non-users of e-cigarettes) to e-cigarette content [48]. Therefore, these influencers could be considered an even higher risk for youth compared to influencers who post exclusively about e-cigarettes. This is problematic and concerning as most e-cigarette brands claim that their advertising and promotional content is meant to target current cigarette smokers to help them switch to e-cigarettes, [49, 50] rather than people who do not use nicotine at all. Previous literature has focused on either smokers, non-smokers and/or vapers, leaving out non-vapers and dual-users. Participants in our study were primarily non-smokers and non-vapers, we argue that it is important to explore non-smokers and non-vapers exposure to and engagement with e-cigarette adverts on social media. This is important for helping to determine the potential influence of e-cigarette adverts on social media in non-smokers and non-vapers.

Before plain packaging, the tobacco industry successfully used colourful packaging to represent its brand and increase the appeal of smoking [51-53]. The marketing tactics used by the tobacco industry were so effective that several e-cigarette manufacturers have used the same trade secrets to advertise their products [54-56]. Research has shown the plethora of bright-coloured packaging, eye-catching designs, and variety of flavourings available all appeal to youths and may result in experimentation of the products [57-62].

For peer review only

Previous research has illustrated YouTube viewers' exposure to e-cigarettes (as well as alcohol and tobacco) while watching videos on the platform [63]. Our study adds to this by illustrating the potential mechanisms by which exposure to e-cigarette advertising on social media platforms may influence perceptions and resultant trial/use of the products. While restrictions on mass media marketing of e-cigarettes are increasingly considered internationally, social media platforms are more difficult to manage because user-generated content (in particular) will not be covered by incoming regulations. Our study findings resonate with previous research, which found that youths were easily able to purchase e-cigarettes from the internet due to the absence of age-verification measures used by internet vendors [64, 65].

As with all research, our study has some limitations. First, and consistent with the qualitative design, the sample does not aim to be representative of UK youth, as our study focused on Scottish youths. However, we did have a diverse sample of both sexes. Secondly, the study's geographical remit has to be considered when interpreting the findings. The UK is considered an international leader in tobacco control policy. It is possible that participants' views may have been influenced by the UK's unique favourable policy approach to e-cigarettes and legal and socio-cultural context, including low smoking prevalence. Thirdly, using friendship groups may have influenced youth’s honesty when answering questions and their responses. Youth’s may not have wanted to disagree with their peers, thus responded in a similar way to the rest of the group. Fourthly, participants in this study were primarily non-smokers and non-vapers and it is possible this may have impacted in their discussion of e-cigarette advertisements on social media. However, as we used predominately visual prompts based upon data found during the scoping review of the research [30] we did not find that non-smokers and non-vapers contributed less to discussions. If we had not used visual prompts and focused on participants own experiences of what they had seen on social media (i.e., recalling from memory) this may have impacted non-smokers and non-vapers contribution to discussions. Fifthly, the data was collected in different formats (online and face-to-face), and this may have influenced participants’ responses. Two of the online groups were conducted in a classroom with a teacher present, and during seven face-to-face groups, a teacher/youth worker was present in the room. The presence of a

For peer review only

teacher/youth worker may have influenced youth's willingness to answer questions and their responses. Finally, two of the groups were recruited through personal networks and this may have impacted the youth's responses.

The results from this study should provide guidance for future research. More research is needed to determine the most effective means to counter the favourable/positive aspects of e-cigarettes to reduce youths' interest in product trial and use. Future research could explore if there are different perceptions of e-cigarette exposure in social media between females and males. As females are one of the target groups for e-cigarette use [66, 67], it can reflect the marketing situation of e-cigarettes for females on social media, which deserves further study. In addition, research on the prevalence of warning statements on e-cigarette-related social media posts and the impact this has on youth perceptions would be an important future direction to expand this work.

Considering that most youth access multiple social media platforms multiple times per day [68, 69] and that exposure to this marketing is related to use [70, 71], our findings indicate a significant public health concern. Given the findings presented by our study, there is a growing need for policymakers to develop comprehensive plans to build more robust measures to protect youths and to restrict the ability of marketers to reach youths with enticing social media content promoting e-cigarettes. In addition, social media platforms should consider implementing more robust measures, such as age restrictions and portraying the negative aspects of vaping, to ensure the prevention of vaping-related content targeted at underage users.

CONCLUSION

The tactics used on social media platforms for advertising and marketing e-cigarettes directly to youths raises concerns and a new generation of youths becoming addicted to nicotine. The results of this study highlight that the e-cigarette industry is using previously employed tactics similar to the tobacco industry to advertise and promote its products on social media. Most youths use social media

For peer review only - http://bmjopen.bmj.com/site/about/guidelines.xhtml

daily and the depiction of products on social media represents a key influence on young people's understanding of products. These findings may highlight a priority for governmental policy to restrict the ability of marketers to reach youths with social media content promoting e-cigarettes.

Declarations

Authors' contributions: **Marissa J. Smith:** Conceptualisation, Data Curation, Investigation, Methodology, Validation, Visualisation**,** Writing- Original draft preparation. **Shona Hilton:** Conceptualisation, Methodology, Validation, Writing - Review & Editing.

Ethical approval: Ethical approval for the study was obtained from the University of Glasgow's Medical and Veterinary Life Sciences Ethics Committee (reference 200210034).

Competing interests: The authors declare that they have no competing interests.

Data sharing statement: All data relevant to the study are included in the article or uploaded as supplementary information**.**

Funding: MS acknowledges funding from Cancer Research UK grant PPRCTAGPJT\100003. SH is funded by the Medical Research Council grant MC_UU_00022/1, the Chief Scientist Office of the Scottish Government Health Directorates grant SPHSU17, and Cancer Research UK grant PPRCTAGPJT\100003.

Role of the funding source: The funders of this study had no role in the study design, data analysis, data interpretation, or writing of the report. The corresponding author had full access to all of the data and final responsibility for the decision to submit it for publication.

Acknowledgements: Patients and or public were not involved.

REFERENCES

1. Hammond D, Wackowski OA, Reid JL, O'Connor RJ. Use of JUUL E-cigarettes Among Youth in the United States. Nicotine Tob Res. 2020;22(5):827-32.
2. Duke JC, Allen JA, Eggers ME, Nonnemaker J, Farrelly MC. Exploring Differences in Youth Perceptions of the Effectiveness of Electronic Cigarette Television Advertisements. Nicotine Tob Res. 2016;18(5):1382-86.

For peer review only

3. Farrelly MC, Duke JC, Crankshaw EC, Eggers ME, Lee YO, Nonnemaker JM, et al. A Randomized Trial of the Effect of E-cigarette TV Advertisements on Intentions to Use E-cigarettes. Am J Prev Med. 2015;49(5):686-93.
4. Hammond D, Reid JL, Burkhalter R, Rynard VL. E-cigarette Marketing Regulations and Youth Vaping: Cross-Sectional Surveys, 2017–2019. 2020;146(1).
5. European Commission. Tobacco Products Directive (2014/40/EU). European Commission; 2014. p. 1-38.
6. The Tobacco and Related Products Regulations 2016, 507. Sect. Part 2 (2016).
7. Stead M, Ford A, Angus K, MacKintosh AM, Purves R, Mitchell D. E-Cigarette Advertising in the UK: A Content Analysis of Traditional and Social Media Advertising to Observe Compliance with Current Regulations. Nicotine Tob Res. 2021;23(11):1839-47.
8. Advertising Standards Authority. About the ASA and CAP 2021 [Available from: https://www.asa.org.uk/about-asa-and-cap/about-regulation/about-the-asa-and-cap.html.
9. Advertising Standards Authority. The CAP Code: The UK Code of Non-broadcast Advertising and Direct & Promotional Marketing. London, UK: Advertising Standards Authority; 2016.
10. Advertising Standards Authority. Electronic Cigarettes: Factual vs. Promotional Claims 2018 [Available from: https://www.asa.org.uk/advice-online/electronic-cigarettes-factual-vs-promotional-claims.html.
11. O'Brien EK, Hoffman L, Navarro MA, Ganz O. Social media use by leading US e-cigarette, cigarette, smokeless tobacco, cigar and hookah brands. Tob Control. 2020;29(e1):e87-e97.
12. De Veirman M, Hudders L, Nelson MR. What Is Influencer Marketing and How Does It Target Children? A Review and Direction for Future Research. Front Psychol. 2019;10.
13. Zhu S, Sun J, Binnevie E, Cummins S, Gamst A, Yin L, et al. Four hundred and sixty brands of e-cigarettes and counting: implications for product regulation. Tob Control. 2014;23(3):3-9.
14. Chu KH, Colditz JB, Primack BA, Shensa A, Allem JP, Miller E, et al. JUUL: Spreading Online and Offline. J Adolesc Health. 2018;63(5):582-86.
15. Phua J, Jin SV, Hahm JM. Celebrity-endorsed e-cigarette brand Instagram advertisements: Effects on young adults' attitudes towards e-cigarettes and smoking intentions. J Health Psychol. 2017;23(4):550-60.
16. Amin S, Dunn AG, Laranjo L. Social Influence in the Uptake and Use of Electronic Cigarettes: A Systematic Review. Am J Prev Med. 2020;58(1):129-41.
17. de Andrade M, Hastings G, Angus K. Promotion of electronic cigarettes: tobacco marketing reinvented? BMJ. 2013;DOI: 10.1136/bmj.f7473.
18. Fairchild AL, Bayer R, Colgrove J. The Renormalization of Smoking? E-Cigarettes and the Tobacco "Endgame". NEJM. 2013;370(4):293-95.
19. Schneider S, Diehl K. Vaping as a Catalyst for Smoking? An Initial Model on the Initiation of Electronic Cigarette Use and the Transition to Tobacco Smoking Among Adolescents. Nicotine Tob Res. 2016;18(5):647-53.
20. Etter J-F. Gateway effects and electronic cigarettes. Addiction. 2017;113(10):1776-83.
21. Cobb NK, Brookover J, Cobb CO. Forensic analysis of online marketing for electronic nicotine delivery systems. Tob Control. 2015;24(2):128.
22. Amin S, Dunn AG, Laranjo L. Exposure to e-cigarette information and advertising in social media and e-cigarette use in Australia: A mixed methods study. 2020;213:108112.
23. Prochaska JJ, Pechmann C, Kim R, Leonhardt JM. Twitter=quitter? An analysis of Twitter quit smoking social networks. Tob Control. 2012;21(4):447-49.
24. Chen Y, Tilden C, Vernberg DK. Adolescents' interpretations of e-cigarette advertising and their engagement with e-cigarette information: results from five focus groups. Psychol Health. 2020;35(2):163-76.
25. Scheffels J, Tokle R, Linnansaari A, Rasmussen SKB, Pisinger C. E-cigarette use in global digital youth culture. A qualitative study of the social practices and meaning of vaping among 15-20-year-olds in Denmark, Finland, and Norway. 2023;111:103928.
26. Johnson AC, Mays D, Hawkins KB, Denzel M, Tercyak KP. A Qualitative Study of Adolescent Perceptions of Electronic Cigarettes and Their Marketing: Implications for Prevention and Policy. Child Health Care. 2017;46(4):379-92.

For peer review only

27. Liu J, McLaughlin S, Lazaro A, Halpern-Felsher B. What Does It Meme? A Qualitative Analysis of Adolescents' Perceptions of Tobacco and Marijuana Messaging. Public Health Rep. 2020;135(5):578-86.

28. Pepper JK, McRee AL, Gilkey MB. Healthcare providers' beliefs and attitudes about electronic cigarettes and preventive counseling for adolescent patients. 2014;54(6):678-83.

29. Guest G, Bunce A, Johnson L. How Many Interviews Are Enough?:An Experiment with Data Saturation and Variability. 2006;18(1):59-82.

30. Smith MJ, Buckton C, Patterson C, Hilton S. User-generated content and influencer marketing involving e-cigarettes on social media: a scoping review and content analysis of YouTube and Instagram. BMC Public Health. 2023;23(1):530.

31. Braun V, Clarke V. Thematic analysis. APA handbook of research methods in psychology, Vol 2: Research designs: Quantitative, qualitative, neuropsychological, and biological. APA handbooks in psychology®.DOI: 10.1037/13620-004. Washington, D.C., USA: American Psychological Association; 2012. p. 57-71.

32. Scottish Government. The Scottish Index of Multiple Deprivation 2020 2020 [Available from: https://www.gov.scot/collections/scottish-index-of-multiple-deprivation-2020/.

33. Ge J. Emoji Sequence Use in Enacting Personal Identity. In: Liu L, White R, editors. WWW '19: Companion Proceedings of The 2019 World Wide Web Conference; New York, USA2019. p. 426-38.

34. Ge J, Gretzel U. Emoji rhetoric: a social media influencer perspective. 2018;34(15-16):1272-95.

35. Jacobson J, Hodson J, Mittelman R. Pup-ularity contest: The advertising practices of popular animal influencers on Instagram. 2022;174:121226.

36. Kostygina G, Tran H, Czaplicki L, Perks SN, Vallone D, Emery SL, et al. Developing a theoretical marketing framework to analyse JUUL and compatible e-cigarette product promotion on Instagram. Tob Control. 2022;10.1136/tobaccocontrol-2021-057120:tobaccocontrol-2021-057120.

37. Alpert JM, Jaisle A, Chen H. A content analysis of the promotional strategies employed by e-cigarette brands on Twitter. 2019;36(4):307-21.

38. Struik LL, Dow-Fleisner S, Belliveau M, Thompson D, Janke R. Tactics for Drawing Youth to Vaping: Content Analysis of Electronic Cigarette Advertisements. 2020;22(8):e18943.

39. Malik A, Li Y, Karbasian H, Hamari J, Johri A. Live, Love, Juul: User and Content Analysis of Twitter Posts about Juul. Am J Health Behav. 2019;43:326-36.

40. Allem JP, Dharmapuri L, Unger JB, Cruz TB. Characterizing JUUL-related posts on Twitter. Drug Alcohol Depend. 2018;190:1-5.

41. Cole-Lewis H, Pugatch J, Sanders A, Varghese A, Posada S, Yun C, et al. Social Listening: A Content Analysis of E-Cigarette Discussions on Twitter. J Med Internet Res. 2015;17(10):e243.

42. Ketonen V, Malik A. Characterizing vaping posts on Instagram by using unsupervised machine learning. Int J Med Inform. 2020;10.1016/j.ijmedinf.2020.104223.

43. Smith MJ, Buckton C, Patterson C, Hilton S. A scoping review and content analysis of YouTube and Instagram posts. In press.

44. de Andrade M, Angus K, Hastings G. Teenage perceptions of electronic cigarettes in Scottish tobacco-education school interventions: co-production and innovative engagement through a pop-up radio project. 2016;136(5):288-93.

45. Choi K, Fabian L, Mottey N, Corbett A, Forster J. Young adults' favorable perceptions of snus, dissolvable tobacco products, and electronic cigarettes: findings from a focus group study. Am J Public Health. 2012;102(11):2088-93.

46. Park E, Kwon M, Gaughan MR, Livingston JA, Chang Y-P. Listening to Adolescents: Their Perceptions and Information Sources About E-cigarettes. 2019;48:82-91.

47. Vogel EA, Ramo DE, Rubinstein ML, Delucchi KL, Darrow SM, Costello C, et al. Effects of Social Media on Adolescents' Willingness and Intention to Use E-Cigarettes: An Experimental Investigation. Nicotine Tob Res. 2020;10.1093/ntr/ntaa003.

48. Vassey J, Valente T, Barker J, Stanton C, Li D, Laestadius L, et al. E-cigarette brands and social media influencers on Instagram: a social network analysis. Tob Control. 2022;10.1136/tobaccocontrol-2021-057053:tobaccocontrol-2021-057053.

49. Dewhirst T. Co-optation of harm reduction by Big Tobacco. Tob Control. 2021;30(e1):e1.

For peer review only

50. Prakash S, Xu Y, Goldenson NI, Wissmann R, Gougelet R, Shiffman S. Transitions in Smoking among Adults Newly Purchasing the JUUL System. Am J Health Behav. 2021;45(3):546-62.
51. Wayne GF, Connolly GN. How cigarette design can affect youth initiation into smoking: Camel cigarettes 1983-93. Tob Control. 2002;11 Suppl 1(Suppl 1):I32-9.
52. Carpenter CM, Wayne GF, Connolly GN. Designing cigarettes for women: new findings from the tobacco industry documents. Addiction. 2005;100(6):837-51.
53. Anderson SJ, Glantz SA, Ling PM. Emotions for sale: cigarette advertising and women's psychosocial needs. Tob Control. 2005;14(2):127-35.
54. Campaign for Tobacco-Free Kids. Campaign for Tobacco-Free Kids. 2013. [cited 2022]. Available from: https://www.tobaccofreekids.org/blog/2013_10_02_ecigarettes.
55. Haugh M. University of Richmond. 2020. [cited 2022]. Available from: https://jolt.richmond.edu/2020/11/27/e-cigarettes-new-technology-but-old-advertising-strategy/.
56. Walley SC, Wilson KM, Winickoff JP, Groner J. A Public Health Crisis: Electronic Cigarettes, Vape, and JUUL. 2019;143(6).
57. Kong G, Morean ME, Cavallo DA, Camenga DR, Krishnan-Sarin S. Reasons for Electronic Cigarette Experimentation and Discontinuation Among Adolescents and Young Adults. Nicotine Tob Res. 2015;17(7):847-54.
58. Audrain-McGovern J, Strasser AA, Wileyto EP. The impact of flavoring on the rewarding and reinforcing value of e-cigarettes with nicotine among young adult smokers. Drug Alcohol Depend. 2016;166(1):263-67.
59. Czoli CD, Goniewicz M, Islam T, Kotnowski K, Hammond D. Consumer preferences for electronic cigarettes: results from a discrete choice experiment. Tob Control. 2016;25(1):30-36.
60. Zare S, Nemati M, Zheng Y. A systematic review of consumer preference for e-cigarette attributes: Flavor, nicotine strength, and type. 2018;13(3):e0194145.
61. Kalaji MNH. The Visual Is Worth Thousands of Words: Adult and Youth Perceptions of Visual Cues in e-Cigarette Advertising [Ph.D.]: Cornell University; 2022.
62. Okawa S, Tabuchi T, Miyashiro I. Who Uses E-cigarettes and Why? E-cigarette Use among Older Adolescents and Young Adults in Japan: JASTIS Study. 2020;52(1):37-45.
63. Cranwell J, Murray R, Lewis S, Leonardi-Bee J, Dockrell M, Britton J. Adolescents' exposure to tobacco and alcohol content in YouTube music videos. Addiction. 2015;110(4):703-11.
64. Williams RS, Derrick J, Ribisl KM. Electronic Cigarette Sales to Minors via the Internet. 2015;169(3):e1563-e63.
65. Schiff S, Liu F, Cruz TB, Unger JB, Cwalina S, Leventhal A, et al. E-cigarette and cigarette purchasing among young adults before and after implementation of California's tobacco 21 policy. Tob Control. 2021;30(2):206.
66. Pike JR, Tan N, Miller S, Cappelli C, Xie B, Stacy AW. The Effect of E-cigarette Commercials on Youth Smoking: A Prospective Study. 2019;43(6):1103-18.
67. Truth Initiative. Old tactics, new products: how big tobacco targets women in e-cigarette marketing 2023 [Available from: https://truthinitiative.org/research-resources/targeted-communities/old-tactics-new-products-how-big-tobacco-targets-women.
68. Statista. Social media sites or apps used by children in the United Kingdom (UK) in 2020 2020 [Available from: https://www.statista.com/statistics/415138/social-media-usage-among-children-uk/#:~:text=Youth%20accounts%20on%20the%20rise,2020%2C%20peaking%20at%2087%20percent.
69. Statista. Reach of leading social networking sites used by those aged 15 to 25 in the United Kingdom (UK) as of 3rd quarter 2020 2022 [Available from: https://www.statista.com/statistics/1059462/social-media-usage-uk-age/.
70. Depue JB, Southwell BG, Betzner AE, Walsh BM. Encoded exposure to tobacco use in social media predicts subsequent smoking behavior. Am J Health Promot. 2015;29(4):259-61.
71. Hébert ET, Case KR, Kelder SH, Delk J, Perry CL, Harrell MB. Exposure and Engagement With Tobacco- and E-Cigarette-Related Social Media. J Adolesc Health. 2017;61(3):371-77.

Appendix A: Interview topic guide

Part 1: Introduction/background

- First of all, thank you for taking part in this research.
- I'm [researcher name], a research assistant at the University of Glasgow with an interest in tobacco and e-cigarettes.

Key points for consent:

- Can I confirm that you have received the participant information sheet and signed and returned your consent form.
- Just to reiterate, your taking part is voluntary, and you are free to withdraw at any time without giving a reason.
- Your participation will be anonymous. Excerpts from the interview may be quoted verbatim in a report and a paper, but quotations will be anonymised.
- Non-anonymised interview recordings and transcripts will be destroyed securely upon completion of this research, but anonymised transcripts and consent forms will be stored securely by the University of Glasgow for a period of 10 years for the purposes of ensuring research integrity.
- Finally, the study has been approved by the Ethics Committee of the College of Social Sciences at the University of Glasgow.
- Reiterate that I am interested to hear their thoughts and experiences as things on social media are changing so quickly and they will know more than me.

Young people's use of social media

First of all, I am going to ask you about social media and what you use.

Show Slide 1 of PowerPoint presentation.

1. Do you recognise any of these logos?
    a. PROMPT: Can you tell me what each logo is?

For peer review only

   b. PROMPT: What do you know about each one?

2. Can you tell me about the social media platforms that you use?
   a. Why do you use those?
   b. In what way do you use those?
      i. PROMPT: How do you use them? E.g., ‘liking’, commenting, sharing?
      ii. PROMPT: What is the purpose of each?
      iii. PROMPT: Whether all are the same in the sense of what you can do on them or whether some are different?
   c. What social media platforms do you like and dislike?
      i. PROMPT: Why?
   d. On a daily basis, how long do you tend to spend on social media?
3. What do you think the term ‘social media influencer’ means?
   a. What do social influencers do?
   b. How do you know that someone is a social influencer?
   c. Can you tell me of any social media influencers who you like and dislike?
   d. What products have you seen influencers advertising?
      i. PROMPT: Clothing, makeup?
   e. How do you think they pick the products that they advertise?
   f. Have you ever bought or used products based on what you have seen on social media?
      i. PROMPT: What type of products were those?
   g. Would you ever like, comment on or share a influencers image if they were advertising something?

<u>**E-cigarettes on social media**</u>

Now I am going show a few slides on PowerPoint and after we have been through them, we will chat through what you have seen.

Show Slides 2-8 of PowerPoint presentation.

For peer review only

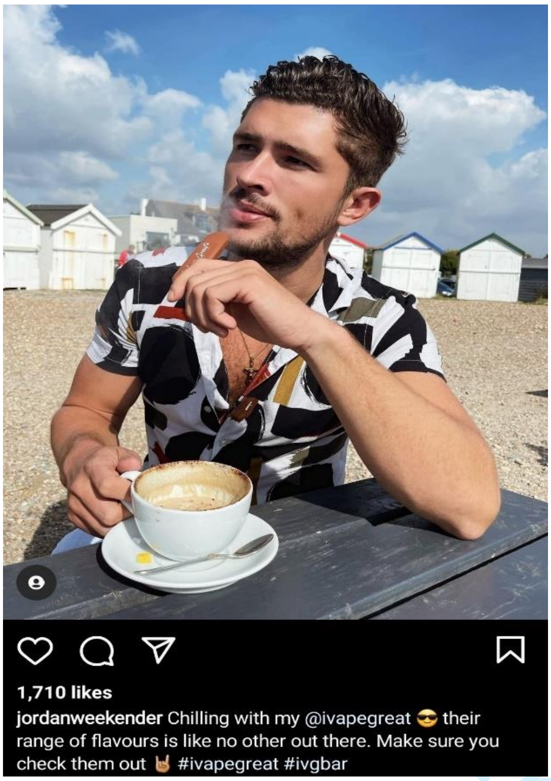

Slide 2

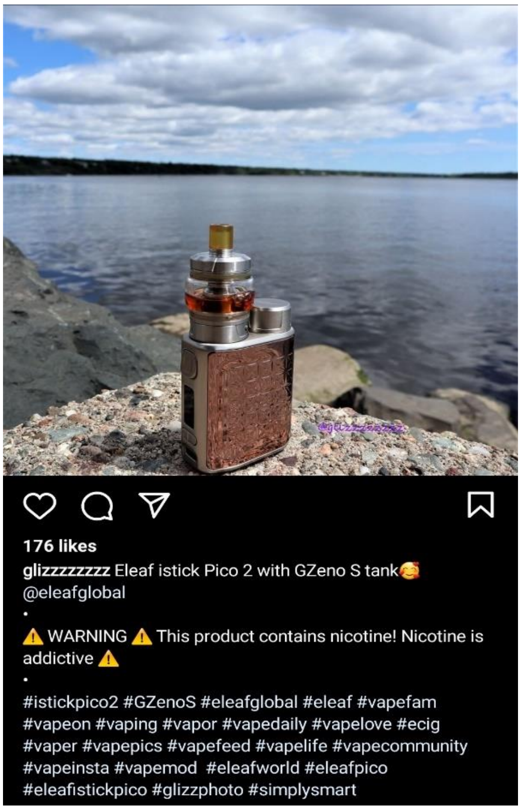

Slide 3

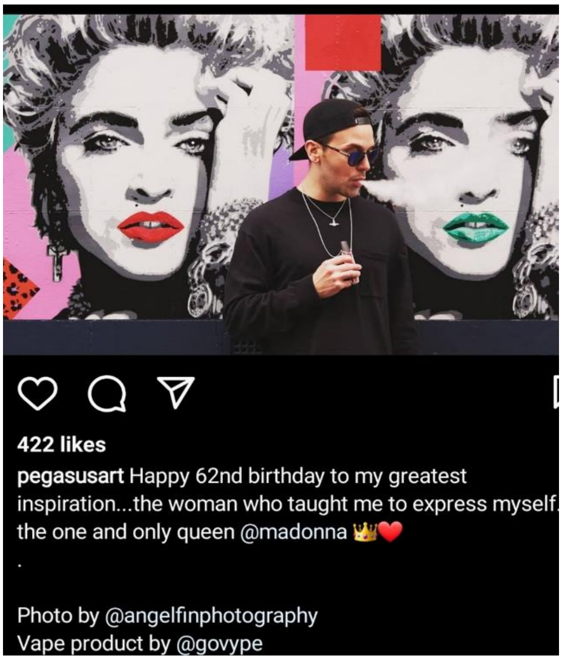

Slide 4

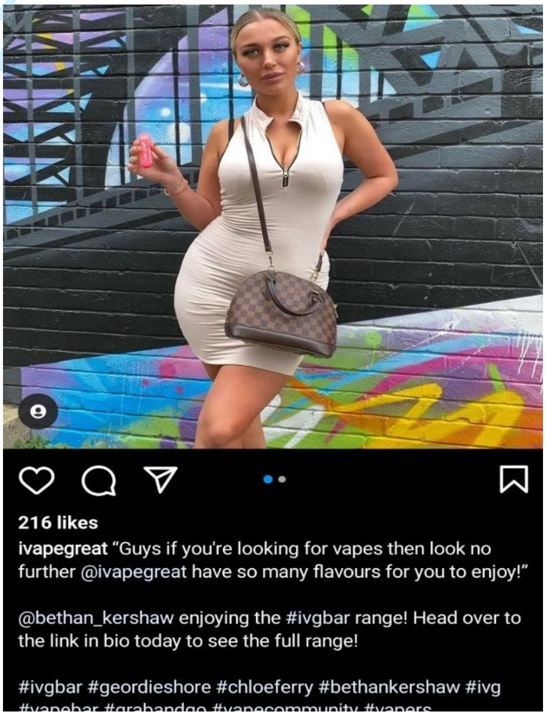

Slide 5

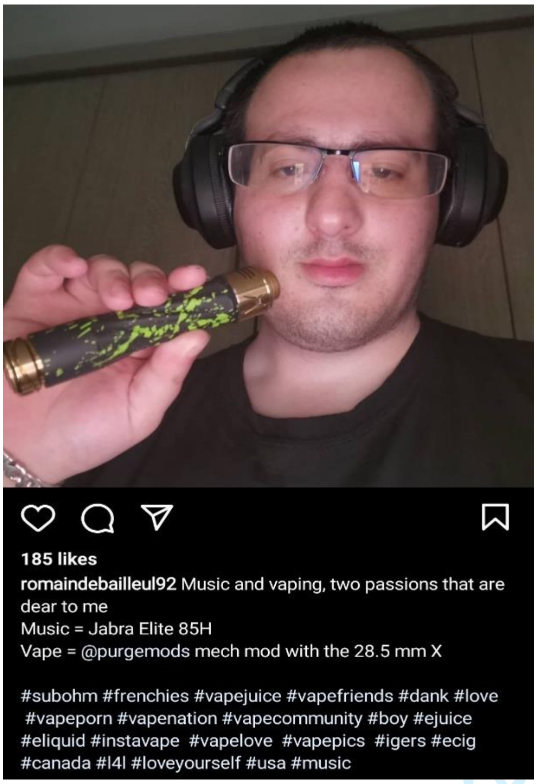

Slide 6

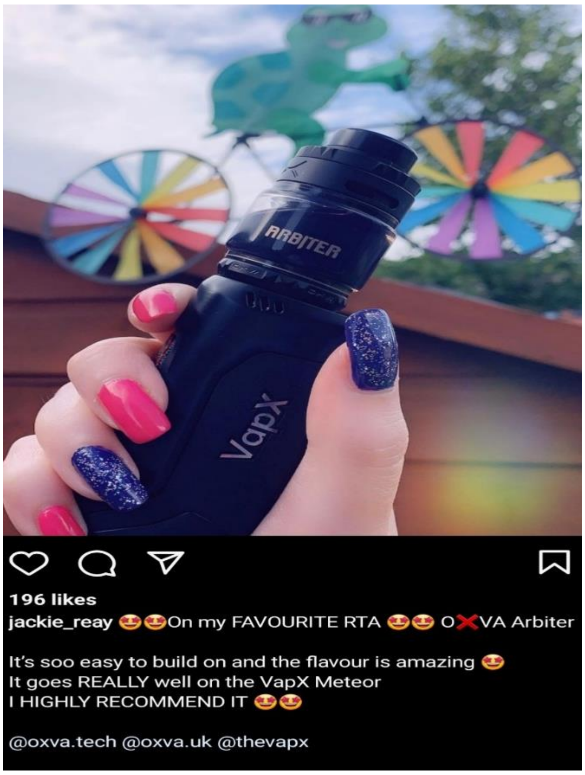

Slide 7

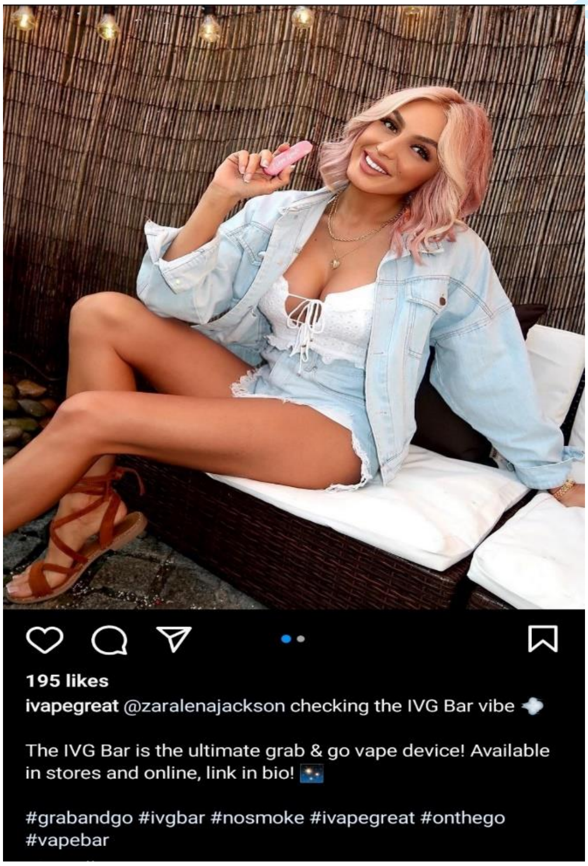

Slide 8

For peer review only - http://bmjopen.bmj.com/site/about/guidelines.xhtml

1. What can you remember from the images that you just saw?
2. Do any of these images grab your attention?
    a. PROMPT: Why?
    b. PROMPT: What do you think about the use of colour in the images?
    c. PROMPT: What do you think about the number of likes each picture has?
    d. PROMPT: What do you think about the caption under the images?
3. Did the images have anything in common?
    a. If so, what?
4. Did you recognise anyone in the images?
    a. If so, who?
5. Why do you think these images have been posted?
6. What do you think the purpose of the images?
7. What do you think the image is trying to say?
    a. PROMPT: How do you know this?
8. Who do you think these images are targeted at?
    a. PROMPT: Why do you say that?
9. Do you think these images are selling a product?
    a. PROMPT: How can you tell if a product is being promoted or not?
    b. PROMPT: How do you know which product(s) are being promoted?
10. Who do you think has posted the images I showed you?
    a. Do you think that person is being paid to post the image or not?
    b. How can you tell if someone is being paid or not?
    c. Why do you think influencers would want to advertise e-cigarette products?
    d. Why do you think companies would pay influencers to advertise their products?

Show slides 9-12 of PowerPoint presentation.

For peer review only

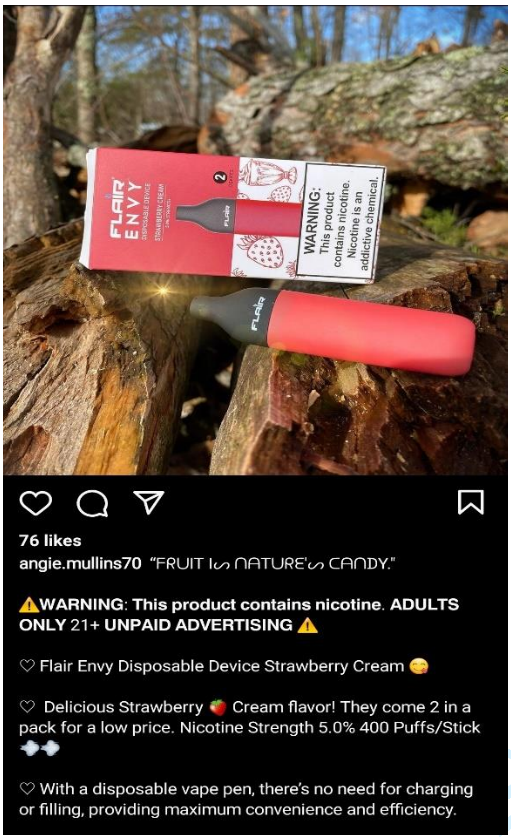

Slide 9

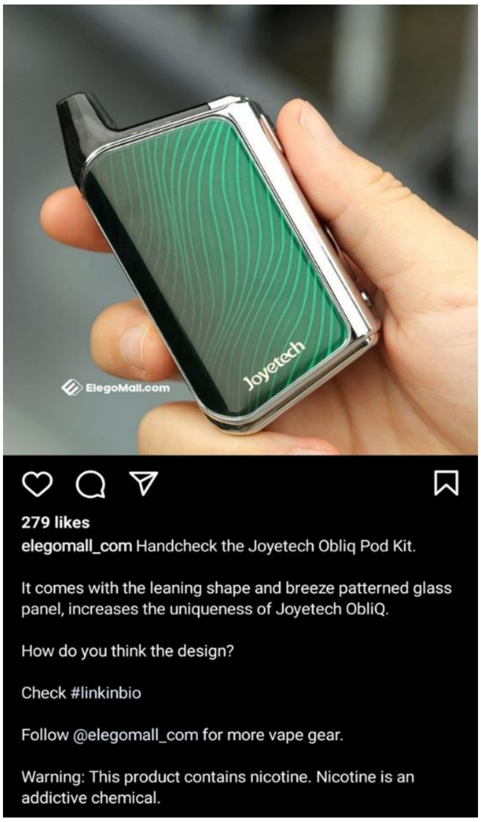

Slide 10

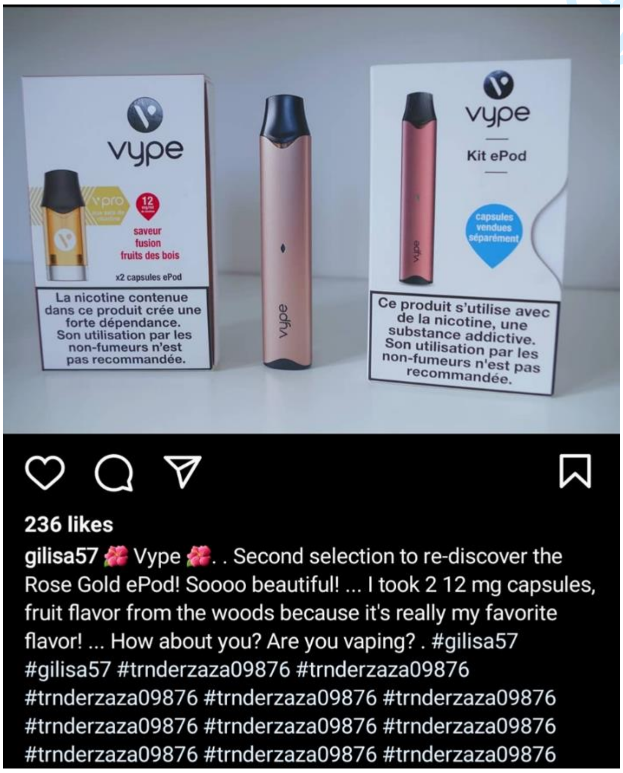

Slide 11

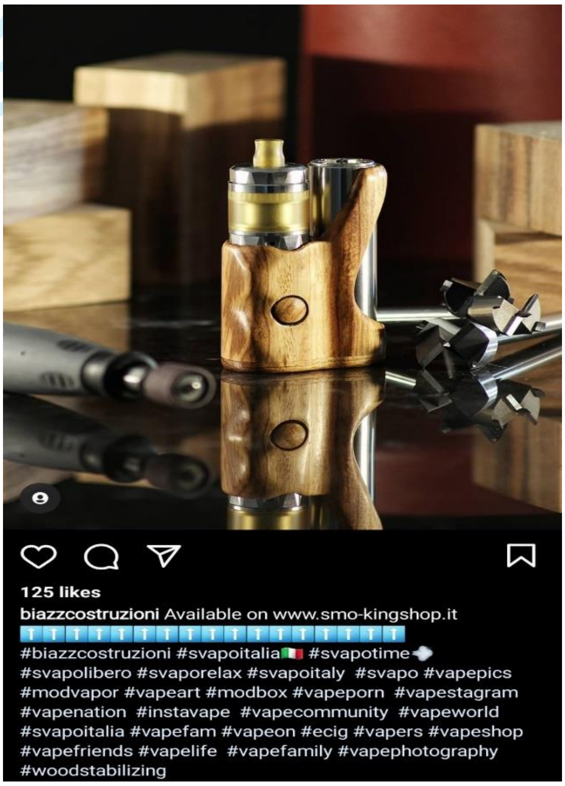

Slide 12

1. What can you remember from the images that you just saw?
2. Do any of these images grab your attention?
    a. PROMPT: Why?
    b. PROMPT: What do you think about the use of colour in the images?
    c. PROMPT: What do you think about the number of likes each picture has?
    d. PROMPT: What do you think about the caption under the images?
3. Did the images have anything in common?
    a. If so, what?
4. Do any of these images remind you of any other products?
    a. PROMPT: Why?
5. Why do you think these images have been posted?
6. What do you think the purpose of the images?
7. What do you think the image is trying to say?
    a. PROMPT: How do you know this?
8. Who do you think these images are targeted at?
    a. PROMPT: Why do you say that?
9. Why do you think e-cigarettes have been designed this way?

Show slides 14-18 of the PowerPoint presentation.

For peer review only

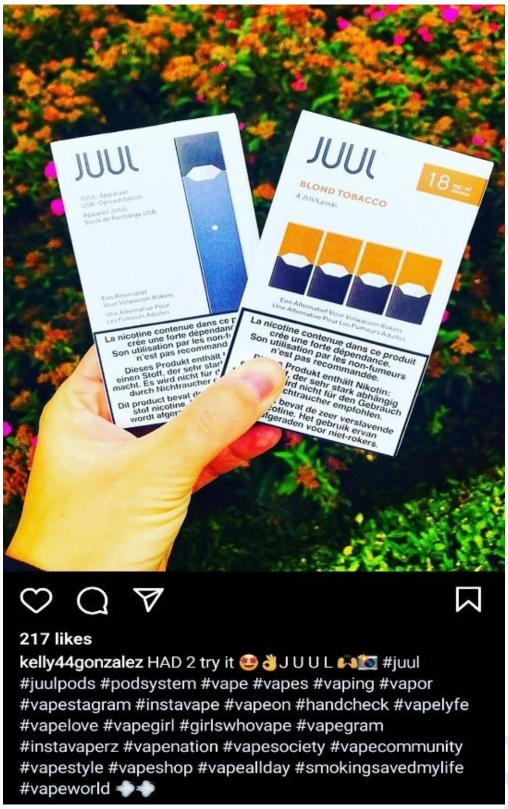

Slide 14

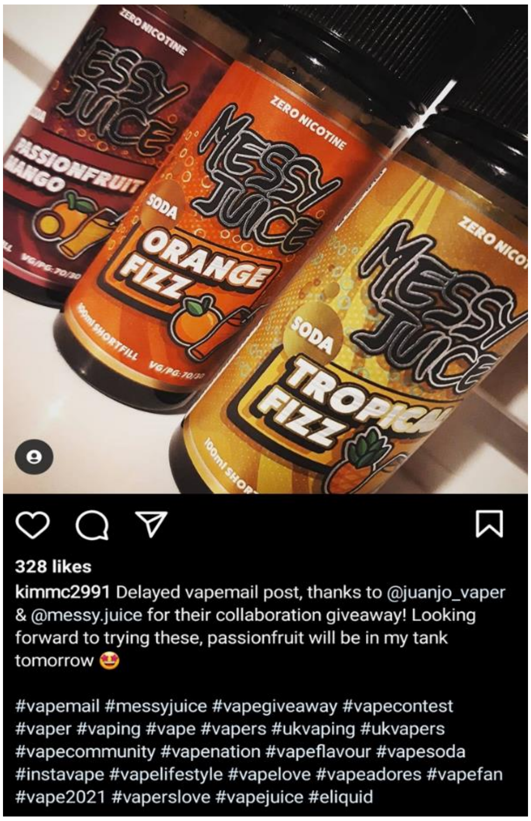

Slide 15

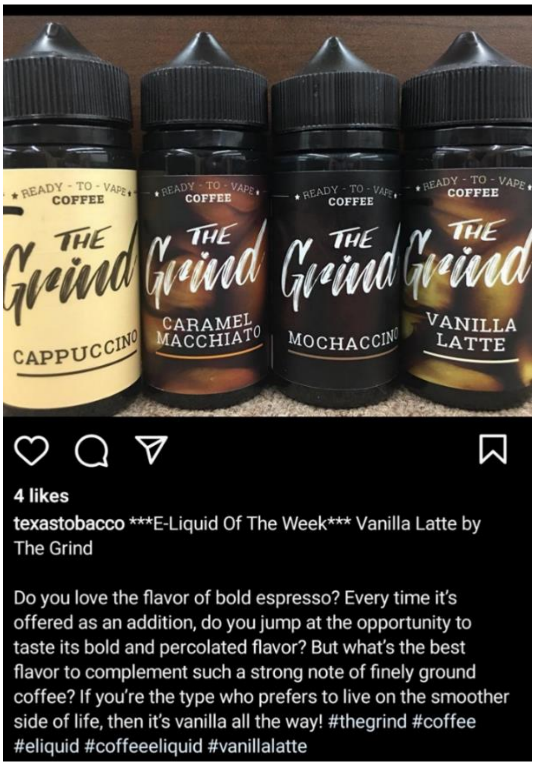

Slide 16

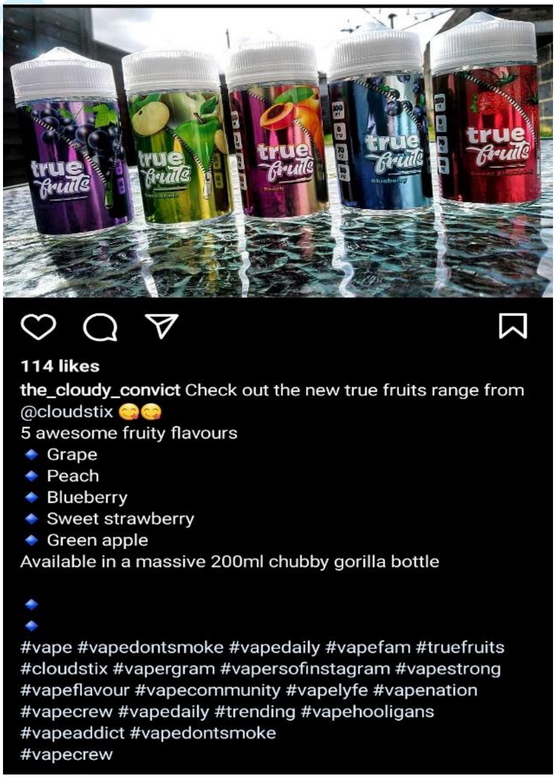

Slide 17

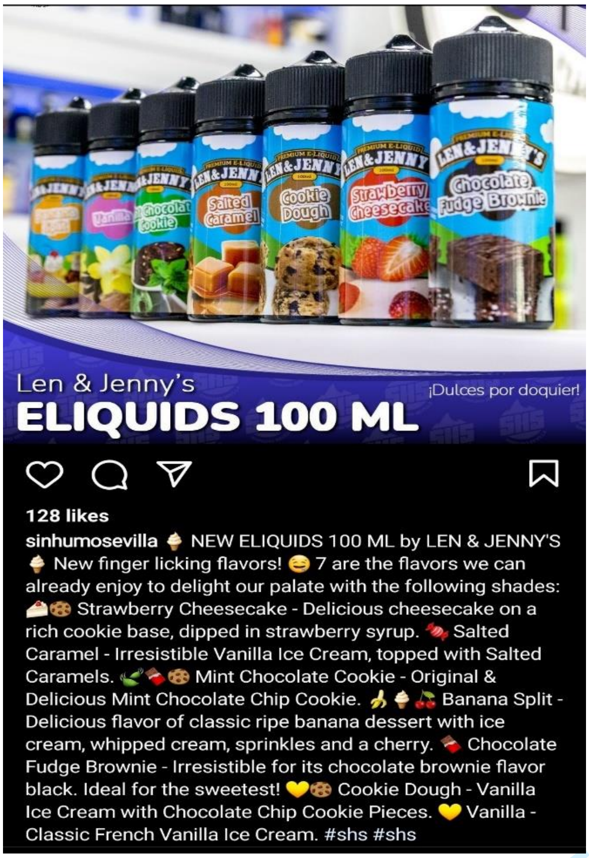
Salted Caramel
Cookie Dough
Strawberry Cheesecake
Chocolate Fudge Brownie
Len & Jenny's
ELIQUIDS 100 ML
¡Dulces por doquier!
128 likes
sinhumosevilla NEW ELIQUIDS 100 ML by LEN & JENNY'S New finger licking flavors! 7 are the flavors we can already enjoy to delight our palate with the following shades: Strawberry Cheesecake - Delicious cheesecake on a rich cookie base, dipped in strawberry syrup. Salted Caramel - Irresistible Vanilla Ice Cream, topped with Salted Caramels. Mint Chocolate Cookie - Original & Delicious Mint Chocolate Chip Cookie. Banana Split - Delicious flavor of classic ripe banana dessert with ice cream, whipped cream, sprinkles and a cherry. Chocolate Fudge Brownie - Irresistible for its chocolate brownie flavor black. Ideal for the sweetest! Cookie Dough - Vanilla Ice Cream with Chocolate Chip Cookie Pieces. Vanilla - Classic French Vanilla Ice Cream. #shs #shs

Slide 18

1. What can you remember from the images that you just saw?
2. Do any of these images grab your attention?
    a. PROMPT: Why?
    b. PROMPT: What do you think about the colour of the products?
    c. PROMPT: What do you think about the number of likes each picture has?
    d. PROMPT: What do you think about the caption under the images?
3. Did the images have anything in common?
        i. If so, what?
4. Do any of these images remind you of any other products?
    a. PROMPT: Why?
5. Do you think these images are selling a product?
    a. Do you know what the products are that shown in the images?
        i. If yes, how do you know that?
6. Why do you think these images have been posted?
7. What do you think the purpose of the images?
8. What do you think the image is trying to say?
    a. PROMPT: How do you know this?
9. Who do you think these images are targeted at?
    a. PROMPT: Why do you say that?
10. What do you think of the flavours shown?
11. Do you see some flavours as more appealing compared to others?
    a. PROMPT: What ones and why?
12. Do you think there are any flavours that are more appealing to youths compared to adults?
    a. PROMPT: What ones and why?

Personal experiences

I am now moving on to ask about your own experiences and of friends and other people.

1. Have you seen any information about e-cigarette on social media platforms?
    a. What kind of information was it?
        i. PROMPT: Why do you think the images were posted?
        ii. PROMPT: What do you think the images were trying to show/tell you?
2. Do friends or anyone you follow on social media post or share messages about e-cigarettes?
    a. How does that make you feel about them?
    b. Why do you think they post or share these messages?
    c. If seeing a friend or someone you know post about e-cigarettes on social media, would it make you want to share images or messages?

For peer review only - http://bmjopen.bmj.com/site/about/guidelines.xhtml

d. Out of interest have you or your friends ever posted on social media about e-cigarettes?

Warnings on social media posts

1. Play video (short clip) https://www.youtube.com/watch?v=tk9SQDH_ZNo
    a. Did you notice anything about the short clip I just showed you?
    b. Can you describe what you remember from the clip I just showed you?
    c. IF NECESSART PROMPT: Did anyone notice the warning notice?
    d. PROMPT: What did it say?
    e. PROMPT: What did you think about it?
2. Replay video pointing out warning.
    a. What do you think about the length of time that the warning was shown for?
        i. How easy or difficult was it to notice the warning?
3. Why do you think there are warnings on posts/videos that show e-cigarette products?
4. Do you think there should or should not be warnings on posts/videos show e-cigarette products?
    a. PROMPT: Why?

Final remarks

1. Have you ever noticed the marketing/promotion of e-cigarette products before or not?
2. How do you think about the marketing/promotion of e-cigarette products on social media?

Close interview

- Is there anything else you would like to add, that we haven't already talked about?
- Thank you very much for taking part.

For peer review only

Standards for Reporting Qualitative Research (SRQR)*

http://www.equator-network.org/reporting-guidelines/srqr/

Title and abstract

	Page/line no(s).
**Title** - Concise description of the nature and topic of the study Identifying the study as qualitative or indicating the approach (e.g., ethnography, grounded theory) or data collection methods (e.g., interview, focus group) is recommended	1
**Abstract** - Summary of key elements of the study using the abstract format of the intended publication; typically includes background, purpose, methods, results, and conclusions	2

Introduction

**Problem formulation** - Description and significance of the problem/phenomenon studied; review of relevant theory and empirical work; problem statement	4 and 5
**Purpose or research question** - Purpose of the study and specific objectives or questions	5

Methods

**Qualitative approach and research paradigm** - Qualitative approach (e.g., ethnography, grounded theory, case study, phenomenology, narrative research) and guiding theory if appropriate; identifying the research paradigm (e.g., postpositivist, constructivist/ interpretivist) is also recommended; rationale**	6 and 7
**Researcher characteristics and reflexivity** - Researchers' characteristics that may influence the research, including personal attributes, qualifications/experience, relationship with participants, assumptions, and/or presuppositions; potential or actual interaction between researchers' characteristics and the research questions, approach, methods, results, and/or transferability	7, 18 and 19
**Context** - Setting/site and salient contextual factors; rationale**	6 and 7
**Sampling strategy** - How and why research participants, documents, or events were selected; criteria for deciding when no further sampling was necessary (e.g., sampling saturation); rationale**	6 and 7
**Ethical issues pertaining to human subjects** - Documentation of approval by an appropriate ethics review board and participant consent, or explanation for lack thereof; other confidentiality and data security issues	7
**Data collection methods** - Types of data collected; details of data collection procedures including (as appropriate) start and stop dates of data collection and analysis, iterative process, triangulation of sources/methods, and modification of procedures in response to evolving study findings; rationale**	6 and 7
**Data collection instruments and technologies** - Description of instruments (e.g., interview guides, questionnaires) and devices (e.g., audio recorders) used for data collection; if/how the instrument(s) changed over the course of the study	6 and 7
**Units of study** - Number and relevant characteristics of participants, documents, or events included in the study; level of participation (could be reported in results)	6 and 7

For peer review only - http://bmjopen.bmj.com/site/about/guidelines.xhtml

**Data processing -** Methods for processing data prior to and during analysis, including transcription, data entry, data management and security, verification of data integrity, data coding, and anonymization/de-identification of excerpts	6 and 7
**Data analysis -** Process by which inferences, themes, etc., were identified and developed, including the researchers involved in data analysis; usually references a specific paradigm or approach; rationale**	6 and 7
**Techniques to enhance trustworthiness -** Techniques to enhance trustworthiness and credibility of data analysis (e.g., member checking, audit trail, triangulation); rationale**	6 and 7

Results/findings

**Synthesis and interpretation -** Main findings (e.g., interpretations, inferences, and themes); might include development of a theory or model, or integration with prior research or theory	8-15
**Links to empirical data -** Evidence (e.g., quotes, field notes, text excerpts, photographs) to substantiate analytic findings	8-15

Discussion

**Integration with prior work, implications, transferability, and contribution(s) to the field -** Short summary of main findings; explanation of how findings and conclusions connect to, support, elaborate on, or challenge conclusions of earlier scholarship; discussion of scope of application/generalizability; identification of unique contribution(s) to scholarship in a discipline or field	15-19
**Limitations -** Trustworthiness and limitations of findings	18 and 19

Other

**Conflicts of interest -** Potential sources of influence or perceived influence on study conduct and conclusions; how these were managed	20
**Funding -** Sources of funding and other support; role of funders in data collection, interpretation, and reporting	20

*The authors created the SRQR by searching the literature to identify guidelines, reporting standards, and critical appraisal criteria for qualitative research; reviewing the reference lists of retrieved sources; and contacting experts to gain feedback. The SRQR aims to improve the transparency of all aspects of qualitative research by providing clear standards for reporting qualitative research.

**The rationale should briefly discuss the justification for choosing that theory, approach, method, or technique rather than other options available, the assumptions and limitations implicit in those choices, and how those choices influence study conclusions and transferability. As appropriate, the rationale for several items might be discussed together.

Reference:
O'Brien BC, Harris IB, Beckman TJ, Reed DA, Cook DA. **Standards for reporting qualitative research: a synthesis of recommendations.** *Academic Medicine*, Vol. 89, No. 9 / Sept 2014
DOI: 10.1097/ACM.0000000000000388

For peer review only - http://bmjopen.bmj.com/site/about/guidelines.xhtml

For peer review only

For peer review only - http://bmjopen.bmj.com/site/about/guidelines.xhtml

BMJ Open

Youth's exposure to and engagement with e-cigarette marketing on social media: a UK focus group study

Journal:	*BMJ Open*
Manuscript ID	bmjopen-2022-071270.R2
Article Type:	Original research
Date Submitted by the Author:	28-Apr-2023
Complete List of Authors:	Smith, Marissa; University of Glasgow, MRC/CSO Social and Public Health Sciences Unit<br>Hilton , Shona; University of Glasgow, MRC/CSO Social and Public Health Sciences Unit
**Primary Subject Heading**:	Public health
Secondary Subject Heading:	Qualitative research
Keywords:	PUBLIC HEALTH, QUALITATIVE RESEARCH, Health policy < HEALTH SERVICES ADMINISTRATION & MANAGEMENT

SCHOLARONE™
Manuscripts

BMJ

I, the Submitting Author has the right to grant and does grant on behalf of all authors of the Work (as defined in the below author licence), an exclusive licence and/or a non-exclusive licence for contributions from authors who are: i) UK Crown employees; ii) where BMJ has agreed a CC-BY licence shall apply, and/or iii) in accordance with the terms applicable for US Federal Government officers or employees acting as part of their official duties; on a worldwide, perpetual, irrevocable, royalty-free basis to BMJ Publishing Group Ltd ("BMJ") its licensees and where the relevant Journal is co-owned by BMJ to the co-owners of the Journal, to publish the Work in this journal and any other BMJ products and to exploit all rights, as set out in our licence.

The Submitting Author accepts and understands that any supply made under these terms is made by BMJ to the Submitting Author unless you are acting as an employee on behalf of your employer or a postgraduate student of an affiliated institution which is paying any applicable article publishing charge ("APC") for Open Access articles. Where the Submitting Author wishes to make the Work available on an Open Access basis (and intends to pay the relevant APC), the terms of reuse of such Open Access shall be governed by a Creative Commons licence – details of these licences and which Creative Commons licence will apply to this Work are set out in our licence referred to above.

Other than as permitted in any relevant BMJ Author's Self Archiving Policies, I confirm this Work has not been accepted for publication elsewhere, is not being considered for publication elsewhere and does not duplicate material already published. I confirm all authors consent to publication of this Work and authorise the granting of this licence.

For peer review only

Youth's exposure to and engagement with e-cigarette marketing on social media: a UK focus group study

Marissa J. Smith[1*] and Shona Hilton[1]

[1] MRC/CSO Social and Public Health Sciences Unit, University of Glasgow, Glasgow, G3 7HR, UK

*Corresponding author

Email: marissa.smith@glasgow.ac.uk Tel: (+44) 0141 353 7500

Keywords: Health policy, qualitative research, public health

For peer review only

ABSTRACT

Objective: E-cigarettes are promoted creatively through social media and considering the potential influence of social media marketing on young people, we explored young people's exposure to and engagement with social media marketing of e-cigarettes.

Design: Semi-structured discussion groups.

Subjects: Twenty focus groups with 82 young people aged 11-16 living in the Central belt of Scotland.

Methods: Youths were asked about smoking and vaping behaviours, social media use, vaping advertisement exposure, and were shown illustrative examples of social media content (e.g., images and videos) about different messages, presentations, and contextual features. Transcripts were imported into NVivo 12, coded thematically and analysed.

Results: Youths highlighted a variety of tactics e-cigarette companies use, including influencer or celebrity endorsement, attractive youth flavours, bright colours and emotional appeal to advertise and promote their products directly to young people. Social media influencers who advertise e-cigarettes were described as portraying e-cigarettes as 'cool' and 'fashionable' to entice viewers to try the products. Youths considered that there is a need for more restrictions on social media content to protect youths while also still allowing smokers to purchase them as a cessation device.

Conclusions: Our study highlights that the e-cigarette industry is using previously employed tactics similar to the tobacco industry to advertise and promote its products on social media. These findings suggest the growing need for governments to work together to develop and implement policies to restrict the advertising and marketing of e-cigarettes on social media.

For peer review only

Strengths and limitations of this study

- This study is one of the first in-depth studies to explore youths' responses to and engagement with e-cigarette marketing on social media with a focus on the rise in user-generated and influencer content.
- This research is very timely as governments consider the need for further e-cigarette regulations.
- A methodological strength is the rich dataset from semi-structured interviews with 82 youths aged 11-16, facilitating understandings of youths' exposure to and interaction with e-cigarette marketing on social media.
- Qualitative thematic analysis of the data allows depth of opinions but cannot offer predictions about the frequency of specific opinions with a wider population.

For peer review only

For peer review only - http://bmjopen.bmj.com/site/about/guidelines.xhtml

INTRODUCTION

Marketing strategies used by electronic cigarette (e-cigarette) companies have been associated with the uptick in e-cigarette use (vaping) among youth [1]. Many studies have found that the promotion of e-cigarettes through various channels (e.g., television (TV), social media influencers) has lent to increased positive perceptions of vaping and intentions to use vaping products and contributed to e-cigarette uptake among youth [2-4].

In the UK, the advertising of e-cigarettes is regulated through Article 20(5) of the revised European Union (EU) Tobacco Products Directive (2014/40/EU) (TPD), [5] which was transposed into UK law by the Tobacco and Related Products Regulations (TRPR) 2016 [6]. The TPD prohibited the advertising of nicotine-containing e-cigarettes (unless licensed as medicines) in channels with potential cross-border impact (i.e. channels that show adverts or sponsored events that originate from non-EU countries in EU countries), including TV, radio, newspapers, magazines, and sponsorship [7]. Online advertising was also prohibited under the TPD, although the regulations left scope for marketers to retain websites containing factual information about e-cigarette products. As there are currently no medicinally licensed nicotine vaping products in the UK, the prohibitions apply to all nicotine-containing e-cigarette products on the market. The TRPR requirements for e-cigarette advertising were set out in 2017 and are enforced by the Committee of Advertising Practice (CAP) – a self-regulatory body of organisations representing advertising, direct marketing, media businesses, and sales promotion endorsed and administered by the independent Advertising Standards Authority (ASA) [8, 9]. Although Rule 22.12 of the ASA CAP code prohibits advertising in online media, social media content for e-cigarettes is permitted in “non-paid-for space online under the marketer’s control, provided claims are factual rather than promotional” [10]. Promotional content includes descriptive language that goes beyond objective, factual claims, significant imagery that is not related to the product, and health claims (e.g. that e-cigarettes are safer or healthier than tobacco) [10].

Social media marketing includes promotions by means of paid digital advertising, via compensated ‘influencers’ (individuals with large followings who are paid to advertise brands and products), and on brand pages that companies can create for free and use to share content [11]. Most adolescents use

For peer review only

visual-based social media daily. User-generated and influencer marketing content on social media represents a key influence on young people's understanding of products, including e-cigarettes [12]. E-cigarettes are promoted creatively through social media, with well-designed features including colours, flavour variations, emotional appeal, incentives (such as price promotions and discount vouchers), and even celebrity endorsements [13]. Research has shown that online marketing that leads to exposure to e-cigarette advertising, including where it is concealed as information or recommendations from peers, can increase the likelihood of vaping in individuals, including among youths [14-16]. In addition, a number of researchers have suggested that e-cigarette advertising could lead to increases in how socially acceptable and desirable cigarettes are perceived to be, and subsequently influence their continued use or possible (re)uptake in smokers, e-cigarette users and dual-users [17-20]. E-cigarette companies have been promoting their products on social media in an attempt to expand their market [21, 22]. In addition, e-cigarette companies use social media platforms to promote e-cigarettes and create a socially attractive vaping image [23]. Previous studies [including 24, 25, 26] have explored youths' perceptions of e-cigarette marketing on social media, finding that social media adverts entice non-smokers to use e-cigarettes, emotional appeals are featured in adverts, use of vibrant colours in the packaging, appeal of flavours and advertisements portray e-cigarettes as less harmful than combustible cigarettes. Liu et al.[27] examined youths; perception of tobacco and marijuana messaging on social media and reported similar findings (including, appealing colours in the packaging and appealing flavours). One limitation of the literature in this area is that it focuses on either smokers, non-smokers and/or vapers. This leaves out non-vapers and dual-users (those who use both cigarettes and e-cigarettes), understudied. It is important to establish whether exposure to e-cigarette adverts on social media influence attitudes towards e-cigarettes in non-vapers and smoking in dual-users.

Gaining a better understanding of the nature, extent, and impact of e-cigarette marketing including their possible effects on youth e-cigarette perceptions and use can help guide the development of policies and interventions to educate youths about e-cigarettes [28]. This study, therefore, aims to explore youths', including non-smokers and non-vapers and dual-users exposure to, and engagement with e-cigarette advertising on social media, including user-generated and influencer content.

For peer review only

METHODS

We conducted 20 focus groups in Scotland between March and May 2022. Focus groups included between three and five participants (a total of 82 participants). Purposive sampling was used to recruit a diverse sample of youths in terms of age, sex, socio-economic background, smoking status, and vaping status [29]. Eleven groups were recruited through youth workers in local youth organisations. These gatekeepers handed out information sheets and helped achieve the sampling frame in terms of youth demographics and experiences concerning combustible cigarettes and e-cigarettes. The three organisations that helped with participant recruitment worked specifically with young people from disadvantaged backgrounds in urban areas. This recruitment strategy resulted in the inclusion of a range of participants from more and less deprived backgrounds and with experiences of smoking and vaping. Seven groups were recruited through the Schools Health and Wellbeing Improvement Research Network (SHINE) Newsletter which is distributed monthly to over 500 schools in Scotland. The remaining two groups were recruited via personal networks directly by MS.

Focus group discussions were facilitated to allow the research team to explore opinions on online advertising and marketing of e-cigarettes. Friendship groups of 3–5 participants were used to facilitate in-depth insights and promote participant interaction. Each participant was given a £20 shopping voucher as compensation for their time.

Prior to the start of the focus groups, participants completed a short anonymous questionnaire about their age, biological sex, postcode, smoking, and e-cigarette use status. For both combustible cigarettes and e-cigarettes, the questionnaire asked participants to specify whether they had tried or used them in the past or were using them at the time of the study. Based on a scoping review of user-generated content and influencer marketing involving e-cigarettes on social media [30], a topic guide (Appendix A) was developed which covered three key areas, including use of social media, perceptions of social media influencers versus user-generated e-cigarette content on social media, and views on the marketing of e-cigarettes on social media.

For peer review only

Images of different types of e-cigarettes (‘tanks’, disposables, and pod devices) and e-liquids posted by social media influencers and general users were used as conversation starters found during the scoping review part of this research [30], were used as conversation starters. Group discussions were facilitated by MS (a young post PhD early career researcher post and experienced qualitive researcher). Ten of the groups were conducted online using Microsoft Teams and ten of the groups were conducted face-to-face. Of these, one of the groups was conducted on the youth organisation’s premises, and the other nine were conducted at the school, with representatives of the youth organisation present. Groups lasted between 40 and 66 minutes. Field-notes reflecting on the focus group and individual issues discussed were written up for each group. All focus groups were audio recorded (with participants' permission) and transcribed verbatim.

Analysis

Following Braun and Clarke’s six-phase framework for thematic analysis [31], we conducted thematic analysis of the data form the focus groups. The research team read and re-read the transcripts to become familiar with the data, and then iteratively constructed a coding frame to enable consistent organisation of relevant data. NVivo was used to organise categories on the basis of inductive themes that emerged from close reading of the, capture of both areas of agreement and less typical perspectives across a range of categories. Each transcript was imported into NVivo 12, coded independently, cross-checked, and analysed by MS and SH (professor of public health and experienced qualitive researcher). Contradictory cases and group dynamics were discussed, making use of transcripts and field notes. The researchers reflected on their role as researcher, remained constantly aware of their position and took care not to introduce bias throughout the research. To further reduce bias the researcher (MS) recorded the focus groups and analysed them some time after they were completed ensuring a more reflective viewpoint of occurrences. Ethical approval for the study was obtained from the University of Glasgow's Medical and Veterinary Life Sciences Ethics Committee (reference 200210034).

For peer review only

Patient and Public Involvement

Patients and/or the public were not involved in the design of the study, developed of the research questions, recruitment, outcome measures or conduct of the study. A summary of the results will be disseminated to study participants.

RESULTS

Participant characteristics

Eighty-two youths aged 11–16 years participated (47 females (57%) and 35 males (43%)), representing diversity in sociodemographic characteristics and smoking-related behaviours. Age distribution within the sample was skewed slightly towards 14–15-year-olds, with 14-year-olds making up the largest subgroup (n= 24). While the majority of participants did not currently smoke or use e-cigarettes, the sample included 10 smokers and 18 youths who used e-cigarettes. Deprivation rank was assigned using the Scottish Index of Multiple Deprivation (SIMD) [32]. The area-level deprivation grouped using a binary deprivation variable (least deprived/most deprived) in which the three most deprived quintiles were grouped into the most deprived category for the Central Belt of Scotland area. Table 1 describes the focus group composition and participants in more detail and Table 2 summarises smoking and e-cigarette use among the sample.

For peer review only

For peer review only - http://bmjopen.bmj.com/site/about/guidelines.xhtml

Group	Area-level deprivation	Sex	Age	Cigarette Smoker	E-cigarette use
1	Least deprived	Female	13-15	Never	Never
2	Least deprived	Female	14-15	Never	Mixed – Never (4) / Tried (1)
3	Least deprived	Female	13-16	Never	Mixed – Never (2) / Tried (1)
4	Most deprived	Mixed – Male (3)/Female (2)	12-15	Mixed – Never (3) / Current (2)	Mixed – Never (3) / Tried (1)/ Current (1)
5	Most deprived	Mixed – Male (1)/ Female (4)	14-16	Mixed – Never (2)/ Tried (2)/ Current (1)	Mixed – Never (2)/ Tried (2)/ Current (1)
6	Most deprived	Male	12-15	Never	Never
7	Most deprived	Male	16	Current	Current
8	Least deprived	Mixed – Male (2)/Female (3)	14	Never	Never
9	Most deprived	Male	16	Mixed – Tried (1)/ Current (2)	Current
10	Most deprived	Mixed – Male (4)/Female (1)	14-15	Mixed – Never (3)/ Tried (1)/ Current (1)	Mixed – Never (3)/ Tried (1)/ Current (1)
11	Most deprived	Mixed – Male (3)/Female (2)	13-16	Mixed – Never (2)/ Tried (2)/ Current (1)	Mixed – Never (1)/ Current (4)
12	Least deprived	Mixed – Male (2)/Female (1)	15-16	Tried	Mixed – Tried (2)/ Current (1)
13	Least deprived	Female	13-16	Never	Never
14	Most deprived	Mixed – Male (1)/Female (3)	11-12	Never	Never
15	Most deprived	Mixed – Male (3)/Female (1)	11-12	Never	Never
16	Most deprived	Mixed – Male (2)/Female (2)	11-12	Never	Never
17	Most deprived	Female	14-16	Mixed – Never (4)/ Tried (1)	Mixed – Never (1)/ Tried (1)/ Current (3)
18	Most deprived	Male	13-16	Never	Never
19	Most deprived	Female	14	Never	Mixed – Tried (2)/ Current (1)
20	Least deprived	Female	15-16	Never	Tried (3)

Table 1: Focus group location, participants, and their cigarette smoking and e-cigarette use.

For peer review only

	E-cigarette use											
	Never			Tried			Current			Total		
**Cigarette smoker**	**n**	**(col %)**	**(row %)**	**n**	**(col %)**	**(row %)**	**n**	**(col %)**	**(row %)**	**n**	**(col %)**	**(row %)**
**Never**	51	98.1%	82.3%	7	58.3%	11.3%	4	22.2%	6.5%	62	75.6%	100.0%
**Tried**	1	1.9%	10.0%	4	33.3%	40.0%	5	27.8%	50.0%	10	12.2%	100.0%
**Current**	0	0.0%	0.0%	1	8.3%	10.0%	9	50.0%	90.0%	10	12.2%	100.0%
**Total**	52	1	92.3%	12	100.0%	14.6%	18	100.0%	22.0%	82	100.0%	100.0%

Table 2: E-cigarette use according to cigarette smoking.

For peer review only

Perceptions of advertising tactics used by the e-cigarette industry to target youths

Youths discussed three ways (influencer or celebrity endorsement, flavourings and emotional appeal) e-cigarette companies market their products to target young people.

Influencer or celebrity endorsements

During the focus groups, participants frequently discussed the prevalence of social media influencers advertising e-cigarette products. When discussing why e-cigarette and/or tobacco companies would want to use social media influencers to advertise their products, participants highlighted the popularity of social media and how this would increase the reach of the adverts.

"I guess companies would use them [influencers] because the company may have approached them to advertise the product and they know they might have a huge following on the platform, so they might influence some more people to buy the products." (Male, current smoker, current vaper)

"More people are on social media nowadays. So, it's better that they [vaping/tobacco companies] use influencers to advertise their products compared to say in newspapers, 'cause you don't see people reading newspapers anymore." (Male, never smoker, never vaper)

Participants reported seeing marketing and advertising of e-cigarettes on social media and that they were portrayed as 'cool' and 'fashionable' by social media influencers.

"If an influencer posts it, a lot of people see it and be like, that's the trend now, it's a cool thing to do, so I want to take part in that." (Female, never smoker, tried vaping)

For peer review only - http://bmjopen.bmj.com/site/about/guidelines.xhtml

"It's like they're showcasing them. It's not just like it's in the background or whatever, it's not a normal pose to have them in, it's like they're showcasing them and that they are a fashionable thing to carry or to have." (Female, never smoker, never vaper)

Participants discussed that this would influence the viewer to try the product, thus increasing the prevalence of use.

"People get tempted easily by seeing the post. Like if they saw some influencers trying that stuff [e-cigarettes], people get tempted quickly to try that stuff, because then that person is trying it so the like the kids would think this is something that I should also try that stuff like that, that's the point." (Male, current smoker, current vaper)

"When you see what an influencer is, most people think oh if they're doing that, then it's cool, so it like, it's making more people want to buy the vapes, and stuff." (Female, never smoker, tried vaping)

Several participants discussed how social media posts make it too easy for underage youths to purchase and hide the fact you are buying e-cigarette products from websites.

"I've seen a thing on TikTok like, they [influencers] show you them putting them [e-cigarettes] in the wee boxes and all that, or you could put them in secret packaging like behind the lashes. Like you can order it off their website and they'll hide it in the packaging, they put a few bits of sweeties on top of your vapes so your mum doesn't see it." (Female, tried smoking, current vaper)

Using sweet and fruity flavours to appeal to youths

For peer review only

Participants discussed the variety of flavours of e-cigarettes available, highlighting that the sweet and fruity flavours are particularly attractive to youths.

> *“’Cause younger people, like us would think that the flavoured ones look nice so they would try then and then they’d start vaping.” (Male, never smoker, never vaper)*

> *“I think the type of ice cream flavours or sweetie ones are targeted at younger people because most people in their 30s and 40s would probably use the tobacco one or the coffee ones.” (Male, current smoker, current vaper)*

> *“The ones that stood out to me the most were the Ben and Jerry’s and fruit ones compared to the tobacco ones. I feel like, if I was going to buy them, I would buy them ‘cause they are attractive and are things I like.” (Female, tried smoking, current vaper)*

Several participants stated that the variety of flavourings was to appeal to youths.

> *“I feel like no one really wants to taste tobacco, to be honest. But I think putting out there with like grape and blueberry flavourings are going to attract younger people to the marketplace. I think it’s an intentional design and I feel like that is probably what it does attract youths.” (Female, never-smoker, never vaper)*

Using vibrant colours to attract youth attention

In addition, participants spoke about the presentation of e-cigarette products on social media, for example, the use of vibrant colours and filters.

For peer review only - http://bmjopen.bmj.com/site/about/guidelines.xhtml

“You can tell by the picture they're promoting them. Because if it was not promoting them you would have like a red colour, it's like a stop, it's kind of like a no colour. But they are using really positive colours, like pink and green. It makes you stop and look at it to see what it’s [the social media post] is about.” (Male, never smoker, never vaper)

“It was really bright colours and they had bright filters on them so it is brighter. If you were scrolling past then gives you a bright colour, you’d go back and see it. You'd be like, oh what’s that? You’d be, like, drawn to it. […] I think young people like us would be drawn to that, well I would.” (Female, never smoker, never vaper)

Connecting emotionally with youths

Participants discussed the emotional connections and positive portrayal of e-cigarettes on social media, stating that individuals in the social media posts ‘looked happy’ to be using the products.

“I think the person who posted this product, I think he or she was trying to emphasise the fact that these products are good because they look happy to be advertising it. So, you should try them and like they were not promoting vaping negatively or that it is bad at all. I think they were trying to sell the aesthetic idea of this product.” (Male, current smoker, current vaper)

“It’s the way they’re all holding them, they looked quite passionate with a smile on their face. A big cheesy smile.” (Male, current smoker, current vaper)

For peer review only

Participants highlighted the use of emojis in e-cigarette social media posts. Emojis (icons used to express an idea or emotion) and emoji sequencing (the use of two or more emojis that form a conceptual rhetorical unit) [33] are used to enhance the message meaning [34, 35]. When asked if this would affect their interaction with social media posts that included emojis, participants stated that they would be more inclined to read the post, rather than scrolling past.

"If you see it in colours, like emojis, you're like, oh I wonder what that is. You'd stop scrolling. 'Cause normally captions is just, like, text and a black background or something like that." (Female, never smoker, never vaper)

"The wee emojis they would draw my attention, because of how brightly coloured they are. And I think that's what they're trying to do, like, it'll draw your attention." (Female, never smoker, never vaper)

Perceptions of what can be done to limit e-cigarette marketing on social media

Participants had divergent views on the marketing and advertising of e-cigarettes on social media. Several participants stated that marketing and advertising should not be allowed as youths, including those of a similar age, could access this type of content and it may encourage them to try e-cigarettes.

"Advertising them [e-cigarettes] on social media is s a bad thing because younger people might see it and then try it." (Male, never smoker, never vaper)

I don't think it [advertising of e-cigarettes] should be allowed, just because of the younger community on it [social media]. And then if they are seeing them, they might end up doing it, getting addicted and end up dying." (Male, tried smoking, tried vaping)

For peer review only - http://bmjopen.bmj.com/site/about/guidelines.xhtml

But participants also acknowledged that controlling what is posted on social media is ‘tricky’ and ‘difficult’ and that prohibiting e-cigarette advertising may limit their role as cessation devices by smokers. Those participants suggested instead of prohibiting the marketing and advertising of e-cigarettes, warnings (such as age and health warnings) or other restrictive measures (such as parental controls or age restrictions) could be imposed on these types of social media posts.

“I think there needs to be age restrictions on the posts or parents could control what their child sees.” (Female, never smoker, never vaper)

DISCUSSION

E-cigarettes have become increasingly popular and visible in public life, particularly on social media. Our qualitative thematic analysis of youths’ perceptions of and engagement with e-cigarette advertising on social media highlighted a public health concern. Similar to previous research [36-38], youths in our study highlighted the tactics used by the e-cigarette industry to promote and advertise e-cigarettes to underage populations through influencer and celebrity endorsements, variety of flavours and using promotional endorsements. Youths discussed the positive portrayal of e-cigarettes by social media influencers, and they were enticing the viewer to buy the product. Youths highlighted that e-cigarette and/or tobacco companies use social media influencers to advertise their products on social media and by adverting on social media platforms, they will increase the reach of the adverts to a wider audience. We found that youths had divergent views on e-cigarette marketing on social media. Several participants stated that e-cigarette advertising on social media should be prohibited, citing the high prevalence of youths on social media who could access the content. While others suggested a balanced approach to regulating advertising on social media so that they can still be advertised as cessation devices by smokers. Youths suggested that all adverts should contain warnings (such as age and health warnings) or other restrictive measures (such as parental controls or age restrictions) to protect youths and non-users.

For peer review only - http://bmjopen.bmj.com/site/about/guidelines.xhtml

Previous studies which examined e-cigarette-related social media [24-26, 39-43] found that the vast majority of the content depicted positive attitudes towards vaping, while negative characterisations were mostly absent. Our study found that youths had similar experiences when exposed to e-cigarette content on social media. Consequently, there is a risk that youths will be exposed to, and possibly engage with, content that promotes vaping while staying uninformed about the negative aspects, including potential health harms. Our study adds to existing concerns about youths' perceptions of e-cigarettes as cool, fashionable products [44-47], by considering the advertising of the products by influencers on social media platforms, frequently accessed by youths. The use of social media influencers to promote products and celebrity endorsements encourage their followers to share and interact with their content, and ultimately purchase their products. Social media influencers often collaborate with multiple industries (e.g., fashion, and beauty products) in addition to e-cigarette products/brands. These influencers could potentially expose their non-e-cigarette-focused audience (including non-users of e-cigarettes) to e-cigarette content [48]. Therefore, these influencers could be considered an even higher risk for youth compared to influencers who post exclusively about e-cigarettes. This is problematic and concerning as most e-cigarette brands claim that their advertising and promotional content is meant to target current cigarette smokers to help them switch to e-cigarettes, [49, 50] rather than people who do not use nicotine at all. Previous literature has focused on either smokers, non-smokers and/or vapers, leaving out non-vapers and dual-users. Participants in our study were primarily non-smokers and non-vapers, we argue that it is important to explore non-smokers and non-vapers exposure to and engagement with e-cigarette adverts on social media. This is important for helping to determine the potential influence of e-cigarette adverts on social media in non-smokers and non-vapers.

Before plain packaging, the tobacco industry successfully used colourful packaging to represent its brand and increase the appeal of smoking [51-53]. The marketing tactics used by the tobacco industry were so effective that several e-cigarette manufacturers have used the same trade secrets to advertise their products [54-56]. Research has shown the plethora of bright-coloured packaging, eye-catching designs, and variety of flavourings available all appeal to youths and may result in experimentation of the products [57-62].

For peer review only

For peer review only - http://bmjopen.bmj.com/site/about/guidelines.xhtml

Previous research has illustrated YouTube viewers' exposure to e-cigarettes (as well as alcohol and tobacco) while watching videos on the platform [63]. Our study adds to this by illustrating the potential mechanisms by which exposure to e-cigarette advertising on social media platforms may influence perceptions and resultant trial/use of the products. While restrictions on mass media marketing of e-cigarettes are increasingly considered internationally, social media platforms are more difficult to manage because user-generated content (in particular) will not be covered by incoming regulations. Our study findings resonate with previous research, which found that youths were easily able to purchase e-cigarettes from the internet due to the absence of age-verification measures used by internet vendors [64, 65].

As with all research, our study has some limitations. First, and consistent with the qualitative design, the sample does not aim to be representative of UK youth, as our study focused on Scottish youths. However, we did have a diverse sample of both sexes. Secondly, the study's geographical remit has to be considered when interpreting the findings. The UK is considered an international leader in tobacco control policy. It is possible that participants' views may have been influenced by the UK's unique favourable policy approach to e-cigarettes and legal and socio-cultural context, including low smoking prevalence. Thirdly, using friendship groups may have influenced youth's honesty when answering questions and their responses. Youth's may not have wanted to disagree with their peers, thus responded in a similar way to the rest of the group. Fourthly, participants in this study were primarily non-smokers and non-vapers and it is possible this may have impacted in their discussion of e-cigarette advertisements on social media. However, as we used predominately visual prompts based upon data found during the scoping review of the research [30] we did not find that non-smokers and non-vapers contributed less to discussions. If we had not used visual prompts and focused on participants own experiences of what they had seen on social media (i.e., recalling from memory) this may have impacted non-smokers and non-vapers contribution to discussions. Fifthly, the data was collected in different formats (online and face-to-face), and this may have influenced participants' responses. Two of the online groups were conducted in a classroom with a teacher present, and during seven face-to-face groups, a teacher/youth worker was present in the room. The presence of a

For peer review only

teacher/youth worker may have influenced youth's willingness to answer questions and their responses. Finally, two of the groups were recruited through personal networks and this may have impacted the youth's responses.

The results from this study should provide guidance for future research. More research is needed to determine the most effective means to counter the favourable/positive aspects of e-cigarettes to reduce youths' interest in product trial and use. Future research could explore if there are different perceptions of e-cigarette exposure in social media between females and males. As females are one of the target groups for e-cigarette use [66, 67], it can reflect the marketing situation of e-cigarettes for females on social media, which deserves further study. In addition, research on the prevalence of warning statements on e-cigarette-related social media posts and the impact this has on youth perceptions would be an important future direction to expand this work.

Considering that most youth access multiple social media platforms multiple times per day [68, 69] and that exposure to this marketing is related to use [70, 71], our findings indicate a significant public health concern. Given the findings presented by our study, there is a growing need for policymakers to develop comprehensive plans to build more robust measures to protect youths and to restrict the ability of marketers to reach youths with enticing social media content promoting e-cigarettes. In addition, social media platforms should consider implementing more robust measures, such as age restrictions and portraying the negative aspects of vaping, to ensure the prevention of vaping-related content targeted at underage users.

CONCLUSION

The tactics used on social media platforms for advertising and marketing e-cigarettes directly to youths raises concerns and a new generation of youths becoming addicted to nicotine. The results of this study highlight that the e-cigarette industry is using previously employed tactics similar to the tobacco industry to advertise and promote its products on social media. Most youths use social media

For peer review only

daily and the depiction of products on social media represents a key influence on young people's understanding of products. These findings may highlight a priority for governmental policy to restrict the ability of marketers to reach youths with social media content promoting e-cigarettes.

Declarations

Authors' contributions: **Marissa J. Smith:** Conceptualisation, Data Curation, Investigation, Methodology, Validation, Visualisation**,** Writing- Original draft preparation. **Shona Hilton:** Conceptualisation, Methodology, Validation, Writing - Review & Editing.

Ethical approval: Ethical approval for the study was obtained from the University of Glasgow's Medical and Veterinary Life Sciences Ethics Committee (reference 200210034).

Competing interests: The authors declare that they have no competing interests.

Data sharing statement: All data relevant to the study are included in the article or uploaded as supplementary information**.**

Funding: MS acknowledges funding from Cancer Research UK grant PPRCTAGPJT\100003. SH is funded by the Medical Research Council grant MC_UU_00022/1, the Chief Scientist Office of the Scottish Government Health Directorates grant SPHSU17, and Cancer Research UK grant PPRCTAGPJT\100003.

Role of the funding source: The funders of this study had no role in the study design, data analysis, data interpretation, or writing of the report. The corresponding author had full access to all of the data and final responsibility for the decision to submit it for publication.

Acknowledgements: Patients and or public were not involved.

REFERENCES

1. Hammond D, Wackowski OA, Reid JL, O'Connor RJ. Use of JUUL E-cigarettes Among Youth in the United States. Nicotine Tob Res. 2020;22(5):827-32.
2. Duke JC, Allen JA, Eggers ME, Nonnemaker J, Farrelly MC. Exploring Differences in Youth Perceptions of the Effectiveness of Electronic Cigarette Television Advertisements. Nicotine Tob Res. 2016;18(5):1382-86.

For peer review only

3. Farrelly MC, Duke JC, Crankshaw EC, Eggers ME, Lee YO, Nonnemaker JM, et al. A Randomized Trial of the Effect of E-cigarette TV Advertisements on Intentions to Use E-cigarettes. Am J Prev Med. 2015;49(5):686-93.
4. Hammond D, Reid JL, Burkhalter R, Rynard VL. E-cigarette Marketing Regulations and Youth Vaping: Cross-Sectional Surveys, 2017–2019. 2020;146(1).
5. European Commission. Tobacco Products Directive (2014/40/EU). European Commission; 2014. p. 1-38.
6. The Tobacco and Related Products Regulations 2016, 507. Sect. Part 2 (2016).
7. Stead M, Ford A, Angus K, MacKintosh AM, Purves R, Mitchell D. E-Cigarette Advertising in the UK: A Content Analysis of Traditional and Social Media Advertising to Observe Compliance with Current Regulations. Nicotine Tob Res. 2021;23(11):1839-47.
8. Advertising Standards Authority. About the ASA and CAP 2021 [Available from: https://www.asa.org.uk/about-asa-and-cap/about-regulation/about-the-asa-and-cap.html.
9. Advertising Standards Authority. The CAP Code: The UK Code of Non-broadcast Advertising and Direct & Promotional Marketing. London, UK: Advertising Standards Authority; 2016.
10. Advertising Standards Authority. Electronic Cigarettes: Factual vs. Promotional Claims 2018 [Available from: https://www.asa.org.uk/advice-online/electronic-cigarettes-factual-vs-promotional-claims.html.
11. O'Brien EK, Hoffman L, Navarro MA, Ganz O. Social media use by leading US e-cigarette, cigarette, smokeless tobacco, cigar and hookah brands. Tob Control. 2020;29(e1):e87-e97.
12. De Veirman M, Hudders L, Nelson MR. What Is Influencer Marketing and How Does It Target Children? A Review and Direction for Future Research. Front Psychol. 2019;10.
13. Zhu S, Sun J, Binnevie E, Cummins S, Gamst A, Yin L, et al. Four hundred and sixty brands of e-cigarettes and counting: implications for product regulation. Tob Control. 2014;23(3):3-9.
14. Chu KH, Colditz JB, Primack BA, Shensa A, Allem JP, Miller E, et al. JUUL: Spreading Online and Offline. J Adolesc Health. 2018;63(5):582-86.
15. Phua J, Jin SV, Hahm JM. Celebrity-endorsed e-cigarette brand Instagram advertisements: Effects on young adults' attitudes towards e-cigarettes and smoking intentions. J Health Psychol. 2017;23(4):550-60.
16. Amin S, Dunn AG, Laranjo L. Social Influence in the Uptake and Use of Electronic Cigarettes: A Systematic Review. Am J Prev Med. 2020;58(1):129-41.
17. de Andrade M, Hastings G, Angus K. Promotion of electronic cigarettes: tobacco marketing reinvented? BMJ. 2013;DOI: 10.1136/bmj.f7473.
18. Fairchild AL, Bayer R, Colgrove J. The Renormalization of Smoking? E-Cigarettes and the Tobacco "Endgame". NEJM. 2013;370(4):293-95.
19. Schneider S, Diehl K. Vaping as a Catalyst for Smoking? An Initial Model on the Initiation of Electronic Cigarette Use and the Transition to Tobacco Smoking Among Adolescents. Nicotine Tob Res. 2016;18(5):647-53.
20. Etter J-F. Gateway effects and electronic cigarettes. Addiction. 2017;113(10):1776-83.
21. Cobb NK, Brookover J, Cobb CO. Forensic analysis of online marketing for electronic nicotine delivery systems. Tob Control. 2015;24(2):128.
22. Amin S, Dunn AG, Laranjo L. Exposure to e-cigarette information and advertising in social media and e-cigarette use in Australia: A mixed methods study. 2020;213:108112.
23. Prochaska JJ, Pechmann C, Kim R, Leonhardt JM. Twitter=quitter? An analysis of Twitter quit smoking social networks. Tob Control. 2012;21(4):447-49.
24. Chen Y, Tilden C, Vernberg DK. Adolescents' interpretations of e-cigarette advertising and their engagement with e-cigarette information: results from five focus groups. Psychol Health. 2020;35(2):163-76.
25. Scheffels J, Tokle R, Linnansaari A, Rasmussen SKB, Pisinger C. E-cigarette use in global digital youth culture. A qualitative study of the social practices and meaning of vaping among 15-20-year-olds in Denmark, Finland, and Norway. 2023;111:103928.
26. Johnson AC, Mays D, Hawkins KB, Denzel M, Tercyak KP. A Qualitative Study of Adolescent Perceptions of Electronic Cigarettes and Their Marketing: Implications for Prevention and Policy. Child Health Care. 2017;46(4):379-92.

For peer review only - http://bmjopen.bmj.com/site/about/guidelines.xhtml

27. Liu J, McLaughlin S, Lazaro A, Halpern-Felsher B. What Does It Meme? A Qualitative Analysis of Adolescents' Perceptions of Tobacco and Marijuana Messaging. Public Health Rep. 2020;135(5):578-86.
28. Pepper JK, McRee AL, Gilkey MB. Healthcare providers' beliefs and attitudes about electronic cigarettes and preventive counseling for adolescent patients. 2014;54(6):678-83.
29. Guest G, Bunce A, Johnson L. How Many Interviews Are Enough?:An Experiment with Data Saturation and Variability. 2006;18(1):59-82.
30. Smith MJ, Buckton C, Patterson C, Hilton S. User-generated content and influencer marketing involving e-cigarettes on social media: a scoping review and content analysis of YouTube and Instagram. BMC Public Health. 2023;23(1):530.
31. Braun V, Clarke V. Thematic analysis. APA handbook of research methods in psychology, Vol 2: Research designs: Quantitative, qualitative, neuropsychological, and biological. APA handbooks in psychology®.DOI: 10.1037/13620-004. Washington, D.C., USA: American Psychological Association; 2012. p. 57-71.
32. Scottish Government. The Scottish Index of Multiple Deprivation 2020 2020 [Available from: https://www.gov.scot/collections/scottish-index-of-multiple-deprivation-2020/.
33. Ge J. Emoji Sequence Use in Enacting Personal Identity. In: Liu L, White R, editors. WWW '19: Companion Proceedings of The 2019 World Wide Web Conference; New York, USA2019. p. 426-38.
34. Ge J, Gretzel U. Emoji rhetoric: a social media influencer perspective. 2018;34(15-16):1272-95.
35. Jacobson J, Hodson J, Mittelman R. Pup-ularity contest: The advertising practices of popular animal influencers on Instagram. 2022;174:121226.
36. Kostygina G, Tran H, Czaplicki L, Perks SN, Vallone D, Emery SL, et al. Developing a theoretical marketing framework to analyse JUUL and compatible e-cigarette product promotion on Instagram. Tob Control. 2022;10.1136/tobaccocontrol-2021-057120:tobaccocontrol-2021-057120.
37. Alpert JM, Jaisle A, Chen H. A content analysis of the promotional strategies employed by e-cigarette brands on Twitter. 2019;36(4):307-21.
38. Struik LL, Dow-Fleisner S, Belliveau M, Thompson D, Janke R. Tactics for Drawing Youth to Vaping: Content Analysis of Electronic Cigarette Advertisements. 2020;22(8):e18943.
39. Malik A, Li Y, Karbasian H, Hamari J, Johri A. Live, Love, Juul: User and Content Analysis of Twitter Posts about Juul. Am J Health Behav. 2019;43:326-36.
40. Allem JP, Dharmapuri L, Unger JB, Cruz TB. Characterizing JUUL-related posts on Twitter. Drug Alcohol Depend. 2018;190:1-5.
41. Cole-Lewis H, Pugatch J, Sanders A, Varghese A, Posada S, Yun C, et al. Social Listening: A Content Analysis of E-Cigarette Discussions on Twitter. J Med Internet Res. 2015;17(10):e243.
42. Ketonen V, Malik A. Characterizing vaping posts on Instagram by using unsupervised machine learning. Int J Med Inform. 2020;10.1016/j.ijmedinf.2020.104223.
43. Smith MJ, Buckton C, Patterson C, Hilton S. A scoping review and content analysis of YouTube and Instagram posts. In press.
44. de Andrade M, Angus K, Hastings G. Teenage perceptions of electronic cigarettes in Scottish tobacco-education school interventions: co-production and innovative engagement through a pop-up radio project. 2016;136(5):288-93.
45. Choi K, Fabian L, Mottey N, Corbett A, Forster J. Young adults' favorable perceptions of snus, dissolvable tobacco products, and electronic cigarettes: findings from a focus group study. Am J Public Health. 2012;102(11):2088-93.
46. Park E, Kwon M, Gaughan MR, Livingston JA, Chang Y-P. Listening to Adolescents: Their Perceptions and Information Sources About E-cigarettes. 2019;48:82-91.
47. Vogel EA, Ramo DE, Rubinstein ML, Delucchi KL, Darrow SM, Costello C, et al. Effects of Social Media on Adolescents' Willingness and Intention to Use E-Cigarettes: An Experimental Investigation. Nicotine Tob Res. 2020;10.1093/ntr/ntaa003.
48. Vassey J, Valente T, Barker J, Stanton C, Li D, Laestadius L, et al. E-cigarette brands and social media influencers on Instagram: a social network analysis. Tob Control. 2022;10.1136/tobaccocontrol-2021-057053:tobaccocontrol-2021-057053.
49. Dewhirst T. Co-optation of harm reduction by Big Tobacco. Tob Control. 2021;30(e1):e1.

For peer review only

50. Prakash S, Xu Y, Goldenson NI, Wissmann R, Gougelet R, Shiffman S. Transitions in Smoking among Adults Newly Purchasing the JUUL System. Am J Health Behav. 2021;45(3):546-62.
51. Wayne GF, Connolly GN. How cigarette design can affect youth initiation into smoking: Camel cigarettes 1983-93. Tob Control. 2002;11 Suppl 1(Suppl 1):I32-9.
52. Carpenter CM, Wayne GF, Connolly GN. Designing cigarettes for women: new findings from the tobacco industry documents. Addiction. 2005;100(6):837-51.
53. Anderson SJ, Glantz SA, Ling PM. Emotions for sale: cigarette advertising and women's psychosocial needs. Tob Control. 2005;14(2):127-35.
54. Campaign for Tobacco-Free Kids. Campaign for Tobacco-Free Kids. 2013. [cited 2022]. Available from: https://www.tobaccofreekids.org/blog/2013_10_02_ecigarettes.
55. Haugh M. University of Richmond. 2020. [cited 2022]. Available from: https://jolt.richmond.edu/2020/11/27/e-cigarettes-new-technology-but-old-advertising-strategy/.
56. Walley SC, Wilson KM, Winickoff JP, Groner J. A Public Health Crisis: Electronic Cigarettes, Vape, and JUUL. 2019;143(6).
57. Kong G, Morean ME, Cavallo DA, Camenga DR, Krishnan-Sarin S. Reasons for Electronic Cigarette Experimentation and Discontinuation Among Adolescents and Young Adults. Nicotine Tob Res. 2015;17(7):847-54.
58. Audrain-McGovern J, Strasser AA, Wileyto EP. The impact of flavoring on the rewarding and reinforcing value of e-cigarettes with nicotine among young adult smokers. Drug Alcohol Depend. 2016;166(1):263-67.
59. Czoli CD, Goniewicz M, Islam T, Kotnowski K, Hammond D. Consumer preferences for electronic cigarettes: results from a discrete choice experiment. Tob Control. 2016;25(1):30-36.
60. Zare S, Nemati M, Zheng Y. A systematic review of consumer preference for e-cigarette attributes: Flavor, nicotine strength, and type. 2018;13(3):e0194145.
61. Kalaji MNH. The Visual Is Worth Thousands of Words: Adult and Youth Perceptions of Visual Cues in e-Cigarette Advertising [Ph.D.]: Cornell University; 2022.
62. Okawa S, Tabuchi T, Miyashiro I. Who Uses E-cigarettes and Why? E-cigarette Use among Older Adolescents and Young Adults in Japan: JASTIS Study. 2020;52(1):37-45.
63. Cranwell J, Murray R, Lewis S, Leonardi-Bee J, Dockrell M, Britton J. Adolescents' exposure to tobacco and alcohol content in YouTube music videos. Addiction. 2015;110(4):703-11.
64. Williams RS, Derrick J, Ribisl KM. Electronic Cigarette Sales to Minors via the Internet. 2015;169(3):e1563-e63.
65. Schiff S, Liu F, Cruz TB, Unger JB, Cwalina S, Leventhal A, et al. E-cigarette and cigarette purchasing among young adults before and after implementation of California's tobacco 21 policy. Tob Control. 2021;30(2):206.
66. Pike JR, Tan N, Miller S, Cappelli C, Xie B, Stacy AW. The Effect of E-cigarette Commercials on Youth Smoking: A Prospective Study. 2019;43(6):1103-18.
67. Truth Initiative. Old tactics, new products: how big tobacco targets women in e-cigarette marketing 2023 [Available from: https://truthinitiative.org/research-resources/targeted-communities/old-tactics-new-products-how-big-tobacco-targets-women.
68. Statista. Social media sites or apps used by children in the United Kingdom (UK) in 2020 2020 [Available from: https://www.statista.com/statistics/415138/social-media-usage-among-children-uk/#:~:text=Youth%20accounts%20on%20the%20rise,2020%2C%20peaking%20at%2087%20percent.
69. Statista. Reach of leading social networking sites used by those aged 15 to 25 in the United Kingdom (UK) as of 3rd quarter 2020 2022 [Available from: https://www.statista.com/statistics/1059462/social-media-usage-uk-age/.
70. Depue JB, Southwell BG, Betzner AE, Walsh BM. Encoded exposure to tobacco use in social media predicts subsequent smoking behavior. Am J Health Promot. 2015;29(4):259-61.
71. Hébert ET, Case KR, Kelder SH, Delk J, Perry CL, Harrell MB. Exposure and Engagement With Tobacco- and E-Cigarette-Related Social Media. J Adolesc Health. 2017;61(3):371-77.

For peer review only - http://bmjopen.bmj.com/site/about/guidelines.xhtml

Appendix A: Interview topic guide

During the focus groups images were shown in a PowerPoint presentation. A series of images (e.g., Slides 2-8) were shown and changed at five-second intervals and once all images in that section were shown the group were then asked to discuss these based upon questions in the topic guide.

Part 1: Introduction/background

- First of all, thank you for taking part in this research.
- I'm [researcher name], a research assistant at the University of Glasgow with an interest in tobacco and e-cigarettes.

Key points for consent:

- Can I confirm that you have received the participant information sheet and signed and returned your consent form.
- Just to reiterate, your taking part is voluntary, and you are free to withdraw at any time without giving a reason.
- Your participation will be anonymous. Excerpts from the interview may be quoted verbatim in a report and a paper, but quotations will be anonymised.
- Non-anonymised interview recordings and transcripts will be destroyed securely upon completion of this research, but anonymised transcripts and consent forms will be stored securely by the University of Glasgow for a period of 10 years for the purposes of ensuring research integrity.
- Finally, the study has been approved by the Ethics Committee of the College of Social Sciences at the University of Glasgow.
- Reiterate that I am interested to hear their thoughts and experiences as things on social media are changing so quickly and they will know more than me.

Young people's use of social media

First of all, I am going to ask you about social media and what you use.

Show Slide 1 of the PowerPoint presentation.

For peer review only - http://bmjopen.bmj.com/site/about/guidelines.xhtml

1. Do you recognise any of these logos?
    a. PROMPT: Can you tell me what each logo is?
    b. PROMPT: What do you know about each one?
2. Can you tell me about the social media platforms that you use?
    a. Why do you use those?
    b. In what way do you use those?
        i. PROMPT: How do you use them? E.g., ‘liking’, commenting, sharing?
        ii. PROMPT: What is the purpose of each?
        iii. PROMPT: Whether all are the same in the sense of what you can do on them or whether some are different?
    c. What social media platforms do you like and dislike?
        i. PROMPT: Why?
    d. On a daily basis, how long do you tend to spend on social media?
3. What do you think the term ‘social media influencer’ means?
    a. What do social influencers do?
    b. How do you know that someone is a social influencer?
    c. Can you tell me of any social media influencers whom you like and dislike?
    d. What products have you seen influencers advertising?
        i. PROMPT: Clothing, makeup?
    e. How do you think they pick the products that they advertise?
    f. Have you ever bought or used products based on what you have seen on social media?
        i. PROMPT: What type of products were those?
    g. Would you ever like, comment on or share an influencer’s image if they were advertising something?

<u>E-cigarettes on social media</u>

Now I am going to show a few slides on PowerPoint and after we have been through them, we will chat through what you have seen.

For peer review only

Show Slides 2-8 of the PowerPoint presentation.

Eight images of social media influencers and user-generated e-cigarette content were shown to participants. We have not included the images used as this would be considered a copyright and ethics issue.

1. What can you remember from the images that you just saw?
2. Do any of these images grab your attention?
    a. PROMPT: Why?
    b. PROMPT: What do you think about the use of colour in the images?
    c. PROMPT: What do you think about the number of likes each picture has?
    d. PROMPT: What do you think about the caption under the images?
3. Did the images have anything in common?
    a. If so, what?
4. Did you recognise anyone in the images?
    a. If so, who?
5. Why do you think these images have been posted?
6. What do you think is the purpose of the images?
7. What do you think the image is trying to say?
    a. PROMPT: How do you know this?
8. Who do you think these images are targeted at?
    a. PROMPT: Why do you say that?
9. Do you think these images are selling a product?
    a. PROMPT: How can you tell if a product is being promoted or not?
    b. PROMPT: How do you know which product(s) are being promoted?
10. Who do you think has posted the images I showed you?
    a. Do you think that person is being paid to post the image or not?
    b. How can you tell if someone is being paid or not?
    c. Why do you think influencers would want to advertise e-cigarette products?
    d. Why do you think companies would pay influencers to advertise their products?

Show slides 9-12 of the PowerPoint presentation.

For peer review only - http://bmjopen.bmj.com/site/about/guidelines.xhtml

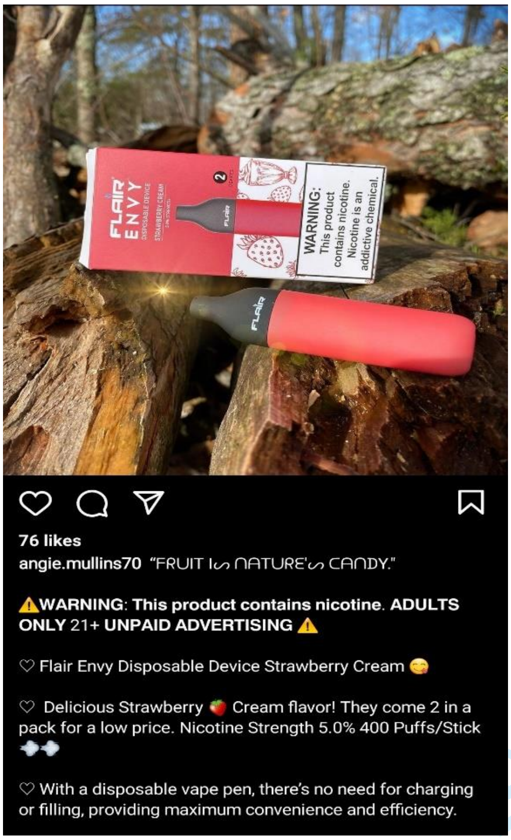

Slide 9

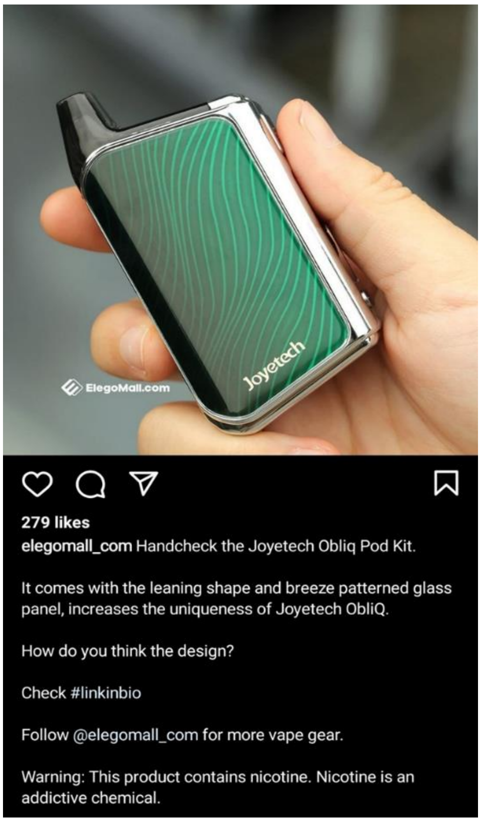

Slide 10

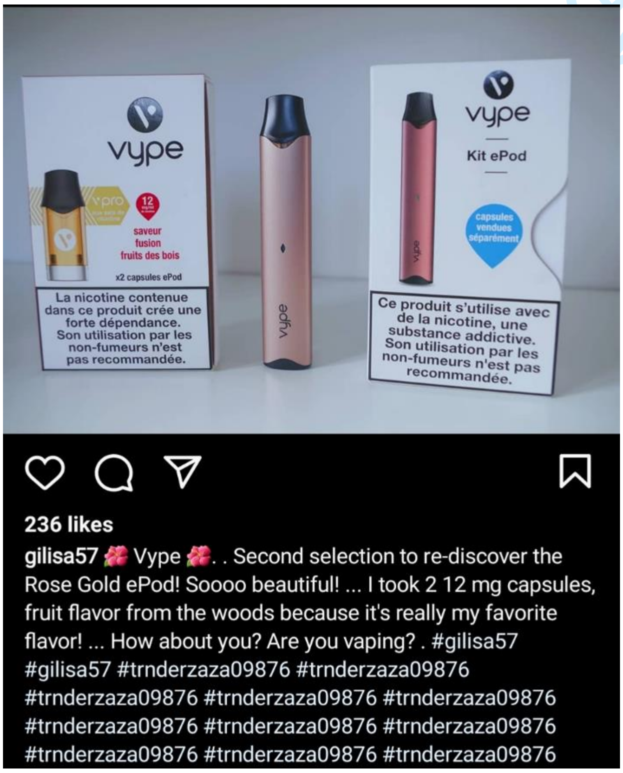

Slide 11

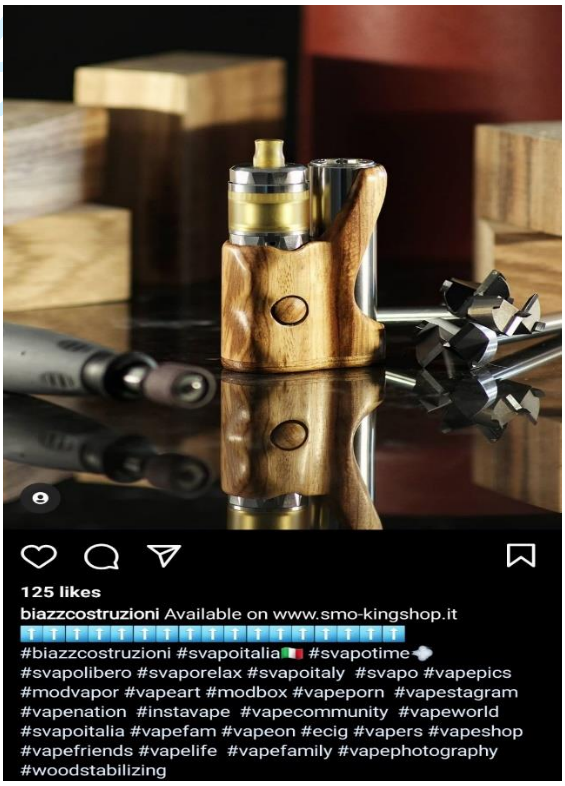

Slide 12

4

1. What can you remember from the images that you just saw?
2. Do any of these images grab your attention?
    a. PROMPT: Why?
    b. PROMPT: What do you think about the use of colour in the images?
    c. PROMPT: What do you think about the number of likes each picture has?
    d. PROMPT: What do you think about the caption under the images?
3. Did the images have anything in common?
    a. If so, what?
4. Do any of these images remind you of any other products?
    a. PROMPT: Why?
5. Why do you think these images have been posted?
6. What do you think is the purpose of the images?
7. What do you think the image is trying to say?
    a. PROMPT: How do you know this?
8. Who do you think these images are targeted at?
    a. PROMPT: Why do you say that?
9. Why do you think e-cigarettes have been designed this way?

Show slides 14-18 of the PowerPoint presentation.

For peer review only

For peer review only - http://bmjopen.bmj.com/site/about/guidelines.xhtml

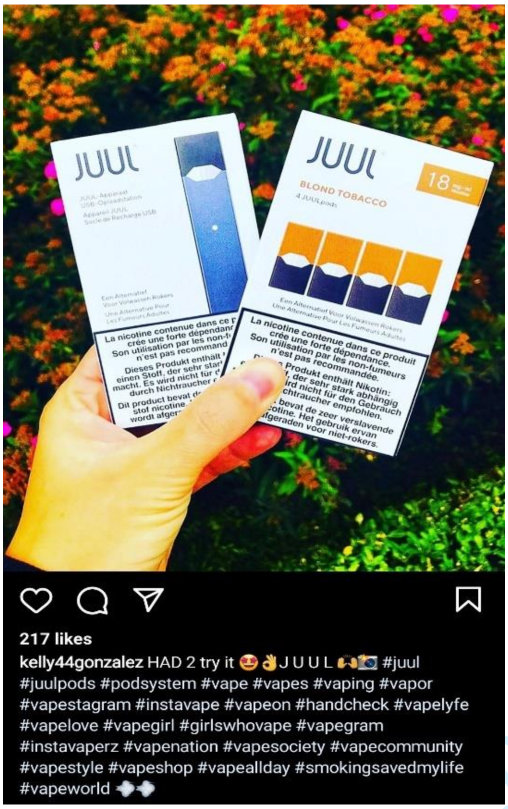

Slide 14

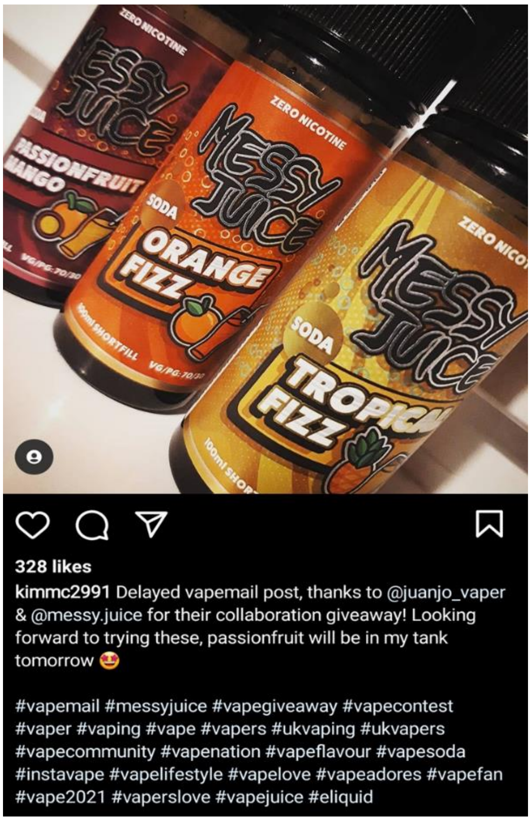

Slide 15

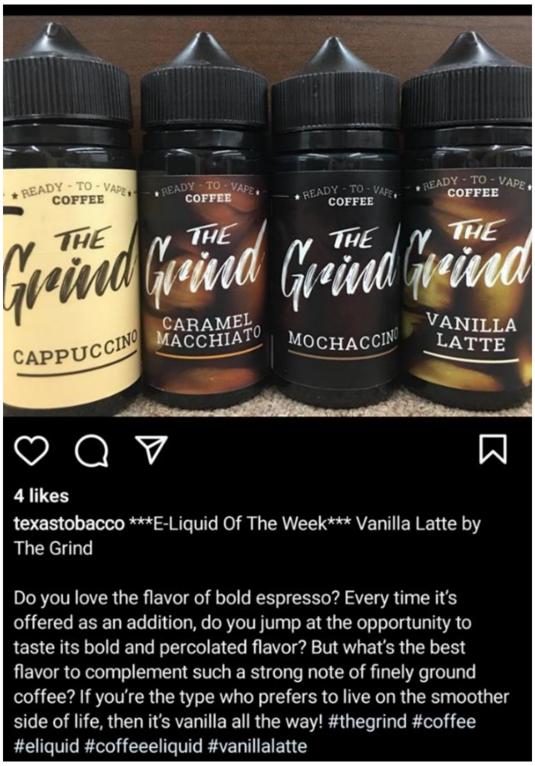

Slide 16

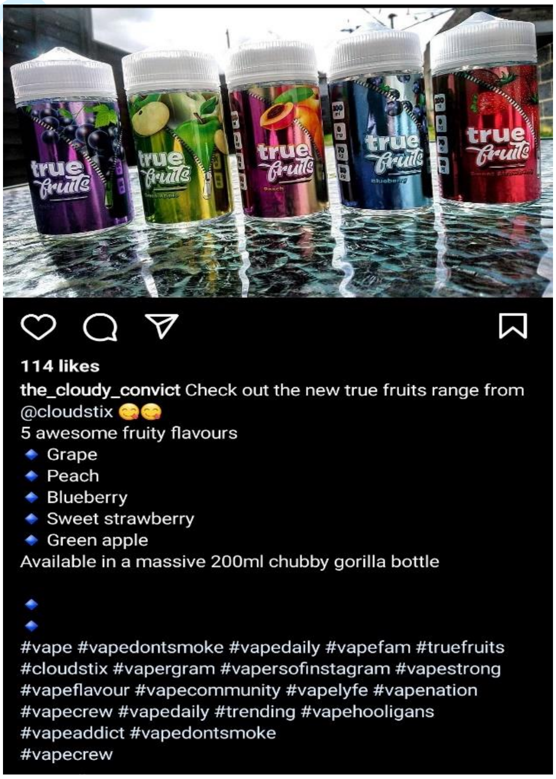

Slide 17

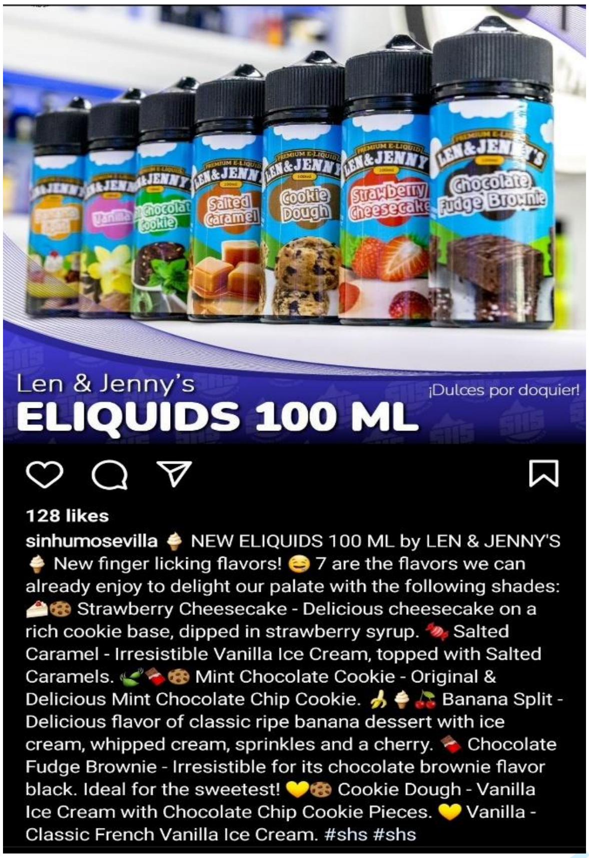

Slide 18

review only

1. What can you remember from the images that you just saw?
2. Do any of these images grab your attention?
    a. PROMPT: Why?
    b. PROMPT: What do you think about the colour of the products?
    c. PROMPT: What do you think about the number of likes each picture has?
    d. PROMPT: What do you think about the caption under the images?
3. Did the images have anything in common?
    i. If so, what?
4. Do any of these images remind you of any other products?
    a. PROMPT: Why?
5. Do you think these images are selling a product?
    a. Do you know what the products are that are shown in the images?
        i. If yes, how do you know that?
6. Why do you think these images have been posted?
7. What do you think is the purpose of the images?
8. What do you think the image is trying to say?
    a. PROMPT: How do you know this?
9. Who do you think these images are targeted at?
    a. PROMPT: Why do you say that?
10. What do you think of the flavours shown?
11. Do you see some flavours as more appealing compared to others?
    a. PROMPT: What ones and why?
12. Do you think there are any flavours that are more appealing to youths compared to adults?
    a. PROMPT: What ones and why?

Personal experiences

I am now moving on to ask about your own experiences and of friends and other people.

1. Have you seen any information about e-cigarettes on social media platforms?
    a. What kind of information was it?
        i. PROMPT: Why do you think the images were posted?
        ii. PROMPT: What do you think the images were trying to show/tell you?
2. Do friends or anyone you follow on social media post or share messages about e-cigarettes?
    a. How does that make you feel about them?
    b. Why do you think they post or share these messages?
    c. If seeing a friend or someone you know post about e-cigarettes on social media, would it make you want to share images or messages?

For peer review only - http://bmjopen.bmj.com/site/about/guidelines.xhtml

d. Out of interest have you or your friends ever posted on social media about e-cigarettes?

Warnings on social media posts

1. Play the video (short clip) https://www.youtube.com/watch?v=tk9SQDH_ZNo
    a. Did you notice anything about the short clip I just showed you?
    b. Can you describe what you remember from the clip I just showed you?
    c. IF NECESSARY PROMPT: Did anyone notice the warning notice?
    d. PROMPT: What did it say?
    e. PROMPT: What did you think about it?
2. Replay the video pointing out the warning.
    a. What do you think about the length of time that the warning was shown for?
        i. How easy or difficult was it to notice the warning?
3. Why do you think there are warnings on posts/videos that show e-cigarette products?
4. Do you think there should or should not be warnings on posts/videos showing e-cigarette products?
    a. PROMPT: Why?

Final remarks

1. Have you ever noticed the marketing/promotion of e-cigarette products before or not?
2. How do you think about the marketing/promotion of e-cigarette products on social media?

Close interview

- Is there anything else you would like to add, that we haven't already talked about?
- Thank you very much for taking part.

For peer review only

Standards for Reporting Qualitative Research (SRQR)*

http://www.equator-network.org/reporting-guidelines/srqr/

Title and abstract

	Page/line no(s).
**Title** - Concise description of the nature and topic of the study Identifying the study as qualitative or indicating the approach (e.g., ethnography, grounded theory) or data collection methods (e.g., interview, focus group) is recommended	1
**Abstract** - Summary of key elements of the study using the abstract format of the intended publication; typically includes background, purpose, methods, results, and conclusions	2

Introduction

**Problem formulation** - Description and significance of the problem/phenomenon studied; review of relevant theory and empirical work; problem statement	4 and 5
**Purpose or research question** - Purpose of the study and specific objectives or questions	5

Methods

**Qualitative approach and research paradigm** - Qualitative approach (e.g., ethnography, grounded theory, case study, phenomenology, narrative research) and guiding theory if appropriate; identifying the research paradigm (e.g., postpositivist, constructivist/ interpretivist) is also recommended; rationale**	6 and 7
**Researcher characteristics and reflexivity** - Researchers' characteristics that may influence the research, including personal attributes, qualifications/experience, relationship with participants, assumptions, and/or presuppositions; potential or actual interaction between researchers' characteristics and the research questions, approach, methods, results, and/or transferability	7, 18 and 19
**Context** - Setting/site and salient contextual factors; rationale**	6 and 7
**Sampling strategy** - How and why research participants, documents, or events were selected; criteria for deciding when no further sampling was necessary (e.g., sampling saturation); rationale**	6 and 7
**Ethical issues pertaining to human subjects** - Documentation of approval by an appropriate ethics review board and participant consent, or explanation for lack thereof; other confidentiality and data security issues	7
**Data collection methods** - Types of data collected; details of data collection procedures including (as appropriate) start and stop dates of data collection and analysis, iterative process, triangulation of sources/methods, and modification of procedures in response to evolving study findings; rationale**	6 and 7
**Data collection instruments and technologies** - Description of instruments (e.g., interview guides, questionnaires) and devices (e.g., audio recorders) used for data collection; if/how the instrument(s) changed over the course of the study	6 and 7
**Units of study** - Number and relevant characteristics of participants, documents, or events included in the study; level of participation (could be reported in results)	6 and 7

For peer review only

**Data processing -** Methods for processing data prior to and during analysis, including transcription, data entry, data management and security, verification of data integrity, data coding, and anonymization/de-identification of excerpts	6 and 7
**Data analysis -** Process by which inferences, themes, etc., were identified and developed, including the researchers involved in data analysis; usually references a specific paradigm or approach; rationale**	6 and 7
**Techniques to enhance trustworthiness -** Techniques to enhance trustworthiness and credibility of data analysis (e.g., member checking, audit trail, triangulation); rationale**	6 and 7

Results/findings

**Synthesis and interpretation -** Main findings (e.g., interpretations, inferences, and themes); might include development of a theory or model, or integration with prior research or theory	8-15
**Links to empirical data -** Evidence (e.g., quotes, field notes, text excerpts, photographs) to substantiate analytic findings	8-15

Discussion

**Integration with prior work, implications, transferability, and contribution(s) to the field -** Short summary of main findings; explanation of how findings and conclusions connect to, support, elaborate on, or challenge conclusions of earlier scholarship; discussion of scope of application/generalizability; identification of unique contribution(s) to scholarship in a discipline or field	15-19
**Limitations -** Trustworthiness and limitations of findings	18 and 19

Other

**Conflicts of interest -** Potential sources of influence or perceived influence on study conduct and conclusions; how these were managed	20
**Funding -** Sources of funding and other support; role of funders in data collection, interpretation, and reporting	20

*The authors created the SRQR by searching the literature to identify guidelines, reporting standards, and critical appraisal criteria for qualitative research; reviewing the reference lists of retrieved sources; and contacting experts to gain feedback. The SRQR aims to improve the transparency of all aspects of qualitative research by providing clear standards for reporting qualitative research.

**The rationale should briefly discuss the justification for choosing that theory, approach, method, or technique rather than other options available, the assumptions and limitations implicit in those choices, and how those choices influence study conclusions and transferability. As appropriate, the rationale for several items might be discussed together.

Reference:
O'Brien BC, Harris IB, Beckman TJ, Reed DA, Cook DA. **Standards for reporting qualitative research: a synthesis of recommendations.** *Academic Medicine*, Vol. 89, No. 9 / Sept 2014
DOI: 10.1097/ACM.0000000000000388

For peer review only

For peer review only

For peer review only - http://bmjopen.bmj.com/site/about/guidelines.xhtml

BMJ Open

Youth's exposure to and engagement with e-cigarette marketing on social media: a UK focus group study

Journal:	*BMJ Open*
Manuscript ID	bmjopen-2022-071270.R3
Article Type:	Original research
Date Submitted by the Author:	20-Jun-2023
Complete List of Authors:	Smith, Marissa; University of Glasgow, MRC/CSO Social and Public Health Sciences Unit<br>Hilton , Shona; University of Glasgow, MRC/CSO Social and Public Health Sciences Unit
**Primary Subject Heading**:	Public health
Secondary Subject Heading:	Qualitative research
Keywords:	PUBLIC HEALTH, QUALITATIVE RESEARCH, Health policy < HEALTH SERVICES ADMINISTRATION & MANAGEMENT

SCHOLARONE™
Manuscripts

I, the Submitting Author has the right to grant and does grant on behalf of all authors of the Work (as defined in the below author licence), an exclusive licence and/or a non-exclusive licence for contributions from authors who are: i) UK Crown employees; ii) where BMJ has agreed a CC-BY licence shall apply, and/or iii) in accordance with the terms applicable for US Federal Government officers or employees acting as part of their official duties; on a worldwide, perpetual, irrevocable, royalty-free basis to BMJ Publishing Group Ltd ("BMJ") its licensees and where the relevant Journal is co-owned by BMJ to the co-owners of the Journal, to publish the Work in this journal and any other BMJ products and to exploit all rights, as set out in our licence.

The Submitting Author accepts and understands that any supply made under these terms is made by BMJ to the Submitting Author unless you are acting as an employee on behalf of your employer or a postgraduate student of an affiliated institution which is paying any applicable article publishing charge ("APC") for Open Access articles. Where the Submitting Author wishes to make the Work available on an Open Access basis (and intends to pay the relevant APC), the terms of reuse of such Open Access shall be governed by a Creative Commons licence – details of these licences and which Creative Commons licence will apply to this Work are set out in our licence referred to above.

Other than as permitted in any relevant BMJ Author's Self Archiving Policies, I confirm this Work has not been accepted for publication elsewhere, is not being considered for publication elsewhere and does not duplicate material already published. I confirm all authors consent to publication of this Work and authorise the granting of this licence.

For peer review only

Youth's exposure to and engagement with e-cigarette marketing on social media: a UK focus group study

Marissa J. Smith[1*] and Shona Hilton[1]

[1] MRC/CSO Social and Public Health Sciences Unit, University of Glasgow, Glasgow, G3 7HR, UK

*Corresponding author

Email: marissa.smith@glasgow.ac.uk Tel: (+44) 0141 353 7500

Keywords: Health policy, qualitative research, public health

For peer review only

ABSTRACT

Objective: E-cigarettes are promoted creatively through social media and considering the potential influence of social media marketing on young people, we explored young people's exposure to and engagement with social media marketing of e-cigarettes.

Design: Semi-structured discussion groups.

Subjects: Twenty focus groups with 82 young people aged 11-16 living in the Central belt of Scotland.

Methods: Youths were asked about smoking and vaping behaviours, social media use, vaping advertisement exposure, and were shown illustrative examples of social media content (e.g., images and videos) about different messages, presentations, and contextual features. Transcripts were imported into NVivo 12, coded thematically and analysed.

Results: Youths highlighted a variety of tactics e-cigarette companies use, including influencer or celebrity endorsement, attractive youth flavours, bright colours and emotional appeal to advertise and promote their products directly to young people. Social media influencers who advertise e-cigarettes were described as portraying e-cigarettes as 'cool' and 'fashionable' to entice viewers to try the products. Youths considered that there is a need for more restrictions on social media content to protect youths while also still allowing smokers to purchase them as a cessation device.

Conclusions: Our study highlights that the e-cigarette industry is using previously employed tactics similar to the tobacco industry to advertise and promote its products on social media. These findings suggest the growing need for governments to work together to develop and implement policies to restrict the advertising and marketing of e-cigarettes on social media.

For peer review only - http://bmjopen.bmj.com/site/about/guidelines.xhtml

Strengths and limitations of this study

- This study is one of the first in-depth studies to explore youths' responses to and engagement with e-cigarette marketing on social media with a focus on the rise in user-generated and influencer content.
- This research is very timely as governments consider the need for further e-cigarette regulations.
- A methodological strength is the rich dataset from semi-structured interviews with 82 youths aged 11-16, facilitating understandings of youths' exposure to and interaction with e-cigarette marketing on social media.
- Qualitative thematic analysis of the data allows depth of opinions but cannot offer predictions about the frequency of specific opinions with a wider population.

For peer review only

INTRODUCTION

Marketing strategies used by electronic cigarette (e-cigarette) companies have been associated with the uptick in e-cigarette use (vaping) among youth [1]. Many studies have found that the promotion of e-cigarettes through various channels (e.g., television (TV), social media influencers) has lent to increased positive perceptions of vaping and intentions to use vaping products and contributed to e-cigarette uptake among youth [2-4].

In the UK, the advertising of e-cigarettes is regulated through Article 20(5) of the revised European Union (EU) Tobacco Products Directive (2014/40/EU) (TPD), [5] which was transposed into UK law by the Tobacco and Related Products Regulations (TRPR) 2016 [6]. The TPD prohibited the advertising of nicotine-containing e-cigarettes (unless licensed as medicines) in channels with potential cross-border impact (i.e. channels that show adverts or sponsored events that originate from non-EU countries in EU countries), including TV, radio, newspapers, magazines, and sponsorship [7]. Online advertising was also prohibited under the TPD, although the regulations left scope for marketers to retain websites containing factual information about e-cigarette products. As there are currently no medicinally licensed nicotine vaping products in the UK, the prohibitions apply to all nicotine-containing e-cigarette products on the market. The TRPR requirements for e-cigarette advertising were set out in 2017 and are enforced by the Committee of Advertising Practice (CAP) – a self-regulatory body of organisations representing advertising, direct marketing, media businesses, and sales promotion endorsed and administered by the independent Advertising Standards Authority (ASA) [8, 9]. Although Rule 22.12 of the ASA CAP code prohibits advertising in online media, social media content for e-cigarettes is permitted in "non-paid-for space online under the marketer's control, provided claims are factual rather than promotional" [10]. Promotional content includes descriptive language that goes beyond objective, factual claims, significant imagery that is not related to the product, and health claims (e.g. that e-cigarettes are safer or healthier than tobacco) [10].

Social media marketing includes promotions by means of paid digital advertising, via compensated 'influencers' (individuals with large followings who are paid to advertise brands and products), and on brand pages that companies can create for free and use to share content [11]. Most adolescents use

For peer review only

For peer review only - http://bmjopen.bmj.com/site/about/guidelines.xhtml

visual-based social media daily. User-generated and influencer marketing content on social media represents a key influence on young people's understanding of products, including e-cigarettes [12]. E-cigarettes are promoted creatively through social media, with well-designed features including colours, flavour variations, emotional appeal, incentives (such as price promotions and discount vouchers), and even celebrity endorsements [13, 14]. Research has shown that online marketing that leads to exposure to e-cigarette advertising, including where it is concealed as information or recommendations from peers, can increase the likelihood of vaping in individuals, including among youths [15-17]. In addition, a number of researchers have suggested that e-cigarette advertising could lead to increases in how socially acceptable and desirable cigarettes are perceived to be, and subsequently influence their continued use or possible (re)uptake in smokers, e-cigarette users and dual-users [18-21]. E-cigarette companies have been promoting their products on social media in an attempt to expand their market [22, 23]. In addition, e-cigarette companies use social media platforms to promote e-cigarettes and create a socially attractive vaping image [24]. Previous studies [including 25, 26, 27] have explored youths' perceptions of e-cigarette marketing on social media, finding that social media adverts entice non-smokers to use e-cigarettes, emotional appeals are featured in adverts, use of vibrant colours in the packaging, appeal of flavours and advertisements portray e-cigarettes as less harmful than combustible cigarettes. Liu et al.[28] examined youths; perception of tobacco and marijuana messaging on social media and reported similar findings (including, appealing colours in the packaging and appealing flavours). One limitation of the literature in this area is that it focuses on either smokers, non-smokers and/or vapers. This leaves out non-vapers and dual-users (those who use both cigarettes and e-cigarettes), understudied. It is important to establish whether exposure to e-cigarette adverts on social media influence attitudes towards e-cigarettes in non-vapers and smoking in dual-users.

Gaining a better understanding of the nature, extent, and impact of e-cigarette marketing including their possible effects on youth e-cigarette perceptions and use can help guide the development of policies and interventions to educate youths about e-cigarettes [29]. This study, therefore, aims to explore youths', including non-smokers and non-vapers and dual-users exposure to, and engagement with e-cigarette advertising on social media, including user-generated and influencer content.

For peer review only

For peer review only - http://bmjopen.bmj.com/site/about/guidelines.xhtml

METHODS

This section closely follows the methodological approach detailed in [30]. The dataset has been rigorously analysed to answer two distinct research questions which have been answered in two separate manuscripts. Smith et al. [30] explored youth's perceptions and engagement with e-cigarettes focusing on one type/generation of e-cigarettes; disposables. This manuscript explores young people's exposure to, and engagement with all types/generations of e-cigarettes but focuses on advertising on social media, including user-generated and influencer content.

We conducted 20 focus groups in Scotland between March and May 2022. Focus groups included between three and five participants (a total of 82 participants). Purposive sampling was used to recruit a diverse sample of youths in terms of age, sex, socio-economic background, smoking status, and vaping status [31]. Eleven groups were recruited through youth workers in local youth organisations. These gatekeepers handed out information sheets and helped achieve the sampling frame in terms of youth demographics and experiences concerning combustible cigarettes and e-cigarettes. The three organisations that helped with participant recruitment worked specifically with young people from disadvantaged backgrounds in urban areas. This recruitment strategy resulted in the inclusion of a range of participants from more and less deprived backgrounds and with experiences of smoking and vaping. Seven groups were recruited through the Schools Health and Wellbeing Improvement Research Network (SHINE) Newsletter which is distributed monthly to over 500 schools in Scotland. The remaining two groups were recruited via personal networks directly by MS.

Focus group discussions were facilitated to allow the research team to explore opinions on online advertising and marketing of e-cigarettes. Friendship groups of 3–5 participants were used to facilitate in-depth insights and promote participant interaction. Each participant was given a £20 shopping voucher as compensation for their time.

Prior to the start of the focus groups, participants completed a short anonymous questionnaire about their age, biological sex, postcode, smoking, and e-cigarette use status. For both combustible cigarettes and e-cigarettes, the questionnaire asked participants to specify whether they had tried or

For peer review only

used them in the past or were using them at the time of the study. Based on a scoping review of user-generated content and influencer marketing involving e-cigarettes on social media [14], a topic guide (Appendix A) was developed which covered three key areas, including use of social media, perceptions of social media influencers versus user-generated e-cigarette content on social media, and views on the marketing of e-cigarettes on social media.

Images of different types of e-cigarettes ('tanks', disposables, and pod devices) and e-liquids posted by social media influencers and general users were used as conversation starters found during the scoping review part of this research [14], were used as conversation starters. Group discussions were facilitated by MS (a young post PhD early career researcher post and experienced qualitativeresearcher). Ten of the groups were conducted online using Microsoft Teams and ten of the groups were conducted face-to-face. Of these, one of the groups was conducted on the youth organisation's premises, and the other nine were conducted at the school, with representatives of the youth organisation present. Groups lasted between 40 and 66 minutes. Field-notes reflecting on the focus group and individual issues discussed were written up for each group. All focus groups were audio recorded (with participants' permission) and transcribed verbatim.

Analysis

Following Braun and Clarke's six-phase framework for thematic analysis [32], we conducted thematic analysis of the data form the focus groups. The research team read and re-read the transcripts to become familiar with the data, and then iteratively constructed a coding frame to enable consistent organisation of relevant data. NVivo was used to organise categories on the basis of inductive themes that emerged from close reading of the, capture of both areas of agreement and less typical perspectives across a range of categories. Each transcript was imported into NVivo 12, coded independently, cross-checked, and analysed by MS and SH (professor of public health and experienced qualitative researcher). Contradictory cases and group dynamics were discussed, making use of transcripts and field notes. The researchers reflected on their role as researcher, remained constantly aware of their position and took care not to introduce bias throughout the research. To

For peer review only

further reduce bias the researcher (MS) recorded the focus groups and analysed them some time after they were completed ensuring a more reflective viewpoint of occurrences. Ethical approval for the study was obtained from the University of Glasgow's Medical and Veterinary Life Sciences Ethics Committee (reference 200210034).

Patient and Public Involvement

Patients and/or the public were not involved in the design of the study, developed of the research questions, recruitment, outcome measures or conduct of the study. A summary of the results will be disseminated to study participants.

RESULTS

Participant characteristics

Eighty-two youths aged 11–16 years participated (47 females (57%) and 35 males (43%)), representing diversity in sociodemographic characteristics and smoking-related behaviours. Age distribution within the sample was skewed slightly towards 14–15-year-olds, with 14-year-olds making up the largest subgroup (n= 24). While the majority of participants did not currently smoke or use e-cigarettes, the sample included 10 smokers and 18 youths who used e-cigarettes. Deprivation rank was assigned using the Scottish Index of Multiple Deprivation (SIMD) [33]. The area-level deprivation grouped using a binary deprivation variable (least deprived/most deprived) in which the three most deprived quintiles were grouped into the most deprived category for the Central Belt of Scotland area. Table 1 describes the focus group composition and participants in more detail and Table 2 summarises smoking and e-cigarette use among the sample.

For peer review only

Group	Area-level deprivation	Sex	Age	Cigarette Smoker	E-cigarette use
1	Least deprived	Female	13-15	Never	Never
2	Least deprived	Female	14-15	Never	Mixed – Never (4) / Tried (1)
3	Least deprived	Female	13-16	Never	Mixed – Never (2) / Tried (1)
4	Most deprived	Mixed – Male (3)/Female (2)	12-15	Mixed – Never (3) / Current (2)	Mixed – Never (3) / Tried (1)/ Current (1)
5	Most deprived	Mixed – Male (1)/ Female (4)	14-16	Mixed – Never (2)/ Tried (2)/ Current (1)	Mixed – Never (2)/ Tried (2)/ Current (1)
6	Most deprived	Male	12-15	Never	Never
7	Most deprived	Male	16	Current	Current
8	Least deprived	Mixed – Male (2)/Female (3)	14	Never	Never
9	Most deprived	Male	16	Mixed – Tried (1)/ Current (2)	Current
10	Most deprived	Mixed – Male (4)/Female (1)	14-15	Mixed – Never (3)/ Tried (1)/ Current (1)	Mixed – Never (3)/ Tried (1)/ Current (1)
11	Most deprived	Mixed – Male (3)/Female (2)	13-16	Mixed – Never (2)/ Tried (2)/ Current (1)	Mixed – Never (1)/ Current (4)
12	Least deprived	Mixed – Male (2)/Female (1)	15-16	Tried	Mixed – Tried (2)/ Current (1)
13	Least deprived	Female	13-16	Never	Never
14	Most deprived	Mixed – Male (1)/Female (3)	11-12	Never	Never
15	Most deprived	Mixed – Male (3)/Female (1)	11-12	Never	Never
16	Most deprived	Mixed – Male (2)/Female (2)	11-12	Never	Never
17	Most deprived	Female	14-16	Mixed – Never (4)/ Tried (1)	Mixed – Never (1)/ Tried (1)/ Current (3)
18	Most deprived	Male	13-16	Never	Never
19	Most deprived	Female	14	Never	Mixed – Tried (2)/ Current (1)
20	Least deprived	Female	15-16	Never	Tried (3)

Table 1: Focus group location, participants, and their cigarette smoking and e-cigarette use.

For peer review only

	E-cigarette use											
	Never			Tried			Current			Total		
**Cigarette smoker**	**n**	**(col %)**	**(row %)**	**n**	**(col %)**	**(row %)**	**n**	**(col %)**	**(row %)**	**n**	**(col %)**	**(row %)**
**Never**	51	98.1%	82.3%	7	58.3%	11.3%	4	22.2%	6.5%	62	75.6%	100.0%
**Tried**	1	1.9%	10.0%	4	33.3%	40.0%	5	27.8%	50.0%	10	12.2%	100.0%
**Current**	0	0.0%	0.0%	1	8.3%	10.0%	9	50.0%	90.0%	10	12.2%	100.0%
**Total**	52	1	92.3%	12	100.0%	14.6%	18	100.0%	22.0%	82	100.0%	100.0%

Table 2: E-cigarette use according to cigarette smoking.

For peer review only

Perceptions of advertising tactics used by the e-cigarette industry to target youths

Youths discussed three ways (influencer or celebrity endorsement, flavourings and emotional appeal) e-cigarette companies market their products to target young people.

Influencer or celebrity endorsements

During the focus groups, participants frequently discussed the prevalence of social media influencers advertising e-cigarette products. When discussing why e-cigarette and/or tobacco companies would want to use social media influencers to advertise their products, participants highlighted the popularity of social media and how this would increase the reach of the adverts.

"I guess companies would use them [influencers] because the company may have approached them to advertise the product and they know they might have a huge following on the platform, so they might influence some more people to buy the products." (Male, current smoker, current vaper)

"More people are on social media nowadays. So, it's better that they [vaping/tobacco companies] use influencers to advertise their products compared to say in newspapers, 'cause you don't see people reading newspapers anymore." (Male, never smoker, never vaper)

Participants reported seeing marketing and advertising of e-cigarettes on social media and that they were portrayed as 'cool' and 'fashionable' by social media influencers.

"If an influencer posts it, a lot of people see it and be like, that's the trend now, it's a cool thing to do, so I want to take part in that." (Female, never smoker, tried vaping)

For peer review only - http://bmjopen.bmj.com/site/about/guidelines.xhtml

"It's like they're showcasing them. It's not just like it's in the background or whatever, it's not a normal pose to have them in, it's like they're showcasing them and that they are a fashionable thing to carry or to have." (Female, never smoker, never vaper)

Participants discussed that this would influence the viewer to try the product, thus increasing the prevalence of use.

"People get tempted easily by seeing the post. Like if they saw some influencers trying that stuff [e-cigarettes], people get tempted quickly to try that stuff, because then that person is trying it so the like the kids would think this is something that I should also try that stuff like that, that's the point." (Male, current smoker, current vaper)

"When you see what an influencer is, most people think oh if they're doing that, then it's cool, so it like, it's making more people want to buy the vapes, and stuff." (Female, never smoker, tried vaping)

Several participants discussed how social media posts make it too easy for underage youths to purchase and hide the fact you are buying e-cigarette products from websites.

"I've seen a thing on TikTok like, they [influencers] show you them putting them [e-cigarettes] in the wee boxes and all that, or you could put them in secret packaging like behind the lashes. Like you can order it off their website and they'll hide it in the packaging, they put a few bits of sweeties on top of your vapes so your mum doesn't see it." (Female, tried smoking, current vaper)

Using sweet and fruity flavours to appeal to youths

For peer review only - http://bmjopen.bmj.com/site/about/guidelines.xhtml

Participants discussed the variety of flavours of e-cigarettes available, highlighting that the sweet and fruity flavours are particularly attractive to youths.

"'Cause younger people, like us would think that the flavoured ones look nice so they would try then and then they'd start vaping." (Male, never smoker, never vaper)

"I think the type of ice cream flavours or sweetie ones are targeted at younger people because most people in their 30s and 40s would probably use the tobacco one or the coffee ones." (Male, current smoker, current vaper)

"The ones that stood out to me the most were the Ben and Jerry's and fruit ones compared to the tobacco ones. I feel like, if I was going to buy them, I would buy them 'cause they are attractive and are things I like." (Female, tried smoking, current vaper)

Several participants stated that the variety of flavourings was to appeal to youths.

"I feel like no one really wants to taste tobacco, to be honest. But I think putting out there with like grape and blueberry flavourings are going to attract younger people to the marketplace. I think it's an intentional design and I feel like that is probably what it does attract youths." (Female, never-smoker, never vaper)

Using vibrant colours to attract youth attention

In addition, participants spoke about the presentation of e-cigarette products on social media, for example, the use of vibrant colours and filters.

For peer review only

"You can tell by the picture they're promoting them. Because if it was not promoting them you would have like a red colour, it's like a stop, it's kind of like a no colour. But they are using really positive colours, like pink and green. It makes you stop and look at it to see what it's [the social media post] is about." (Male, never smoker, never vaper)

"It was really bright colours and they had bright filters on them so it is brighter. If you were scrolling past then gives you a bright colour, you'd go back and see it. You'd be like, oh what's that? You'd be, like, drawn to it. [...] I think young people like us would be drawn to that, well I would." (Female, never smoker, never vaper)

Connecting emotionally with youths

Participants discussed the emotional connections and positive portrayal of e-cigarettes on social media, stating that individuals in the social media posts 'looked happy' to be using the products.

"I think the person who posted this product, I think he or she was trying to emphasise the fact that these products are good because they look happy to be advertising it. So, you should try them and like they were not promoting vaping negatively or that it is bad at all. I think they were trying to sell the aesthetic idea of this product." (Male, current smoker, current vaper)

"It's the way they're all holding them, they looked quite passionate with a smile on their face. A big cheesy smile." (Male, current smoker, current vaper)

For peer review only

Participants highlighted the use of emojis in e-cigarette social media posts. Emojis (icons used to express an idea or emotion) and emoji sequencing (the use of two or more emojis that form a conceptual rhetorical unit) [34] are used to enhance the message meaning [35, 36]. When asked if this would affect their interaction with social media posts that included emojis, participants stated that they would be more inclined to read the post, rather than scrolling past.

"If you see it in colours, like emojis, you're like, oh I wonder what that is. You'd stop scrolling. 'Cause normally captions is just, like, text and a black background or something like that." (Female, never smoker, never vaper)

"The wee emojis they would draw my attention, because of how brightly coloured they are. And I think that's what they're trying to do, like, it'll draw your attention." (Female, never smoker, never vaper)

Perceptions of what can be done to limit e-cigarette marketing on social media

Participants had divergent views on the marketing and advertising of e-cigarettes on social media. Several participants stated that marketing and advertising should not be allowed as youths, including those of a similar age, could access this type of content and it may encourage them to try e-cigarettes.

"Advertising them [e-cigarettes] on social media is s a bad thing because younger people might see it and then try it." (Male, never smoker, never vaper)

I don't think it [advertising of e-cigarettes] should be allowed, just because of the younger community on it [social media]. And then if they are seeing them, they might end up doing it, getting addicted and end up dying." (Male, tried smoking, tried vaping)

For peer review only

But participants also acknowledged that controlling what is posted on social media is ‘tricky’ and ‘difficult’ and that prohibiting e-cigarette advertising may limit their role as cessation devices by smokers. Those participants suggested instead of prohibiting the marketing and advertising of e-cigarettes, warnings (such as age and health warnings) or other restrictive measures (such as parental controls or age restrictions) could be imposed on these types of social media posts.

“I think there needs to be age restrictions on the posts or parents could control what their child sees.” (Female, never smoker, never vaper)

DISCUSSION

E-cigarettes have become increasingly popular and visible in public life, particularly on social media. Our qualitative thematic analysis of youths’ perceptions of and engagement with e-cigarette advertising on social media highlighted a public health concern. Similar to previous research [37-39], youths in our study highlighted the tactics used by the e-cigarette industry to promote and advertise e-cigarettes to underage populations through influencer and celebrity endorsements, variety of flavours and using promotional endorsements. Youths discussed the positive portrayal of e-cigarettes by social media influencers, and they were enticing the viewer to buy the product. Youths highlighted that e-cigarette and/or tobacco companies use social media influencers to advertise their products on social media and by adverting on social media platforms, they will increase the reach of the adverts to a wider audience. We found that youths had divergent views on e-cigarette marketing on social media. Several participants stated that e-cigarette advertising on social media should be prohibited, citing the high prevalence of youths on social media who could access the content. While others suggested a balanced approach to regulating advertising on social media so that they can still be advertised as cessation devices by smokers. Youths suggested that all adverts should contain warnings (such as age and health warnings) or other restrictive measures (such as parental controls or age restrictions) to protect youths and non-users.

For peer review only

Previous studies which examined e-cigarette-related social media [25-27, 40-44] found that the vast majority of the content depicted positive attitudes towards vaping, while negative characterisations were mostly absent. Our study found that youths had similar experiences when exposed to e-cigarette content on social media. Consequently, there is a risk that youths will be exposed to, and possibly engage with, content that promotes vaping while staying uninformed about the negative aspects, including potential health harms. Our study adds to existing concerns about youths' perceptions of e-cigarettes as cool, fashionable products [30, 45-48], by considering the advertising of the products by influencers on social media platforms, frequently accessed by youths. The use of social media influencers to promote products and celebrity endorsements encourage their followers to share and interact with their content, and ultimately purchase their products. Social media influencers often collaborate with multiple industries (e.g., fashion, and beauty products) in addition to e-cigarette products/brands. These influencers could potentially expose their non-e-cigarette-focused audience (including non-users of e-cigarettes) to e-cigarette content [49]. Therefore, these influencers could be considered an even higher risk for youth compared to influencers who post exclusively about e-cigarettes. This is problematic and concerning as most e-cigarette brands claim that their advertising and promotional content is meant to target current cigarette smokers to help them switch to e-cigarettes, [50, 51] rather than people who do not use nicotine at all. Previous literature has focused on either smokers, non-smokers and/or vapers, leaving out non-vapers and dual-users. Participants in our study were primarily non-smokers and non-vapers, we argue that it is important to explore non-smokers and non-vapers exposure to and engagement with e-cigarette adverts on social media. This is important for helping to determine the potential influence of e-cigarette adverts on social media in non-smokers and non-vapers.

Before plain packaging, the tobacco industry successfully used colourful packaging to represent its brand and increase the appeal of smoking [52-54]. The marketing tactics used by the tobacco industry were so effective that several e-cigarette manufacturers have used the same trade secrets to advertise their products [55-57]. Research has shown the plethora of bright-coloured packaging, eye-catching designs, and variety of flavourings available all appeal to youths and may result in experimentation of the products [58-63].

For peer review only

For peer review only - http://bmjopen.bmj.com/site/about/guidelines.xhtml

Previous research has illustrated YouTube viewers' exposure to e-cigarettes (as well as alcohol and tobacco) while watching videos on the platform [64]. Our study adds to this by illustrating the potential mechanisms by which exposure to e-cigarette advertising on social media platforms may influence perceptions and resultant trial/use of the products. While restrictions on mass media marketing of e-cigarettes are increasingly considered internationally, social media platforms are more difficult to manage because user-generated content (in particular) will not be covered by incoming regulations. Our study findings resonate with previous research, which found that youths were easily able to purchase e-cigarettes from the internet due to the absence of age-verification measures used by internet vendors [65, 66].

As with all research, our study has some limitations, several of which are similar to that detailed in [30] as this research closely followed the same methodological approach First, and consistent with the qualitative design, the sample does not aim to be representative of UK youth, as our study focused on Scottish youths. However, we did have a diverse sample of both sexes. Secondly, the study's geographical remit has to be considered when interpreting the findings. The UK is considered an international leader in tobacco control policy. It is possible that participants' views may have been influenced by the UK's unique favourable policy approach to e-cigarettes and legal and socio-cultural context, including low smoking prevalence. Thirdly, using friendship groups may have influenced youth’s honesty when answering questions and their responses. Youth’s may not have wanted to disagree with their peers, thus responded in a similar way to the rest of the group. Fourthly, participants in this study were primarily non-smokers and non-vapers and it is possible this may have impacted in their discussion of e-cigarette advertisements on social media. However, as we used predominately visual prompts based upon data found during the scoping review of the research [14] we did not find that non-smokers and non-vapers contributed less to discussions. If we had not used visual prompts and focused on participants own experiences of what they had seen on social media (i.e., recalling from memory) this may have impacted non-smokers and non-vapers contribution to discussions. Fifthly, the data was collected in different formats (online and face-to-face), and this may have influenced participants’ responses. Two of the online groups were conducted in a classroom with

For peer review only

a teacher present, and during seven face-to-face groups, a teacher/youth worker was present in the room. The presence of a teacher/youth worker may have influenced youth's willingness to answer questions and their responses. Finally, two of the groups were recruited through personal networks and this may have impacted the youth's responses.

The results from this study should provide guidance for future research. More research is needed to determine the most effective means to counter the favourable/positive aspects of e-cigarettes to reduce youths' interest in product trial and use. Future research could explore if there are different perceptions of e-cigarette exposure in social media between females and males. As females are one of the target groups for e-cigarette use [67, 68], it can reflect the marketing situation of e-cigarettes for females on social media, which deserves further study. In addition, research on the prevalence of warning statements on e-cigarette-related social media posts and the impact this has on youth perceptions would be an important future direction to expand this work.

Considering that most youth access multiple social media platforms multiple times per day [69, 70] and that exposure to this marketing is related to use [71, 72], our findings indicate a significant public health concern. Given the findings presented by our study, there is a growing need for policymakers to develop comprehensive plans to build more robust measures to protect youths and to restrict the ability of marketers to reach youths with enticing social media content promoting e-cigarettes. In addition, social media platforms should consider implementing more robust measures, such as age restrictions and portraying the negative aspects of vaping, to ensure the prevention of vaping-related content targeted at underage users.

CONCLUSION

The tactics used on social media platforms for advertising and marketing e-cigarettes directly to youths raises concerns and a new generation of youths becoming addicted to nicotine. The results of this study highlight that the e-cigarette industry is using previously employed tactics similar to the

For peer review only - http://bmjopen.bmj.com/site/about/guidelines.xhtml

tobacco industry to advertise and promote its products on social media. Most youths use social media daily and the depiction of products on social media represents a key influence on young people's understanding of products. These findings may highlight a priority for governmental policy to restrict the ability of marketers to reach youths with social media content promoting e-cigarettes.

Declarations

Authors' contributions: **Marissa J. Smith:** Conceptualisation, Data Curation, Investigation, Methodology, Validation, Visualisation**,** Writing- Original draft preparation. **Shona Hilton:** Conceptualisation, Methodology, Validation, Writing - Review & Editing.

Ethical approval: Ethical approval for the study was obtained from the University of Glasgow's Medical and Veterinary Life Sciences Ethics Committee (reference 200210034).

Competing interests: The authors declare that they have no competing interests.

Data sharing statement: All data relevant to the study are included in the article or uploaded as supplementary information**.**

Funding: MS acknowledges funding from Cancer Research UK grant PPRCTAGPJT\100003. SH is funded by the Medical Research Council grant MC_UU_00022/1, the Chief Scientist Office of the Scottish Government Health Directorates grant SPHSU17, and Cancer Research UK grant PPRCTAGPJT\100003.

Role of the funding source: The funders of this study had no role in the study design, data analysis, data interpretation, or writing of the report. The corresponding author had full access to all of the data and final responsibility for the decision to submit it for publication.

Acknowledgements: Patients and or public were not involved.

REFERENCES

1. Hammond D, Wackowski OA, Reid JL, O'Connor RJ. Use of JUUL E-cigarettes Among Youth in the United States. Nicotine Tob Res. 2020;22(5):827-32.

For peer review only

2. Duke JC, Allen JA, Eggers ME, Nonnemaker J, Farrelly MC. Exploring Differences in Youth Perceptions of the Effectiveness of Electronic Cigarette Television Advertisements. Nicotine Tob Res. 2016;18(5):1382-86.

3. Farrelly MC, Duke JC, Crankshaw EC, Eggers ME, Lee YO, Nonnemaker JM, et al. A Randomized Trial of the Effect of E-cigarette TV Advertisements on Intentions to Use E-cigarettes. Am J Prev Med. 2015;49(5):686-93.

4. Hammond D, Reid JL, Burkhalter R, Rynard VL. E-cigarette Marketing Regulations and Youth Vaping: Cross-Sectional Surveys, 2017–2019. 2020;146(1).

5. European Commission. Tobacco Products Directive (2014/40/EU). European Commission; 2014. p. 1-38.

6. The Tobacco and Related Products Regulations 2016, 507. Sect. Part 2 (2016).

7. Stead M, Ford A, Angus K, MacKintosh AM, Purves R, Mitchell D. E-Cigarette Advertising in the UK: A Content Analysis of Traditional and Social Media Advertising to Observe Compliance with Current Regulations. Nicotine Tob Res. 2021;23(11):1839-47.

8. Advertising Standards Authority. About the ASA and CAP 2021 [Available from: https://www.asa.org.uk/about-asa-and-cap/about-regulation/about-the-asa-and-cap.html.

9. Advertising Standards Authority. The CAP Code: The UK Code of Non-broadcast Advertising and Direct & Promotional Marketing. London, UK: Advertising Standards Authority; 2016.

10. Advertising Standards Authority. Electronic Cigarettes: Factual vs. Promotional Claims 2018 [Available from: https://www.asa.org.uk/advice-online/electronic-cigarettes-factual-vs-promotional-claims.html.

11. O'Brien EK, Hoffman L, Navarro MA, Ganz O. Social media use by leading US e-cigarette, cigarette, smokeless tobacco, cigar and hookah brands. Tob Control. 2020;29(e1):e87-e97.

12. De Veirman M, Hudders L, Nelson MR. What Is Influencer Marketing and How Does It Target Children? A Review and Direction for Future Research. Front Psychol. 2019;10.

13. Zhu S, Sun J, Binnevie E, Cummins S, Gamst A, Yin L, et al. Four hundred and sixty brands of e-cigarettes and counting: implications for product regulation. Tob Control. 2014;23(3):3-9.

14. Smith MJ, Buckton C, Patterson C, Hilton S. User-generated content and influencer marketing involving e-cigarettes on social media: a scoping review and content analysis of YouTube and Instagram. BMC Public Health. 2023;23(1):530.

15. Chu KH, Colditz JB, Primack BA, Shensa A, Allem JP, Miller E, et al. JUUL: Spreading Online and Offline. J Adolesc Health. 2018;63(5):582-86.

16. Phua J, Jin SV, Hahm JM. Celebrity-endorsed e-cigarette brand Instagram advertisements: Effects on young adults' attitudes towards e-cigarettes and smoking intentions. J Health Psychol. 2017;23(4):550-60.

17. Amin S, Dunn AG, Laranjo L. Social Influence in the Uptake and Use of Electronic Cigarettes: A Systematic Review. Am J Prev Med. 2020;58(1):129-41.

18. de Andrade M, Hastings G, Angus K. Promotion of electronic cigarettes: tobacco marketing reinvented? BMJ. 2013;DOI: 10.1136/bmj.f7473.

19. Fairchild AL, Bayer R, Colgrove J. The Renormalization of Smoking? E-Cigarettes and the Tobacco "Endgame". NEJM. 2013;370(4):293-95.

20. Schneider S, Diehl K. Vaping as a Catalyst for Smoking? An Initial Model on the Initiation of Electronic Cigarette Use and the Transition to Tobacco Smoking Among Adolescents. Nicotine Tob Res. 2016;18(5):647-53.

21. Etter J-F. Gateway effects and electronic cigarettes. Addiction. 2017;113(10):1776-83.

22. Cobb NK, Brookover J, Cobb CO. Forensic analysis of online marketing for electronic nicotine delivery systems. Tob Control. 2015;24(2):128.

23. Amin S, Dunn AG, Laranjo L. Exposure to e-cigarette information and advertising in social media and e-cigarette use in Australia: A mixed methods study. 2020;213:108112.

24. Prochaska JJ, Pechmann C, Kim R, Leonhardt JM. Twitter=quitter? An analysis of Twitter quit smoking social networks. Tob Control. 2012;21(4):447-49.

25. Chen Y, Tilden C, Vernberg DK. Adolescents' interpretations of e-cigarette advertising and their engagement with e-cigarette information: results from five focus groups. Psychol Health. 2020;35(2):163-76.

For peer review only - http://bmjopen.bmj.com/site/about/guidelines.xhtml

26. Scheffels J, Tokle R, Linnansaari A, Rasmussen SKB, Pisinger C. E-cigarette use in global digital youth culture. A qualitative study of the social practices and meaning of vaping among 15-20-year-olds in Denmark, Finland, and Norway. 2023;111:103928.

27. Johnson AC, Mays D, Hawkins KB, Denzel M, Tercyak KP. A Qualitative Study of Adolescent Perceptions of Electronic Cigarettes and Their Marketing: Implications for Prevention and Policy. Child Health Care. 2017;46(4):379-92.

28. Liu J, McLaughlin S, Lazaro A, Halpern-Felsher B. What Does It Meme? A Qualitative Analysis of Adolescents' Perceptions of Tobacco and Marijuana Messaging. Public Health Rep. 2020;135(5):578-86.

29. Pepper JK, McRee AL, Gilkey MB. Healthcare providers' beliefs and attitudes about electronic cigarettes and preventive counseling for adolescent patients. 2014;54(6):678-83.

30. Smith MJ, MacKintosh AM, Ford A, Hilton S. Youth's engagement and perceptions of disposable e-cigarettes: a UK focus group study. 2023;13(3):e068466.

31. Guest G, Bunce A, Johnson L. How Many Interviews Are Enough?:An Experiment with Data Saturation and Variability. 2006;18(1):59-82.

32. Braun V, Clarke V. Thematic analysis. APA handbook of research methods in psychology, Vol 2: Research designs: Quantitative, qualitative, neuropsychological, and biological. APA handbooks in psychology®.DOI: 10.1037/13620-004. Washington, D.C., USA: American Psychological Association; 2012. p. 57-71.

33. Scottish Government. The Scottish Index of Multiple Deprivation 2020 2020 [Available from: https://www.gov.scot/collections/scottish-index-of-multiple-deprivation-2020/.

34. Ge J. Emoji Sequence Use in Enacting Personal Identity. In: Liu L, White R, editors. WWW '19: Companion Proceedings of The 2019 World Wide Web Conference; New York, USA2019. p. 426-38.

35. Ge J, Gretzel U. Emoji rhetoric: a social media influencer perspective. 2018;34(15-16):1272-95.

36. Jacobson J, Hodson J, Mittelman R. Pup-ularity contest: The advertising practices of popular animal influencers on Instagram. 2022;174:121226.

37. Kostygina G, Tran H, Czaplicki L, Perks SN, Vallone D, Emery SL, et al. Developing a theoretical marketing framework to analyse JUUL and compatible e-cigarette product promotion on Instagram. Tob Control. 2022;10.1136/tobaccocontrol-2021-057120:tobaccocontrol-2021-057120.

38. Alpert JM, Jaisle A, Chen H. A content analysis of the promotional strategies employed by e-cigarette brands on Twitter. 2019;36(4):307-21.

39. Struik LL, Dow-Fleisner S, Belliveau M, Thompson D, Janke R. Tactics for Drawing Youth to Vaping: Content Analysis of Electronic Cigarette Advertisements. 2020;22(8):e18943.

40. Malik A, Li Y, Karbasian H, Hamari J, Johri A. Live, Love, Juul: User and Content Analysis of Twitter Posts about Juul. Am J Health Behav. 2019;43:326-36.

41. Allem JP, Dharmapuri L, Unger JB, Cruz TB. Characterizing JUUL-related posts on Twitter. Drug Alcohol Depend. 2018;190:1-5.

42. Cole-Lewis H, Pugatch J, Sanders A, Varghese A, Posada S, Yun C, et al. Social Listening: A Content Analysis of E-Cigarette Discussions on Twitter. J Med Internet Res. 2015;17(10):e243.

43. Ketonen V, Malik A. Characterizing vaping posts on Instagram by using unsupervised machine learning. Int J Med Inform. 2020;10.1016/j.ijmedinf.2020.104223.

44. Smith MJ, Buckton C, Patterson C, Hilton S. A scoping review and content analysis of YouTube and Instagram posts. In press.

45. de Andrade M, Angus K, Hastings G. Teenage perceptions of electronic cigarettes in Scottish tobacco-education school interventions: co-production and innovative engagement through a pop-up radio project. 2016;136(5):288-93.

46. Choi K, Fabian L, Mottey N, Corbett A, Forster J. Young adults' favorable perceptions of snus, dissolvable tobacco products, and electronic cigarettes: findings from a focus group study. Am J Public Health. 2012;102(11):2088-93.

47. Park E, Kwon M, Gaughan MR, Livingston JA, Chang Y-P. Listening to Adolescents: Their Perceptions and Information Sources About E-cigarettes. 2019;48:82-91.

For peer review only - http://bmjopen.bmj.com/site/about/guidelines.xhtml

48. Vogel EA, Ramo DE, Rubinstein ML, Delucchi KL, Darrow SM, Costello C, et al. Effects of Social Media on Adolescents' Willingness and Intention to Use E-Cigarettes: An Experimental Investigation. Nicotine Tob Res. 2020;10.1093/ntr/ntaa003.
49. Vassey J, Valente T, Barker J, Stanton C, Li D, Laestadius L, et al. E-cigarette brands and social media influencers on Instagram: a social network analysis. Tob Control. 2022;10.1136/tobaccocontrol-2021-057053:tobaccocontrol-2021-057053.
50. Dewhirst T. Co-optation of harm reduction by Big Tobacco. Tob Control. 2021;30(e1):e1.
51. Prakash S, Xu Y, Goldenson NI, Wissmann R, Gougelet R, Shiffman S. Transitions in Smoking among Adults Newly Purchasing the JUUL System. Am J Health Behav. 2021;45(3):546-62.
52. Wayne GF, Connolly GN. How cigarette design can affect youth initiation into smoking: Camel cigarettes 1983-93. Tob Control. 2002;11 Suppl 1(Suppl 1):I32-9.
53. Carpenter CM, Wayne GF, Connolly GN. Designing cigarettes for women: new findings from the tobacco industry documents. Addiction. 2005;100(6):837-51.
54. Anderson SJ, Glantz SA, Ling PM. Emotions for sale: cigarette advertising and women's psychosocial needs. Tob Control. 2005;14(2):127-35.
55. Campaign for Tobacco-Free Kids. Campaign for Tobacco-Free Kids. 2013. [cited 2022]. Available from: https://www.tobaccofreekids.org/blog/2013_10_02_ecigarettes.
56. Haugh M. University of Richmond. 2020. [cited 2022]. Available from: https://jolt.richmond.edu/2020/11/27/e-cigarettes-new-technology-but-old-advertising-strategy/.
57. Walley SC, Wilson KM, Winickoff JP, Groner J. A Public Health Crisis: Electronic Cigarettes, Vape, and JUUL. 2019;143(6).
58. Kong G, Morean ME, Cavallo DA, Camenga DR, Krishnan-Sarin S. Reasons for Electronic Cigarette Experimentation and Discontinuation Among Adolescents and Young Adults. Nicotine Tob Res. 2015;17(7):847-54.
59. Audrain-McGovern J, Strasser AA, Wileyto EP. The impact of flavoring on the rewarding and reinforcing value of e-cigarettes with nicotine among young adult smokers. Drug Alcohol Depend. 2016;166(1):263-67.
60. Czoli CD, Goniewicz M, Islam T, Kotnowski K, Hammond D. Consumer preferences for electronic cigarettes: results from a discrete choice experiment. Tob Control. 2016;25(1):30-36.
61. Zare S, Nemati M, Zheng Y. A systematic review of consumer preference for e-cigarette attributes: Flavor, nicotine strength, and type. 2018;13(3):e0194145.
62. Kalaji MNH. The Visual Is Worth Thousands of Words: Adult and Youth Perceptions of Visual Cues in e-Cigarette Advertising [Ph.D.]: Cornell University; 2022.
63. Okawa S, Tabuchi T, Miyashiro I. Who Uses E-cigarettes and Why? E-cigarette Use among Older Adolescents and Young Adults in Japan: JASTIS Study. 2020;52(1):37-45.
64. Cranwell J, Murray R, Lewis S, Leonardi-Bee J, Dockrell M, Britton J. Adolescents' exposure to tobacco and alcohol content in YouTube music videos. Addiction. 2015;110(4):703-11.
65. Williams RS, Derrick J, Ribisl KM. Electronic Cigarette Sales to Minors via the Internet. 2015;169(3):e1563-e63.
66. Schiff S, Liu F, Cruz TB, Unger JB, Cwalina S, Leventhal A, et al. E-cigarette and cigarette purchasing among young adults before and after implementation of California's tobacco 21 policy. Tob Control. 2021;30(2):206.
67. Pike JR, Tan N, Miller S, Cappelli C, Xie B, Stacy AW. The Effect of E-cigarette Commercials on Youth Smoking: A Prospective Study. 2019;43(6):1103-18.
68. Truth Initiative. Old tactics, new products: how big tobacco targets women in e-cigarette marketing 2023 [Available from: https://truthinitiative.org/research-resources/targeted-communities/old-tactics-new-products-how-big-tobacco-targets-women.
69. Statista. Social media sites or apps used by children in the United Kingdom (UK) in 2020 2020 [Available from: https://www.statista.com/statistics/415138/social-media-usage-among-children-uk/#:~:text=Youth%20accounts%20on%20the%20rise,2020%2C%20peaking%20at%2087%20percent.

For peer review only - http://bmjopen.bmj.com/site/about/guidelines.xhtml

70. Statista. Reach of leading social networking sites used by those aged 15 to 25 in the United Kingdom (UK) as of 3rd quarter 2020 2022 [Available from: https://www.statista.com/statistics/1059462/social-media-usage-uk-age/.
71. Depue JB, Southwell BG, Betzner AE, Walsh BM. Encoded exposure to tobacco use in social media predicts subsequent smoking behavior. Am J Health Promot. 2015;29(4):259-61.
72. Hébert ET, Case KR, Kelder SH, Delk J, Perry CL, Harrell MB. Exposure and Engagement With Tobacco- and E-Cigarette-Related Social Media. J Adolesc Health. 2017;61(3):371-77.

For peer review only

For peer review only - http://bmjopen.bmj.com/site/about/guidelines.xhtml

Appendix A: Interview topic guide

During the focus groups images were shown in a PowerPoint presentation. A series of images (e.g., Slides 2-8) were shown and changed at five-second intervals and once all images in that section were shown the group were then asked to discuss these based upon questions in the topic guide.

Part 1: Introduction/background

- First of all, thank you for taking part in this research.
- I'm [researcher name], a research assistant at the University of Glasgow with an interest in tobacco and e-cigarettes.

Key points for consent:

- Can I confirm that you have received the participant information sheet and signed and returned your consent form.
- Just to reiterate, your taking part is voluntary, and you are free to withdraw at any time without giving a reason.
- Your participation will be anonymous. Excerpts from the interview may be quoted verbatim in a report and a paper, but quotations will be anonymised.
- Non-anonymised interview recordings and transcripts will be destroyed securely upon completion of this research, but anonymised transcripts and consent forms will be stored securely by the University of Glasgow for a period of 10 years for the purposes of ensuring research integrity.
- Finally, the study has been approved by the Ethics Committee of the College of Social Sciences at the University of Glasgow.
- Reiterate that I am interested to hear their thoughts and experiences as things on social media are changing so quickly and they will know more than me.

Young people's use of social media

First of all, I am going to ask you about social media and what you use.

Show Slide 1 of the PowerPoint presentation.

For peer review only

1. Do you recognise any of these logos?
    a. PROMPT: Can you tell me what each logo is?
    b. PROMPT: What do you know about each one?
2. Can you tell me about the social media platforms that you use?
    a. Why do you use those?
    b. In what way do you use those?
        i. PROMPT: How do you use them? E.g., 'liking', commenting, sharing?
        ii. PROMPT: What is the purpose of each?
        iii. PROMPT: Whether all are the same in the sense of what you can do on them or whether some are different?
    c. What social media platforms do you like and dislike?
        i. PROMPT: Why?
    d. On a daily basis, how long do you tend to spend on social media?
3. What do you think the term 'social media influencer' means?
    a. What do social influencers do?
    b. How do you know that someone is a social influencer?
    c. Can you tell me of any social media influencers whom you like and dislike?
    d. What products have you seen influencers advertising?
        i. PROMPT: Clothing, makeup?
    e. How do you think they pick the products that they advertise?
    f. Have you ever bought or used products based on what you have seen on social media?
        i. PROMPT: What type of products were those?
    g. Would you ever like, comment on or share an influencer's image if they were advertising something?

<u>E-cigarettes on social media</u>

Now I am going to show a few slides on PowerPoint and after we have been through them, we will chat through what you have seen.

For peer review only - http://bmjopen.bmj.com/site/about/guidelines.xhtml

Show Slides 2-8 of the PowerPoint presentation.

Eight images of social media influencers and user-generated e-cigarette content were shown to participants. We have not included the images used as this would be considered a copyright and ethics issue.

1. What can you remember from the images that you just saw?
2. Do any of these images grab your attention?
   a. PROMPT: Why?
   b. PROMPT: What do you think about the use of colour in the images?
   c. PROMPT: What do you think about the number of likes each picture has?
   d. PROMPT: What do you think about the caption under the images?
3. Did the images have anything in common?
   a. If so, what?
4. Did you recognise anyone in the images?
   a. If so, who?
5. Why do you think these images have been posted?
6. What do you think is the purpose of the images?
7. What do you think the image is trying to say?
   a. PROMPT: How do you know this?
8. Who do you think these images are targeted at?
   a. PROMPT: Why do you say that?
9. Do you think these images are selling a product?
   a. PROMPT: How can you tell if a product is being promoted or not?
   b. PROMPT: How do you know which product(s) are being promoted?
10. Who do you think has posted the images I showed you?
    a. Do you think that person is being paid to post the image or not?
    b. How can you tell if someone is being paid or not?
    c. Why do you think influencers would want to advertise e-cigarette products?
    d. Why do you think companies would pay influencers to advertise their products?

Show slides 9-12 of the PowerPoint presentation.

For peer review only - http://bmjopen.bmj.com/site/about/guidelines.xhtml

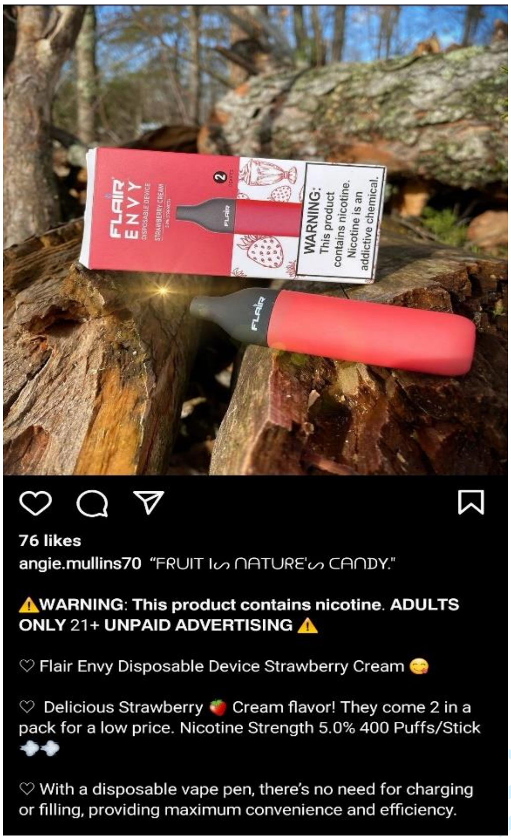

Slide 9

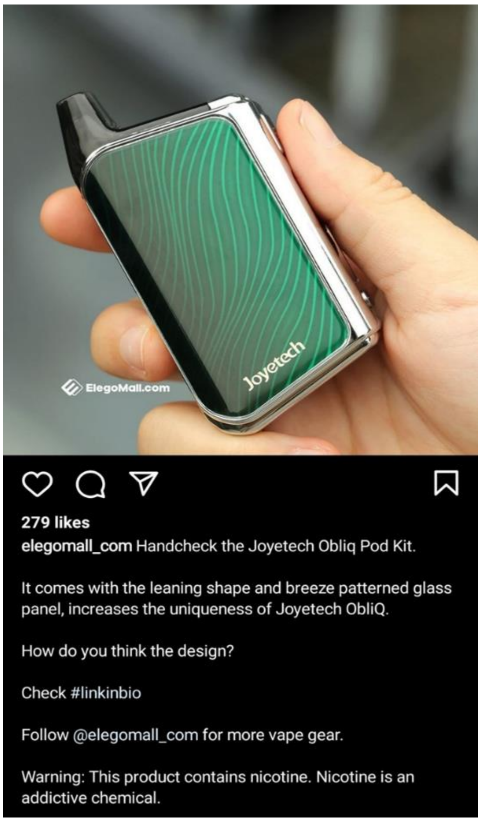

Slide 10

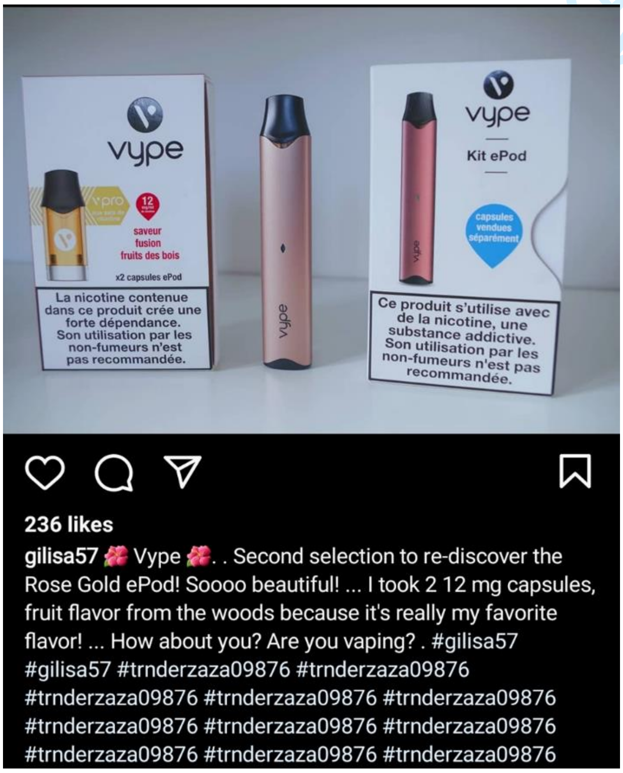

Slide 11

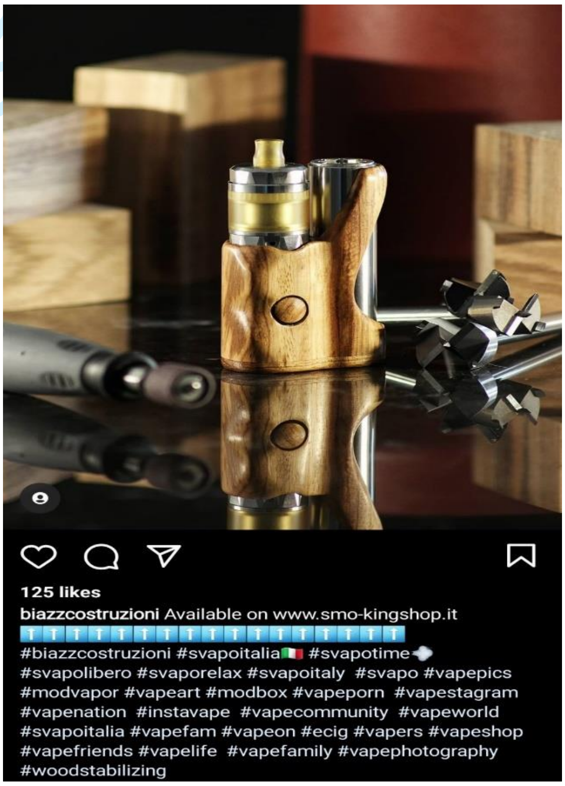

Slide 12

1. What can you remember from the images that you just saw?
2. Do any of these images grab your attention?
    a. PROMPT: Why?
    b. PROMPT: What do you think about the use of colour in the images?
    c. PROMPT: What do you think about the number of likes each picture has?
    d. PROMPT: What do you think about the caption under the images?
3. Did the images have anything in common?
    a. If so, what?
4. Do any of these images remind you of any other products?
    a. PROMPT: Why?
5. Why do you think these images have been posted?
6. What do you think is the purpose of the images?
7. What do you think the image is trying to say?
    a. PROMPT: How do you know this?
8. Who do you think these images are targeted at?
    a. PROMPT: Why do you say that?
9. Why do you think e-cigarettes have been designed this way?

Show slides 14-18 of the PowerPoint presentation.

For peer review only

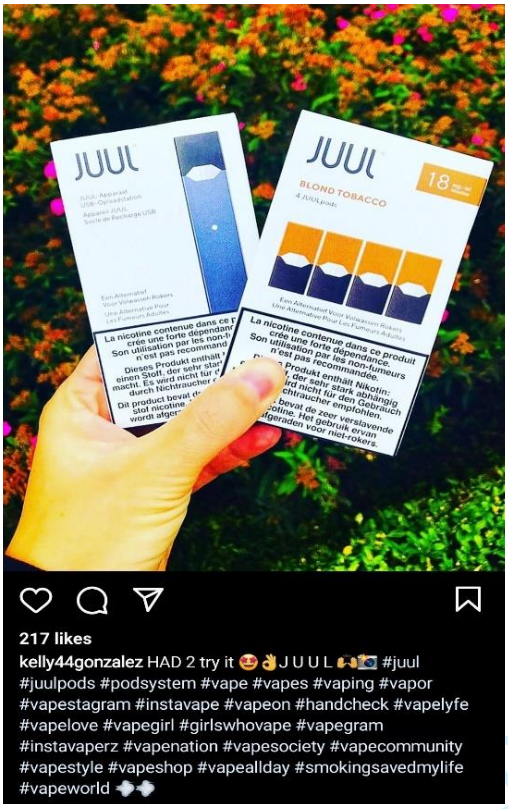

Slide 14

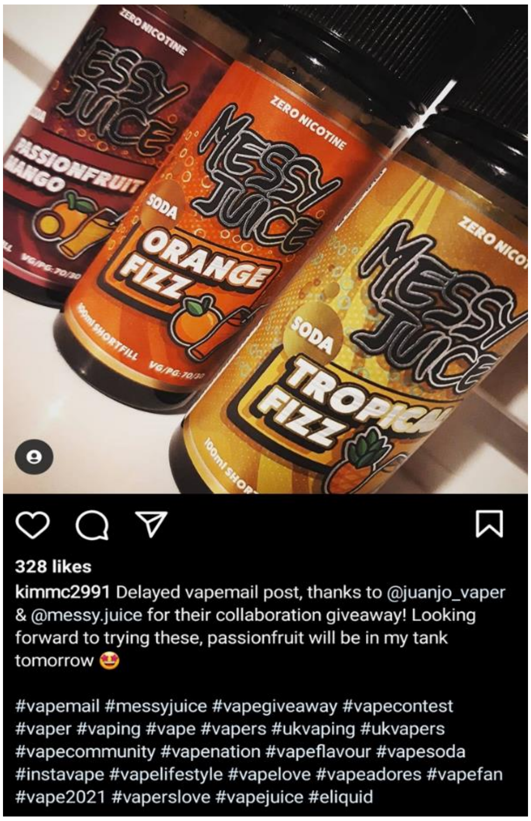

Slide 15

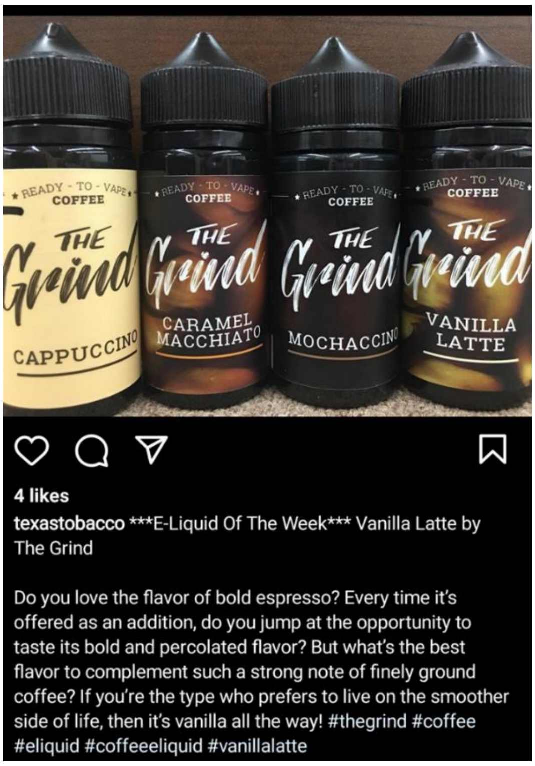

Slide 16

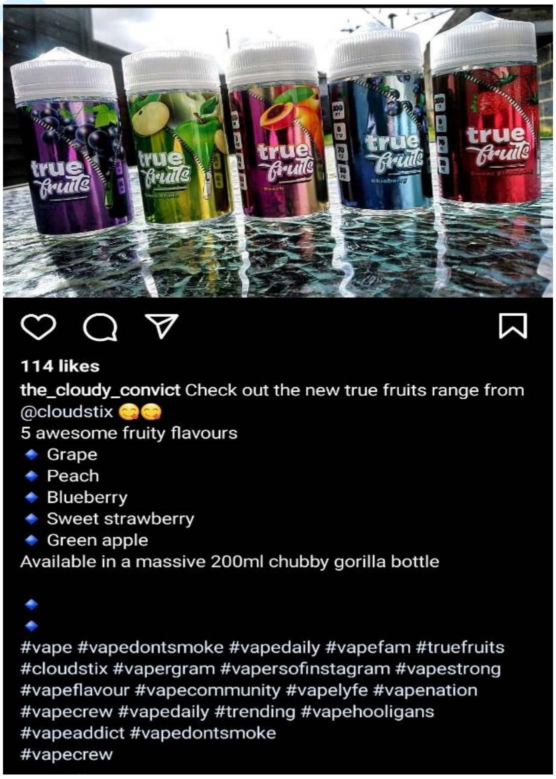

Slide 17

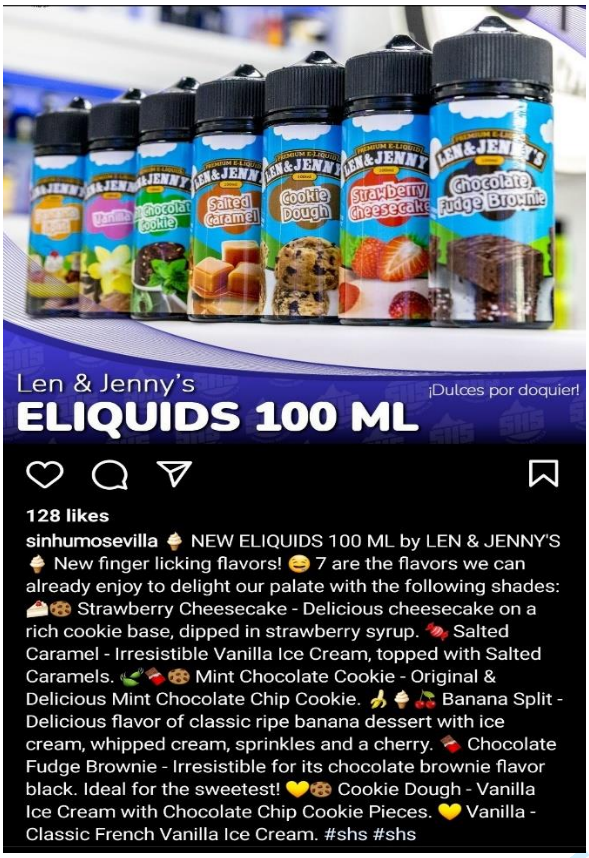

Slide 18

review only

1. What can you remember from the images that you just saw?
2. Do any of these images grab your attention?
    a. PROMPT: Why?
    b. PROMPT: What do you think about the colour of the products?
    c. PROMPT: What do you think about the number of likes each picture has?
    d. PROMPT: What do you think about the caption under the images?
3. Did the images have anything in common?
        i. If so, what?
4. Do any of these images remind you of any other products?
    a. PROMPT: Why?
5. Do you think these images are selling a product?
    a. Do you know what the products are that are shown in the images?
        i. If yes, how do you know that?
6. Why do you think these images have been posted?
7. What do you think is the purpose of the images?
8. What do you think the image is trying to say?
    a. PROMPT: How do you know this?
9. Who do you think these images are targeted at?
    a. PROMPT: Why do you say that?
10. What do you think of the flavours shown?
11. Do you see some flavours as more appealing compared to others?
    a. PROMPT: What ones and why?
12. Do you think there are any flavours that are more appealing to youths compared to adults?
    a. PROMPT: What ones and why?

<u>Personal experiences</u>

I am now moving on to ask about your own experiences and of friends and other people.

1. Have you seen any information about e-cigarettes on social media platforms?
    a. What kind of information was it?
        i. PROMPT: Why do you think the images were posted?
        ii. PROMPT: What do you think the images were trying to show/tell you?
2. Do friends or anyone you follow on social media post or share messages about e-cigarettes?
    a. How does that make you feel about them?
    b. Why do you think they post or share these messages?
    c. If seeing a friend or someone you know post about e-cigarettes on social media, would it make you want to share images or messages?

For peer review only - http://bmjopen.bmj.com/site/about/guidelines.xhtml

d. Out of interest have you or your friends ever posted on social media about e-cigarettes?

Warnings on social media posts

1. Play the video (short clip) https://www.youtube.com/watch?v=tk9SQDH_ZNo
    a. Did you notice anything about the short clip I just showed you?
    b. Can you describe what you remember from the clip I just showed you?
    c. IF NECESSARY PROMPT: Did anyone notice the warning notice?
    d. PROMPT: What did it say?
    e. PROMPT: What did you think about it?
2. Replay the video pointing out the warning.
    a. What do you think about the length of time that the warning was shown for?
        i. How easy or difficult was it to notice the warning?
3. Why do you think there are warnings on posts/videos that show e-cigarette products?
4. Do you think there should or should not be warnings on posts/videos showing e-cigarette products?
    a. PROMPT: Why?

Final remarks

1. Have you ever noticed the marketing/promotion of e-cigarette products before or not?
2. How do you think about the marketing/promotion of e-cigarette products on social media?

Close interview

- Is there anything else you would like to add, that we haven't already talked about?
- Thank you very much for taking part.

For peer review only

Standards for Reporting Qualitative Research (SRQR)*

http://www.equator-network.org/reporting-guidelines/srqr/

	Page/line no(s).
**Title and abstract**	
**Title** - Concise description of the nature and topic of the study Identifying the study as qualitative or indicating the approach (e.g., ethnography, grounded theory) or data collection methods (e.g., interview, focus group) is recommended	1
**Abstract** - Summary of key elements of the study using the abstract format of the intended publication; typically includes background, purpose, methods, results, and conclusions	2
**Introduction**	
**Problem formulation** - Description and significance of the problem/phenomenon studied; review of relevant theory and empirical work; problem statement	4 and 5
**Purpose or research question** - Purpose of the study and specific objectives or questions	5
**Methods**	
**Qualitative approach and research paradigm** - Qualitative approach (e.g., ethnography, grounded theory, case study, phenomenology, narrative research) and guiding theory if appropriate; identifying the research paradigm (e.g., postpositivist, constructivist/ interpretivist) is also recommended; rationale**	6 and 7
**Researcher characteristics and reflexivity** - Researchers' characteristics that may influence the research, including personal attributes, qualifications/experience, relationship with participants, assumptions, and/or presuppositions; potential or actual interaction between researchers' characteristics and the research questions, approach, methods, results, and/or transferability	7, 18 and 19
**Context** - Setting/site and salient contextual factors; rationale**	6 and 7
**Sampling strategy** - How and why research participants, documents, or events were selected; criteria for deciding when no further sampling was necessary (e.g., sampling saturation); rationale**	6 and 7
**Ethical issues pertaining to human subjects** - Documentation of approval by an appropriate ethics review board and participant consent, or explanation for lack thereof; other confidentiality and data security issues	7
**Data collection methods** - Types of data collected; details of data collection procedures including (as appropriate) start and stop dates of data collection and analysis, iterative process, triangulation of sources/methods, and modification of procedures in response to evolving study findings; rationale**	6 and 7
**Data collection instruments and technologies** - Description of instruments (e.g., interview guides, questionnaires) and devices (e.g., audio recorders) used for data collection; if/how the instrument(s) changed over the course of the study	6 and 7
**Units of study** - Number and relevant characteristics of participants, documents, or events included in the study; level of participation (could be reported in results)	6 and 7

For peer review only - http://bmjopen.bmj.com/site/about/guidelines.xhtml

**Data processing -** Methods for processing data prior to and during analysis, including transcription, data entry, data management and security, verification of data integrity, data coding, and anonymization/de-identification of excerpts	6 and 7
**Data analysis -** Process by which inferences, themes, etc., were identified and developed, including the researchers involved in data analysis; usually references a specific paradigm or approach; rationale**	6 and 7
**Techniques to enhance trustworthiness -** Techniques to enhance trustworthiness and credibility of data analysis (e.g., member checking, audit trail, triangulation); rationale**	6 and 7

Results/findings

**Synthesis and interpretation -** Main findings (e.g., interpretations, inferences, and themes); might include development of a theory or model, or integration with prior research or theory	8-15
**Links to empirical data -** Evidence (e.g., quotes, field notes, text excerpts, photographs) to substantiate analytic findings	8-15

Discussion

**Integration with prior work, implications, transferability, and contribution(s) to the field -** Short summary of main findings; explanation of how findings and conclusions connect to, support, elaborate on, or challenge conclusions of earlier scholarship; discussion of scope of application/generalizability; identification of unique contribution(s) to scholarship in a discipline or field	15-19
**Limitations -** Trustworthiness and limitations of findings	18 and 19

Other

**Conflicts of interest -** Potential sources of influence or perceived influence on study conduct and conclusions; how these were managed	20
**Funding -** Sources of funding and other support; role of funders in data collection, interpretation, and reporting	20

*The authors created the SRQR by searching the literature to identify guidelines, reporting standards, and critical appraisal criteria for qualitative research; reviewing the reference lists of retrieved sources; and contacting experts to gain feedback. The SRQR aims to improve the transparency of all aspects of qualitative research by providing clear standards for reporting qualitative research.

**The rationale should briefly discuss the justification for choosing that theory, approach, method, or technique rather than other options available, the assumptions and limitations implicit in those choices, and how those choices influence study conclusions and transferability. As appropriate, the rationale for several items might be discussed together.

Reference:

O'Brien BC, Harris IB, Beckman TJ, Reed DA, Cook DA. **Standards for reporting qualitative research: a synthesis of recommendations.** *Academic Medicine*, Vol. 89, No. 9 / Sept 2014

DOI: 10.1097/ACM.0000000000000388

For peer review only - http://bmjopen.bmj.com/site/about/guidelines.xhtml

For peer review only

For peer review only - http://bmjopen.bmj.com/site/about/guidelines.xhtml
